# MADS-Box and bHLH Transcription Factors Coordinate Transmitting Tract Development in *Arabidopsis thaliana*

**DOI:** 10.3389/fpls.2020.00526

**Published:** 2020-05-06

**Authors:** Maurizio Di Marzo, Irma Roig-Villanova, Eva Zanchetti, Francesca Caselli, Veronica Gregis, Paola Bardetti, Matteo Chiara, Andrea Guazzotti, Elisabetta Caporali, Marta Adelina Mendes, Lucia Colombo, Martin M. Kater

**Affiliations:** Dipartimento di Bioscienze, Università degli Studi di Milano, Milan, Italy

**Keywords:** bHLH, cell death, cell wall, extracellular matrix, MADS-box, transmitting tract

## Abstract

The MADS-domain transcription factor *SEEDSTICK* (*STK*) controls several aspects of plant reproduction. *STK* is co-expressed with *CESTA* (*CES*), a basic Helix-Loop-Helix (bHLH) transcription factor-encoding gene. *CES* was reported to control redundantly with the brassinosteroid positive signaling factors BRASSINOSTEROID ENHANCED EXPRESSION1 (BEE1) and BEE3 the development of the transmitting tract. Combining the *stk ces-4* mutants led to a reduction in ovule fertilization due to a defect in carpel fusion which, caused the formation of holes at the center of the septum where the transmitting tract differentiates. Combining the *stk* mutant with the *bee1 bee3 ces-4* triple mutant showed an increased number of unfertilized ovules and septum defects. The transcriptome profile of this quadruple mutant revealed a small subset of differentially expressed genes which are mainly involved in cell death, extracellular matrix and cell wall development. Our data evidence a regulatory gene network controlling transmitting tract development regulated directly or indirectly by a STK-CES containing complex and reveal new insights in the regulation of transmitting tract development by bHLH and MADS-domain transcription factors.

## Introduction

The plant life cycle in Angiosperms is characterized by the alternation of diploid sporophyte and haploid gametophyte generations. The sporophyte produces spores, which then develop into gametophytes. The gametophytes produce either the male or the female gametes. Sexual reproduction requires the delivery of sperm nuclei, via the pollen tube, to the embryo sac, where fertilization occurs, and the new diploid sporophyte is formed.

The growing of the pollen tube through the female reproductive organ tissues in Angiosperms is a crucial step in plant sexual reproduction. In *Arabidopsis thaliana*, the pistil or gynoecium results from the fusion of two carpels. The apical part of the pistil is formed by the stigma and the stigma papillae, which are needed to attach the pollen. The stigma is connected by the style to the ovary, in which lodge the ovules. The septum divides the Arabidopsis ovary into two locules. At the center of the septum, as in the style, the transmitting tract tissue develops. Pollen tubes must travel through several distinct tissues before reaching ovules, including the stigma and the transmitting tract both in the style and in the septum (Lord and Russell, 2002; Roeder and Yanofsky, 2006).

The transmitting tract differentiates from the carpel margin meristem (CMM), a meristematic tissue that develops as two internal crests that, fuse when they reach each other in the middle of the young pistil at developmental stage 9. The transmitting tract is fully developed at stage 12 of pistil development (Bowman et al., 1999; Roeder and Yanofsky, 2006; Reyes-Olalde et al., 2013). The cells composing the transmitting tract secrete an extracellular matrix (ECM), a complex mixture of polysaccharides, glycoproteins, and glycolipids that accompany the growing pollen tubes (Lennon et al., 1998). Indeed, the pollen tubes grow faster, longer and more precisely *in vivo* than *in vitro*, suggesting the importance of the ECM for pollen tube growth in response to female signal factors (Johnson and Preuss, 2002; Johnson and Lord, 2006; Kim et al., 2006). Moreover, programmed cell death (PCD) in the transmitting tract is critical for pollen tube growth as well. The PCD of the transmitting tract starts before anthesis and can be accelerated by pollination (Crawford et al., 2007).

A complex genetic network controls the development of the transmitting tract. The zinc-finger transcription factor NO TRANSMITTING TRACT (NTT) has pivotal roles in this process. In the *ntt* mutant, the PCD of the cells composing the transmitting tract during fertilization, which is also influenced by the ECM composing cell wall, is severely compromised (Crawford et al., 2007). The changes in PCD and ECM in the *ntt* mutant cause a partial failure in pollen tube growth, with a high percentage of unfertilized ovules from the middle to the bottom part of the siliques (Crawford et al., 2007). NTT acts upstream of three genes that work redundantly in the control of the style and transmitting tract development, that are *CESTA* (*CES*), also named *HALF FILLED* (*HAF*), a bHLH-encoding transcription factor, and two phylogenetically related genes *BRASSINOSTEROID ENHANCED EXPRESSION1* (*BEE1*) and *BEE3* (Crawford and Yanofsky, 2011; Poppenberger et al., 2011).

*BEE*s expression and activity are induced in response to brassinosteroids (Friedrichsen et al., 2002). BRASSINOSTEROID INSENSITIVE 2 (BIN2) directly represses CES activity by phosphorylating it and thus promoting its degradation, restricting its subcellular localization and inhibiting the DNA binding activity of this transcription factor (Vert et al., 2005; Kim and Wang, 2010; Poppenberger et al., 2011). In response to brassinosteroids, phosphorylation of CES is inhibited and the unphosphorylated isoform accumulates promoting its SUMOylation (Khan et al., 2014). It is hypothesized that the brassinosteroid-induced SUMOylation of CES may facilitate the activation of some target genes involved in plant resistance to freezing stress (Eremina et al., 2016).

The triple mutant *ces* (*haf*) *bee1 bee3* shows similar defects in transmitting tract development as Crawford and collaborators previously observed in the *ntt* mutant (Crawford and Yanofsky, 2011). Furthermore, ectopic expression of *CES* (*HAF*) increases the diameter of the transmitting tract allowing more pollen tubes to pass through it simultaneously (Crawford and Yanofsky, 2011).

Recently, NTT was found to interact with the MADS-domain transcription factor SEEDSTICK (STK) to control transmitting tract development (Herrera-Ubaldo et al., 2019). STK was previously identified for its role in ovule development and acts redundantly with other two MADS-box transcription factors SHATTERPROOF1 (SHP1) and SHP2 (Pinyopich et al., 2003).

*NTT* and *STK* have similar expression patterns in the medial domain of the pistil (Herrera-Ubaldo et al., 2019). At anthesis, the *ntt stk* double mutant presents a number of cells composing the septum similar to wild type (Herrera-Ubaldo et al., 2019). However, in the *ntt stk* mutant siliques, there is a partial failure in septum fusion, and it appears to have holes. The fact that the cell number composing the septum has not changed suggests that the abnormal fusion is not related to early stages of development but rather to later defects during epidermis formation (Herrera-Ubaldo et al., 2019). The partial failure of pollen tube growth in the *ntt stk* mutant is also caused by the absence of degeneration and collapse of the cells composing the septum and consequently the transmitting tract (Herrera-Ubaldo et al., 2019).

Mendes et al. (2016) performed a bioinformatic analysis to identify genes with expression patterns similar to *STK*, using Arabidopsis Affymetrix microarray data from 2000 independent experiments. From this experiment *CES* was identified as one of the best candidates to participate in the same processes as STK.

Here we report a genetic and molecular analysis which revealed interactions between the AGAMOUS family members *STK*, *SHP1*, and *SHP2*, the bHLH gene *CES* and the closely related genes *BEE1* and *BEE3* in the control of transmitting tract development in Arabidopsis. We further performed a transcriptome analysis of the *bee1 bee3 stk ces-4* quadruple mutant uncovering new downstream players, involved in transmitting tract differentiation.

## Materials and Methods

### Plant Material and Growth Conditions

All plants used in this study were in the Columbia (Col-0) ecotype. For all analyses, *Arabidopsis thaliana* seeds were incubated for 2 days at 4°C after sowing. Plants were grown at 22°C under long day conditions (LD). The *ces-4* mutant was obtained from NASC (SAIL_674_A01 lines). This line phenocopy the *ces-3* (*haf*), *ces-1* and *ces-2* mutant alleles that have been previously characterized (Crawford and Yanofsky, 2011; Eremina et al., 2016). The *stk*, *shp1*, and *shp2* mutants were provided by M. Yanofsky (Pinyopich et al., 2003), the *bee1 bee2 bee3* triple mutant was provided by J. Chory (Friedrichsen et al., 2002). The other mutant combinations analyzed were obtained by crossing. The *pCES::GUS* line was provided by Prof. B. Poppenberger (Poppenberger et al., 2011).

### Mutant Genotyping

Genotyping of the mutants was done by PCR analysis. All the primers used in this study are listed in Supplementary Table S11.

### *In situ* Hybridization Analysis

*Arabidopsis* flowers and siliques were fixed and embedded in paraffin as previously described in Dreni et al. (2011). Tissue sections (8 μm) were hybridized with a *CES* specific antisense RNA probe and observed with a Zeiss Axiophot D1 microscope. Images were captured on an Axiocam MRc5 camera (Zeiss) using the Axiovision program (version 4.1).

### Gus-Staining Analysis

GUS staining of *pCES::GUS* was performed as described in Balanzà et al. (2016).

### Silique Analysis

The percentage of unfertilized ovules per fruit was determined by opening at least 20 mature siliques for each genotype, counting the number of unfertilized ovules and the total number of developed seeds per silique under a stereomicroscope. The statistical significance of the differences in unfertilized ovules was analyzed using an Anova test followed by the Tukey HSD test (^∗∗^*p* < 0.01). All the experiments were repeated four times. For each experiment 5 siliques were collected from 4 independent plant lines of the same genotype.

### Scanning Electron Microscope (SEM) Pictures

Biological samples were collected and fixed overnight at 4°C in FAA solution (3.7% formaldehyde, 5% acetic acid and 50% ethanol). Fixed tissues were washed with water and post-fixed with aqueous 2% osmium tetroxide for 2 h at room temperature. Tissues were rinsed several times in deionized water and dehydrated in a graded series of ethanol for 15 min per rinse. This step was followed by critical point drying with liquid CO_2_ and sputter-coating with gold in a Nanotech sputter coater. Specimens were analyzed using a LEO 1430 Scanning Electron Microscope.

### Pollen Tube Guidance Analysis

Experiments of pollen tube guidance were performed as previously described by Mendes et al. (2016) and images were captured on an Axiocam MRc5 camera (Zeiss) using the Axiovision program (version 4.1).

### Bimolecular Fluorescence Complementation Assay (BiFC)

Coding Sequences (CDS) of *CES* and *STK* were amplified by PCR with the primers indicated in Supplementary Table S11 and first cloned into pDONOR207 (Life Technologies) and subsequently into the pYFPN43 and pYFPC43 vectors.^1^ BiFC experiments were conducted as previously described in Balanzà et al. (2016). Essentially, the abaxial surfaces of tobacco leaves were observed 2–5 days after agroinfiltration and the interactions were monitored using a Leica TCS confocal microscope.

### Yeast Two-Hybrid Assays

The two-hybrid assays were performed at 22°C in the yeast strain AH109 (Clontech), using the cotransformation technique (Egea-Cortines et al., 1999). The coding sequences of *CESTA*, *STK*, and *BEE1* were cloned in the Gateway vector GAL4 system (pGADT7 and pGBKT7; Clontech) passing through pDONOR207 (Life Technologies). Yeast two-hybrid interaction assays were performed on selective yeast synthetic dropout medium lacking Leu, Trp, Adenine, and His supplemented with different concentrations of 3-aminotriazole (1, 2.5, 5, 10, and 15 mM of 3-AT).

### RNA Extraction and qRT-PCR Analysis

For the qRT-PCR experiment of *ces-4* (knockout validation) total RNA was extracted from whole inflorescences using the LiCl method as previously described (Verwoerd et al., 1989). First-strand cDNA was synthesized using an IMProm-II ^TM^ Reverse Transcription System (Promega). Enrichments fold were detected using a SYBER Green assay (Bio-Rad).^2^ The qRT-PCR assay was performed in triplicate using a Bio-Rad iCycler iQ optical system (software version 3.0a). The qRT-PCR assay was repeated three times. For each experiment the RNA was extracted from three independent plant lines.

### RNA Sequencing

Total RNA was extracted from three biological replicates (1 gr, obtained from a pool of 5 plants for each replicate) from both wild-type and *bee1 bee3 stk ces-4* mutant inflorescences till stage 12, using the Macherey Nagel “Nucleospin RNA Plant” according to the manufacturer’s instructions. RNA concentrations and integrity were determined using Qubit Fluorometer and the Qubit^TM^ RNA XR Assay Kit (Thermo Fisher Scientific). Sequencing libraries were prepared using the NEBNext Ultra II Directional RNA library Prep Kit for Illumina (NEB) according to the manufacture’s instruction and sequenced on the HiSeq Illumina platform. Reads were mapped on the reference *Arabidopsis thaliana* genome (TAIR, version 10) using the bowtie2 program (Langmead and Steven, 2012; Supplementary Table S4). Estimation of gene expression levels was performed using RSEM (Li and Dewey, 2011). Identification of differentially expressed genes was performed by the quasi-likelihood *F*-test as implemented by edgeR (Robinson et al., 2009). A False Discovery Rate (FDR) cut-off value of 0.05 was applied for the identification of significantly differentially expressed genes. Graphical representation of the data was performed by means of the gplots R library. To validate the data obtained from the RNAseq experiment, we extracted total RNA from wild type and *bee1 bee3 stk ces-4* mutant inflorescences till stage 12, using the Macherey Nagel “Nucleospin RNA Plant” according to the manufacturer’s instructions. The cDNA and qRT-PCR were performed as described in the previous paragraph “RNA extraction and qRT-PCR analysis.” The qRT-PCR assay to validate RNAseq experiment was repeated three times. For each experiment the RNA was extracted from three independent plant lines that were used for RT-PCR analysis.

### TFBS Analysis

Enrichment analysis of Transcription Factor Binding Sites (TFBS) was performed by means of the Pscan tool, using the “non-redundant” set of Jaspar TFBS matrices (Fornes et al., 2019) and considering a region spanning from the TSS to 500 bp upstream (default settings). Archetype consensus sites for the W-box, MADS-box and G-box elements were established as the Jaspar TFBS matrix showing the highest enrichment in our dataset according to Pscan. To identify genes with high confidence W-box, MADS-box and G-box elements in their promoters, the complete set of 33239 Arabidopsis transcripts according to the TAIR10 annotation were scanned using the highly significantly enriched TFBS matrices as defined above. Distribution of scores were established for each element, and an Expectation-Maximization algorithm as implemented in the R EM-cluster package was used to fit a 2 Gaussian mixture model. Based on the EM model promoters were partitioned in “high” and “low” scoring. Only promoters that were confidently assigned to high scoring clusters (confidence > 95%) were considered to have a bona fide TFBS element. Similarly, to our previous analyses promoters were defined as regions spanning 500 bp upstream of the TSS. *P*-values for the enrichment of genes with W-box, MADS-box and G-box elements in their promoters were performed by means of the hypergeometric distribution test as implemented in the R stats package.

### Accession Numbers of the Genes

The GenBank/EMBL accession numbers for the genes shown in this study are the following: *BEE1*, At1g18400; *BEE2*, At4g36540; *BEE3*, At1g73830; *CES*, At1g25330; *SHP1*, At3g58780; *SHP2*, At2g42830; *STK*, At4g09960. The accession number of the genes identified in the transcriptomic analysis are indicated in Supplementary Tables S5, S6.

## Results

### SEEDSTICK and CESTA Are Co-expressed

In order to identify new targets, partners and/or regulators of *STK*, a bioinformatic analysis was previously performed in the laboratory to discover genes co-expressed with *STK* (Mendes et al., 2016). This kind of analysis assumes that correlation between the patterns of gene expression in a large range of different experimental conditions could indicate a functional relationship (Aoki et al., 2007). As already described, the procedure to identify genes that have expression profiles that correlate with *STK* was proven successful since among the top listed genes *SHP1* and *SHP2* were found, which act redundantly with STK in the control of ovule identity (Pinyopich et al., 2003). Furthermore, top rated were *VERDANDI* (*VDD*) and *VALKYRIE* (*VAL*), targets of the STK-SEPALLATA3 (SEP3) complex, which both act in the control of the death of the synergid cell that is receptive of the pollen tube (Mendes et al., 2016). *CES* (At1g25330) rated in position 14 of the correlators, having an expression correlation with *STK* of p(LIN) 0.759 and p(LOG) 0.505 (Mendes et al., 2016).

**FIGURE 1:**
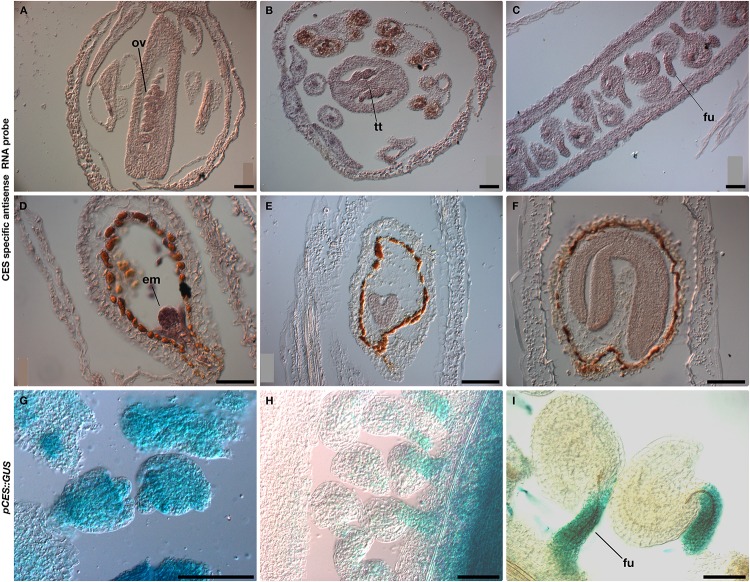
Analysis of *CES* expression. *In situ* hybridization **(A–F)** and GUS activity of the *pCES::GUS* marker line **(G–I)** showed *CES* expression in ovules **(A–C,G–I)**, transmitting tract **(B)**, and in the embryos of the developing seeds **(D–F)**. Em, embryo; fu, funiculus; ov, ovule; tt, transmitting tract. Scale bars: 50 μm.

Previously, it has been reported that *CES* is expressed from stage 8 to 15 of flower development, first in CMM and later in the stigma, septum and funiculi (Crawford and Yanofsky, 2011; Poppenberger et al., 2011). Since *STK* is expressed during female reproductive organ development (mainly septum, transmitting tract, replum, funiculus and ovule/seed) (Pinyopich et al., 2003; Mizzotti et al., 2014; Herrera-Ubaldo et al., 2019; Di Marzo et al., 2020), we investigated in more detail the spatio-temporal expression pattern of *CES* during pistil, ovule and seed development. We performed both RNA *in situ* hybridization and the analysis of the *pCES::GUS* reporter gene construct (Figure 1). The RNA *in situ* analysis experiments showed that *CES* was expressed in ovules during early stages of development until mature ovule formation and its transcripts were also present in the transmitting tract before fertilization (Figures 1A–C). In mature ovules, a high expression was detected in the funiculus (Figure 1C). After fertilization and during embryo development *CES* mRNA was present at high levels in the early globular stage, and at heart and torpedo stages mRNA levels were lower (Figures 1D–F). Analysis of the *pCES::GUS* plants confirmed the *in situ* data (Figures 1G–I) and these experiments showed that *STK* and *CES* partially overlap in their expression patterns during ovule and transmitting tract development (Pinyopich et al., 2003; Crawford and Yanofsky, 2011; Poppenberger et al., 2011; Herrera-Ubaldo et al., 2019).

### *Arabidopsis* Plants With *stk, shp, ces*, and *bee* Mutant Combinations Are Partially Sterile

**FIGURE 2:**
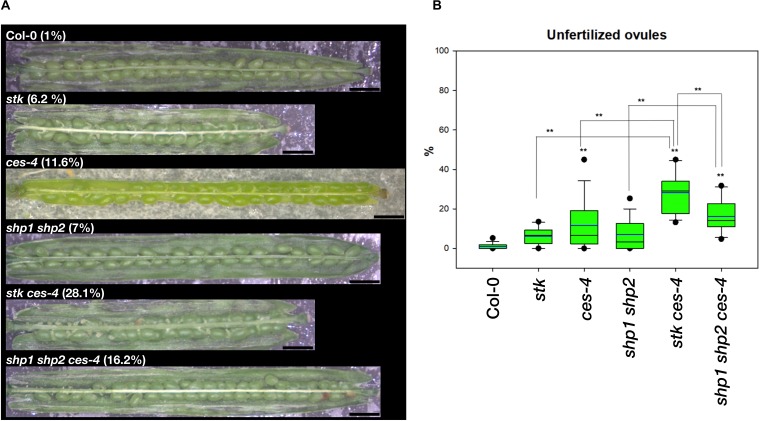
Unfertilized ovules analysis in multiple mutant combination of *stk*, *ces-4*, and *shp1 shp2*. **(A)** Stereomicroscope images of opened siliques of the *stk and ces-4* single mutants, the *stk ces-4* and the *shp1 shp2* double mutants, the *shp1 shp2 ces-4* triple mutant and the wild type Col-0; Scale bars: 1 mm. **(B)** Box plots of all the mutants analyzed in comparison to wild type Col-0. Statistical analysis was performed using Anova followed by Tukey HSD test (**p < 0.01). The experiment was repeated four times. For each experiment 5 siliques were collected from 4 independent plant lines of the same genotype to obtain a total of 20 analyzed siliques.

To further analyze the role of CES, we characterized a CES loss-of-function allele that we obtained from the SAIL collection (Alonso and Stepanova, 2003). This allele, that we called *ces-4*, has a T-DNA insertion in the second exon, differently from *ces-1, ces-2*, and *ces-3* (*haf*), previously published T-DNA knockout insertion alleles (Crawford and Yanofsky, 2011; Poppenberger et al., 2011; Eremina et al., 2016). The qRT-PCR analysis performed to check *CES* expression in the *ces-4* mutant indicated that this is also a null allele (Supplementary Figure S1). Analysis of *ces-4* homozygous plants did not reveal any general phenotype at the level of the entire plant, which was consistent with previous reports for the *ces* (*haf*) mutant alleles (Crawford and Yanofsky, 2011; Poppenberger et al., 2011; Eremina et al., 2016). We crossed the *ces-4* mutant with *stk* to obtain the *stk ces-4* double mutant. The double mutant was indistinguishable from wild type plants apart from a large number of unfertilized ovules at the lower part of the siliques. In wild type plants on average 1.0% of the ovules in a silique remained unfertilized. In the *stk* mutant we observed on average 6.2% unfertilized ovules, and in the *ces-4* single mutant 11.6% (^∗∗^*p* < 0.01 in respect to wild type; Figure 2 and Supplementary Table S1). In the *stk ces-4* double mutant this phenotype was further increased to 28.1% of unfertilized ovules (Figure 2 and Supplementary Table S1). Like in the *ces-4* single mutant, also in the *stk ces-4* double mutant the unfertilized ovules were located always at the lower part of the siliques (Figure 2 and Supplementary Table S1).

Since SHP1 and SHP2 are closely related to STK and have shown to control together with STK ovule identity redundantly (Pinyopich et al., 2003), we investigated whether they also play a role together with CES in the control of ovule fertilization. Therefore, the *shp1 shp2* double mutant was crossed with the *ces-4* mutant to obtain the *shp1 shp2 ces-4* triple mutant. The *shp1 shp2* double mutant showed on average 7.0% unfertilized ovules (Figure 2 and Supplementary Table S1). The *shp1 shp2 ces-4* triple mutant showed a clear increase to 16.2%, also in this case the unfertilized ovules were localized at the lower part of the siliques (^∗∗^*p* < 0.01 in respect to wild type and *shp1 shp2*; Figure 2 and Supplementary Table S1). Unfortunately we could not analyze the *stk shp1 shp2* triple mutant, because the triple mutant does not develop normal ovules (Pinyopich et al., 2003). These results suggest that the MADS-domain transcription factor STK, SHP1 and SHP2 have a role together with CES in the control of ovule fertilization.

**FIGURE 3:**
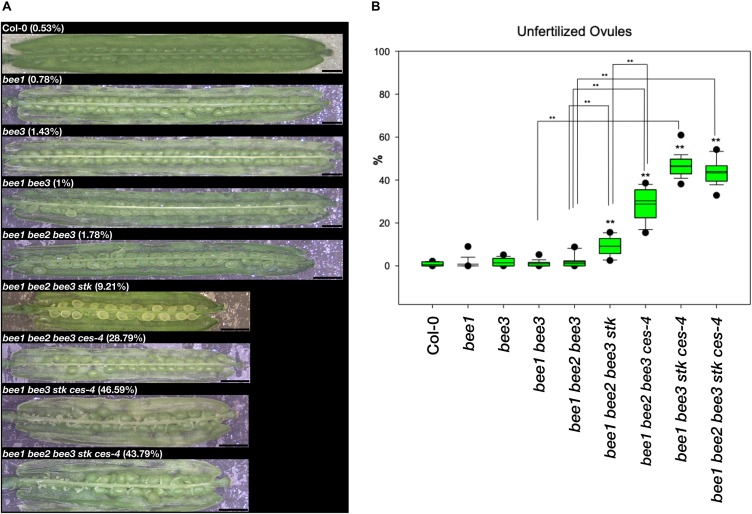
Unfertilized ovules analysis in multiple mutant combination of *bee1*, *bee2*, *bee3*, *stk*, and *ces-4*. **(A)** Stereomicroscope images of opened siliques of the *bee1* and *bee3* single mutants, the *bee1 bee3* double mutant, the *bee1 bee2 bee3* triple mutant, the *bee1 bee2 bee3 stk*, the *bee1 bee2 bee3 ces-4*, and *bee1 bee3 stk ces-4* quadruple mutants, the *bee1 bee2 bee3 stk ces-4* quintuple mutants and the wild type Col-0; Scale bars: 1 mm. **(B)** Box plots of all the mutants analyzed in comparison to wild type Col-0. Statistical analysis was performed using Anova followed by Tukey HSD test (**p < 0.01). The experiment was repeated four times. For each experiment 5 siliques were collected from 4 independent plant lines of the same genotype to obtain a total of 20 analyzed siliques.

The contribution of the *BEE* genes to this process was also investigated (Figure 3 and Supplementary Table S2). *CES* is phylogenetically related to *BEE1* and *BEE3*, which have been described as redundant with *BEE2* (Friedrichsen et al., 2002). Moreover, it has been shown that CES together with BEE1 and BEE3 redundantly control the development of the transmitting tract in the pistil (Crawford and Yanofsky, 2011; Poppenberger et al., 2011). The single mutants *bee1* and *bee3* and the multiple mutant combinations *bee1 bee3* and *bee1 bee2 bee3* did not show any obvious phenotype (Figure 3 and Supplementary Table S2). To investigate redundancy between *BEE* genes and *STK*, we obtained the *bee1 bee2 bee3 stk* quadruple mutant, that presented 9.2% of unfertilized ovules, with ^∗∗^*p* < 0.01 when compared to wild type and the triple mutant *bee1 bee2 bee3*, which again were preferentially positioned at the lower part of the siliques (Figure 3 and Supplementary Table S2). This percentage is lower than the *bee1 bee2 bee3 ces-4* quadruple mutant, which presented in average 28.7% of unfertilized ovules (Figure 3 and Supplementary Table S2). Despite the weaker phenotype, our results demonstrated that STK and BEEs also have a role together in ovule fertilization. To investigate if combining the *ces-4* mutant could further enhance the unfertilized ovule phenotype, we generated the *bee1 bee3 stk ces-4* quadruple and the *bee1 bee2 bee3 stk ces-4* quintuple mutants. The results of this analysis showed that *bee1 bee3 stk ces-4* had on average 46.6% unfertilized ovules, and a similar result was obtained in the *bee1 bee2 bee3 stk ces-4* (44.0%) (Figure 3 and Supplementary Table S2), showing that the addition of the *bee2* allele did not further enhance the phenotype. The unfertilized ovules in these cases are localized from the middle to the bottom part of the siliques. Altogether, these results indicated that the MADS-domain transcription factors function not only together with CES, but also with the BEEs in the control of the fertilization of all the ovules within the Arabidopsis fruit.

### STK and CES Regulate Transmitting Tract Development

To better understand the defect that caused the presence of a high percentage of unfertilized ovules at the lower part of the siliques in the different mutant combinations, we performed reciprocal crosses between wild type and *stk ces-4* or *shp1 shp2 ces-4* mutant plants, to analyze if the female, male or both parts were defective. The outcome of the reciprocal crosses shown in Supplementary Figure S2, suggested that the unfertilized-ovules phenotype was only present when the mutant genotypes were used as mothers. This result indicates that the defects resides only in the female reproductive organ.

To further investigate the defect present in the female reproductive organs, which caused the high number of unfertilized ovules at the lower part of the siliques in the multiple mutant combinations, we analyzed the septum morphology by Scanning Electron Microscope (SEM), at stage 17-B of fruit development, when the siliques reach their maximum size (Roeder and Yanofsky, 2006).

**FIGURE 4:**
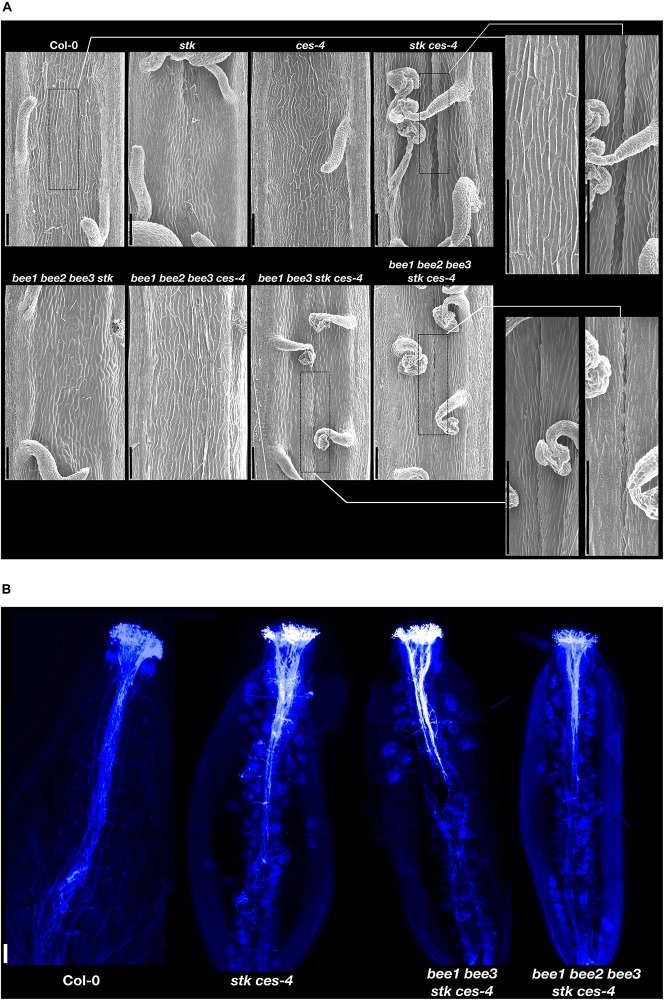
Septum analysis and pollen tube growth. **(A)** Scanning Electron Microscope pictures of the septum of opened siliques of *stk*, *ces-4*, *stk ces-4*, *bee1 bee2 bee3 stk*, *bee1 bee2 bee3 ces-4*, *bee1 bee3 stk ces-4* and *bee1 bee2 bee3 stk ces-4* and wild type Col-0; Scale bars: 100 μm. **(B)** Aniline blue staining of *stk ces-4, bee1 bee3 stk ces-4* and *bee1 bee2 bee3 stk ces-4* and wild type Col-0; Scale bar: 50 μm.

The *stk* and *ces-4* single mutants presented a septum similar to wild type, while the double mutant *stk ces-4* disclosed a defect in septum morphology (Figure 4A). The double mutant was characterized by a partial failure of septum fusion in the central portion of the septum in conjunction with the medium portion of the ovary (Figure 4A). The quadruple mutant combinations *bee1 bee2 bee3 stk* and *bee1 bee2 bee3 ces-4* did not display defects in septum morphology when compared to wild type (Figure 4A). However, the damage of the septum appears stronger in the quadruple mutant *bee1 bee3 stk ces-4* and in the quintuple mutant *bee1 bee2 bee3 stk ces-4* where a hole in the central portion of the septum was seen in conjunction with the medium region of the ovary (Figure 4A).

The septum morphological analysis at stage 17-B suggests a transmitting tract defect caused by a failure of the carpel fusion which happens from stage 9 to 12 (Roeder and Yanofsky, 2006). At these stages, fusion occurs within the two crests of the CMM leading to septum and transmitting tract formation (Bowman et al., 1999; Roeder and Yanofsky, 2006; Reyes-Olalde et al., 2013).

To confirm that the defects found in the septum and transmitting tract formation, described for *stk ces-4*, *bee1 bee3 stk ces-4*, and *bee1 bee2 bee3 stk ces-4* caused a defective growth of the pollen tubes through the ovary transmitting tract, pistils were hand pollinated with wild type pollen and an aniline blue staining was performed after 36 h (Figure 4B). In the wild-type-hand-pollinated control pistils all the pollen tubes reached the bottom part of the ovary. However, pollen tube growth was compromised in the three mutant combinations. In the *stk ces-4* double mutant the pollen tubes did not grow further than the middle region of ovary length (Figure 4B). A stronger phenotype was observed in *bee1 bee3 stk ces-4* quadruple and in the *bee1 bee2 bee3 stk ces-4* quintuple mutants, where the pollen tubes stopped growing at the upper part of the ovary (Figure 4B).

The differences in septum morphology and in pollen tube growth in the double mutant *stk ces-4*, in the quadruple mutant *bee1 bee3 stk ces-4* and in the quintuple mutant *bee1 bee2 bee3 stk ces-4* clearly indicate that the fusion of the carpels, that permits the correct development of the septum and consequently of the transmitting tract, was partially disturbed in these mutants and the cause that the ovules at the lower part of the siliques remained unfertilized.

### SEEDSTICK and CESTA Interact in Yeast and *Planta*

**FIGURE 5:**
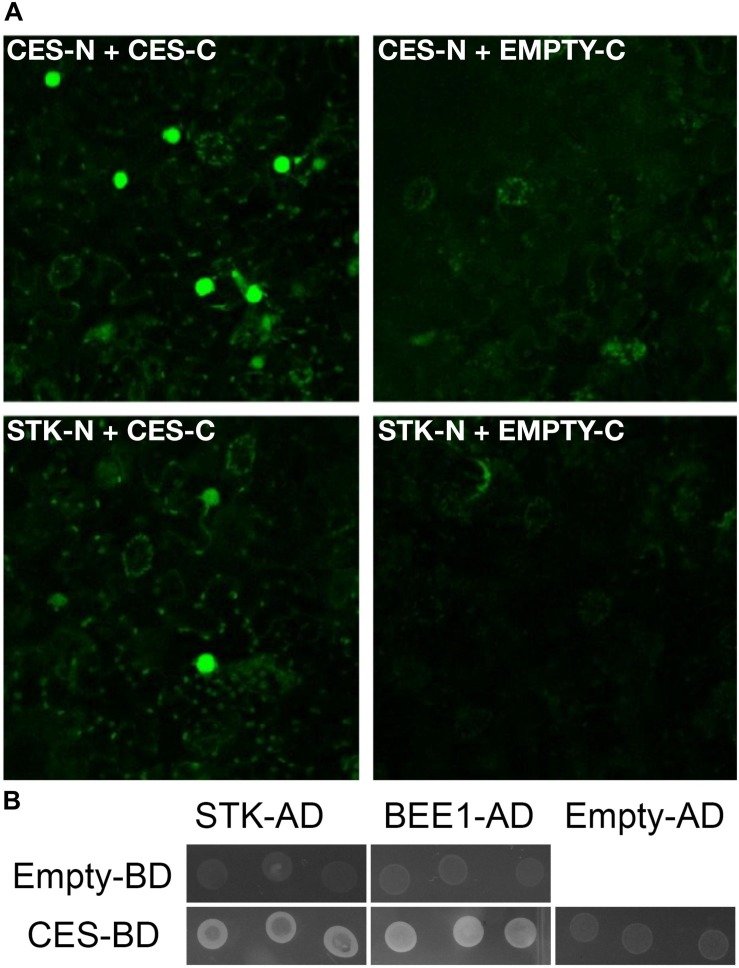
STK, CES and BEE1 interaction assays in yeast and *in planta*. **(A)** BiFC assays showing the interaction of STK with CES. STK-N or CES-N: STK or CES fusion with the N-terminal part of the split YFP; STK-C or CES-C: STK or CES fusion with the C-terminal part of the split YFP. CES-N CES-C interaction was used as positive control. STK-N EMPTY-C and CES-N EMPTY-C were used as negative control. **(B)** Yeast two-hybrid assays testing interactions between STK-AD, BEE1-AD, and CES-BD; interactions were considered positive when growth on -W-L-H +15 mM of 3-AT selective media was observed. The experiments were performed at 22°C. The CES-BEE1 interaction was chosen as a positive control (Poppenberger et al., 2011), the growth of transformants carrying empty AD or BD vectors were used as negative controls of the experiments.

The experiments described above showed a genetic interaction between *STK* and *CES.* To investigate if there is also a physical interaction between these proteins, Bimolecular Fluorescence Complementation (BiFC) was performed. As a positive control, cells were co-transformed with CES (YC-CES) and (YN-CES), as CES was shown to homodimerize in yeast (Poppenberger et al., 2011; Figure 5A). Reconstitution of YFP fluorescence in the nuclei of co-transformed cells confirmed that CES is able to interact with STK *in vivo* (Figure 5A), supporting our idea that CES and STK form a complex that controls transmitting tract formation. The interaction between STK and CES was also confirmed by Y2H experiments (Figure 5B). As positive control in these experiments we used the previously published interaction between BEE1 and CES (Poppenberger et al., 2011; Figure 5B). Furthermore, we observed that STK and BEE1 were not able to interact. The complete list of vector combinations, interactions and growth media used in this Y2H assay are described in Supplementary Table S3.

### Transcriptome Analysis of the *bee1 bee3 stk ces-4* Quadruple Mutant Reveals Target Genes Involved in Transmitting Tract Development

To obtain a snapshot of the regulatory network controlled by STK, CES, and BEE proteins, in the development of the septum/transmitting tract, transcriptional profiles of the quadruple mutant *bee1 bee3 stk ces-4*, which displayed the most severe phenotype, were studied by RNA sequencing. RNA was extracted from the quadruple mutant and wild type inflorescences until stage 12 of pistil development, before fertilization since the transmitting tract is fully developed at this stage (Bowman et al., 1999; Roeder and Yanofsky, 2006; Reyes-Olalde et al., 2013).

For the identification of significantly differentially expressed genes, a FDR cut-off value of 0.05 was applied and a total of 202 differentially expressed genes were identified. A total of 31 genes were upregulated in the mutant (Supplementary Table S5) while 171 genes were downregulated (Supplementary Table S6). Furthermore, functional enrichment analyses revealed that gene ontology categories such as glycosinolate, glycoside and carbohydrate biosynthetic and metabolic processes were enriched for the upregulated genes (Supplementary Table S7), while the downregulated genes were involved in the cell death, cell wall, plant-type cell wall biosynthesis components and response to hormone stimulus (Supplementary Table S8).

**FIGURE 6:**
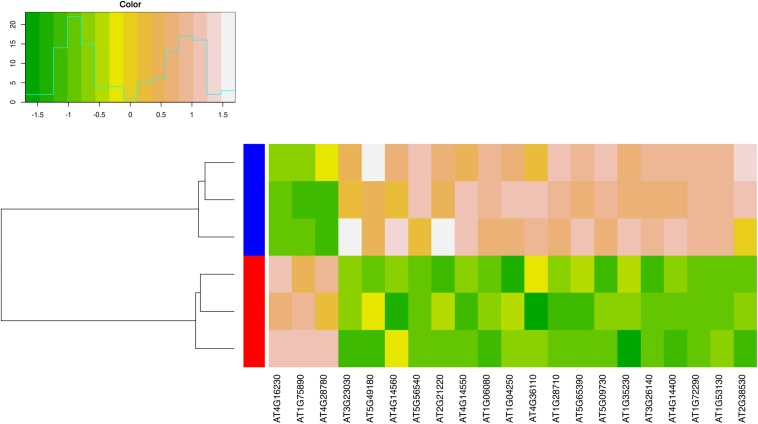
Heatmap of differentially expressed genes described in Table 1. Expression levels are shown for each of the 6 independent biological replicates analyzed in this study. Genes are represented in the columns and the biological replicates in the rows. To facilitate comparison, the expression values were standardized with respect to the average expression of each gene. Dark green indicates low expression, light brown indicates high expression. In the columns, red is used to mark biological replicates for the quadruple mutant, blue is used to indicate biological replicates for wild type plants.

Remarkably, among the downregulated genes, three genes were previously described as direct targets of STK (Herrera-Ubaldo et al., 2019): *AT3G26140*, *AT1G28710*, and *AT1G06080*. The *AT3G26140* gene which encodes a glycosyl hydrolase (GH5_11) is expressed in CMM during pistil development (Aspeborg et al., 2012; Herrera-Ubaldo et al., 2019). The GH5 enzymes are all mannan endo-beta-1,4-mannosidases which are known to be involved in cell wall remodeling processes (Aspeborg et al., 2012; Lombard et al., 2014; Herrera-Ubaldo et al., 2019). *AT1G28710* encodes for a nucleotide diphospho-sugar transferase protein which is involving in the biosynthesis of polysaccharides, that are fundamental components of the ECM (Lennon et al., 1998; Tung et al., 2005; Herrera-Ubaldo and de Folter, 2018). *AT1G06080* [*DELTA 9 DESATURASE1* (*ADS1*)], encodes for a fatty acid desaturase which is involved in lipid biosynthesis, expressed in flowers (Fukuchi-Mizutani et al., 1998; Yao et al., 2003; Heilmann et al., 2004; Table 1, see also Figure 6 for heatmap description). Downregulation of these three genes was also confirmed by qRT-PCR (Supplementary Figure S3).

**TABLE 1:** RNAseq data of candidate genes regulated by STK-CES protein complex and involved in transmitting tract development.

TAIR ID	Gene name	log FC	FDR	Gene function
AT4G14400	*ACD6*	–4.0117	2.33E-05	Cell death
AT1G06080	*ADS1*	–1.0338	2.17E-05	Cell wall and ECM biosynthesis
AT1G35230	*AGP5*	–2.1455	0.0145	Cell death and ECM biosynthesis
AT5G65390	*AGP7*	–1.3013	0.0024	Cell death and ECM biosynthesis
AT5G56540	*AGP14*	–0.7840	0.0473	Cell death and ECM biosynthesis
AT1G72290	*ATWSCP*	–6.5142	1.42E-07	Cell death and pollen tube growth
AT5G09730	*BXL3*	–2.0805	1.71E-06	Cell wall
AT1G75890	GDSL family of serine esterases/lipases	0.8845	0.0038	Cell wall and lipid metabolic process
AT4G28780	GDSL family of serine esterases/lipases	0.6227	0.0393	Cell wall and lipid metabolic process
AT4G16230	GDSL family of serine esterases/lipases	1.0662	0.0127	Cell wall and lipid metabolic process
AT3G26140	Glycosyl hydrolase family 5	–2.7099	3.13E-06	Cell wall and ECM biosynthesis
AT1G53130	*GRI*	–7.9981	4.16E-09	Cell death
AT4G14560	*IAA1*	–0.7727	0.0387	AUX signaling
AT3G23030	*IAA2*	–0.6711	0.0393	AUX signaling
AT4G14550	*IAA14*	–0.9381	0.0265	AUX signaling
AT1G04250	*IAA17*	–1.2215	0.0037	AUX signaling
AT2G38530	*LTP2*	–0.8702	0.0257	Lipid transport, cell death, cuticle biosynthesis
AT1G28710	Nucleotide diphospho-sugar transferase protein	–1.2338	0.00008	Cell wall and ECM biosynthesis
AT5G49180	*PME58*	–0.6750	0.0459	Cell wall
AT4G36110	*SAUR9*	–1.2233	0.0447	Auxin response
AT2G21220	*SAUR12*	–0.7960	0.0239	Auxin response

*Column 1, Gene ID according to the TAIR10 annotation of the A. thaliana genome; Column 2, Gene name; Column 3, Log Fold Change of expression between bee1 bee3 stk ces-4 and wild type plants; Column 4, FDR: Benjamini Hochberg adjusted P-value for differential expression. A Significance cut-off of 0.05 is applied; Column 5, Brief description of the gene according to TAIR10 (if available). Total RNA was extracted from three biological replicates (1 gr, obtained from a pool of 5 plants for each replicate) from both wild-type and bee1 bee3 stk ces-4 mutant inflorescences.*

PCD is a key process in the transmitting tract for ovule fertilization (Crawford et al., 2007). Interestingly, several genes involved in the regulation of cell death were downregulated in the quadruple mutant (Table 1 and Figure 6). One of the most important is *GRIM REAPER* (*GRI*, *AT1G53130*) that encodes for a small protein with an extracellular localization that is able to positively regulate cell death (Wrzaczek et al., 2009, 2015; Table 1 and Figure 6). Moreover, we observe that *ACCELERATED CELL DEATH 6* (*ACD6*, *AT4G14400*) another positive regulator of cell death is also downregulated (Rate et al., 2007; Table 1 and Figure 6).

Additionally, we notice the downregulation of three genes encoding for the Arabinogalactan proteins (AGPs) *ARABINOGALACTAN PROTEIN 5* (*AGP5*, *AT1G35230*), *AGP7* (*AT5G65390*), and *AGP14* (*AT5G56540*). AGPs are structurally complex plasma membrane and cell wall proteoglycans that are implicated in diverse developmental processes, including plant sexual reproduction (Lennon et al., 1998; Coimbra et al., 2007, 2009). Importantly, these protein are able to regulate cell death in Arabidopsis cell culture (Gao and Showalter, 1999; Guan and Nothnagel, 2004). Intriguingly, we also detect a significant downregulation of *ATWSCP* (*AT1G72290*), which encodes for a Kunitz-type protease inhibitor, member of a small family of proteins, that are water-soluble chlorophyll proteins, and *LIPID TRANSFER PROTEIN 2* (*LTP2, AT2G38530*) (Table 1 and Figure 6). *ATWSCP* is expressed in the septum and transmitting tract during its development, and after fertilization the protein is located in the medial domain of the silique where is able to regulate cell death and pollen tube growth (Bektas et al., 2012; Boex-Fontvieille et al., 2015). The *LTP2* gene encodes for a lipid transfer protein necessary for the movement of hydrophobic wax components through the hydrophilic cell wall matrix to reach the cuticle (Kunst and Samuels, 2003). LTP2 mediates cell wall loosening *in vitro* (Nieuwland et al., 2005).

Numerous genes involved in cell wall biosynthesis processes are mis-regulated in the quadruple mutant, these include *BETA-XYLOSIDASE 3* (*BXL3*, *AT5G09730*), which encodes for an enzyme involved in arabinan and xylan catabolic process (Minic et al., 2006), and *PECTIN METHYLESTERASE 58* (*PME58*, *AT5G49180*), encoding for a pectin methylesterases that contributes to cell wall modification (Turbant et al., 2016; Table 1 and Figure 6); both genes were downregulated. Among the upregulated genes, there are three genes of the GDSL family of serine esterases/lipases, *AT1G75890*, *AT4G28780*, and *AT4G16230* (Table 1 and Figure 6). These genes are involved in numerous aspect of plant development, but in particular the acetylxylan esterase activity of these enzymes could contribute in the degradation of cell walls in association with xylanases, cellulases, and mannanases (Akoh et al., 2004).

Moreover, some genes involved in the auxin signaling pathway were downregulated. These include genes encoding for Aux/IAAs proteins, *INDOLE-3-ACETIC ACID INDUCIBLE 1* (*IAA1*) (*AT4G14560*), *IAA2* (*AT3G23030*), *IAA14* (*AT4G14550*), and *IAA17* (*AT1G04250*) (Table 1 and Figure 6). The Aux/IAA proteins are the early auxin response proteins and participate in auxin signaling through interacting, as repressors, with AUXIN RESPONSE FACTOR (ARF) proteins (Lavy and Estelle, 2016). In addition, the *SMALL AUXIN UPREGULATED RNA 9* (*SAUR9*) and *SAUR12* were also downregulated. The SAURs are small transcripts induced by auxin, that are localized in elongated tissues (McClure and Guilfoyle, 1987; Knauss et al., 2003). *SAUR9* and *SAUR12* are part of the *SAUR10*-clade (van Mourik et al., 2017).

Finally, we decided to explore the enriched binding sites from our RNAseq data of the quadruple mutant in order to find possible direct target genes of the complex formed by STK, CES, BEE1, and BEE3. The binding sites used by the MADS-domain and bHLH transcription factors have been characterized: MADS factors bind to CArG boxes (Egea-Cortines et al., 1999; de Folter and Angenent, 2006) while bHLHs recognize E-boxes, being G-boxes the most common form (Quail, 2000; Toledo-Ortiz et al., 2003), in order to regulate the activity of their target genes. On the other hand, W-box is the binding site used by the WRKY transcription factors (Ciolkowski et al., 2008).

Transcription factor binding sites enrichment analyses, performed by means of the Pscan tool (Zambelli et al., 2009) suggest a strong enrichment of G-box and G-box like elements in the upregulated genes (Supplementary Table S9), while a significant enrichment of W-box and MADS-box elements is observed in the downregulated genes (Supplementary Table S10). Notably, according to our analyses G-box elements are identified in 35% (*p* = 4E-05) of the upregulated genes, while W-box elements and MADS box elements are identified in 17 and 19 downregulated genes (*p*-values 9.3E-04 and 0.035), respectively. Unsurprisingly, considering the limited number of genes that are differentially expressed in our experimental settings, we observe that only one gene has both a G-box and a CArG-box element in its promoter (*AT3G49270*, upregulated), only one gene has both a W-box and a MADS-box element (*AT1G05450*, downregulated), and none of the differentially expressed genes have a W-box and a G-box.

## Discussion

### Genetic Redundancy Between MADS-Domain and bHLH Transcription Factors in Transmitting Tract Development

In Arabidopsis, the gynoecium is formed by the fusion of two carpels, which fuse vertically at their margins and give rise to the CMM. From this meristem the transmitting tract develops, an essential tissue to facilitate the growing pollen tubes to reach the ovules and fertilize them.

Recently, a role for STK in relation with NTT was shown during the development of transmitting tract (Herrera-Ubaldo et al., 2019). Here we reveal that STK may also act in the downstream pathway of NTT in a protein complex with CES. Our genetic analysis evidence the role and redundancies between the MADS-box genes *STK*, *SHP1*, and *SHP2*, the bHLH transcription factor gene *CES* and the closely related bHLH transcription factors *BEE1* and *BEE3* in the development of the transmitting tract in *Arabidopsis thaliana*. The *CES* gene was selected based on high correlation of its expression pattern with *STK* (Mendes et al., 2016) but also because this gene was shown to play a role in transmitting tract development (Crawford and Yanofsky, 2011; Poppenberger et al., 2011). *CES*, *BEE1*, and *BEE3* were shown to be expressed with distinct but overlapping patterns within the reproductive tract (Crawford and Yanofsky, 2011; Poppenberger et al., 2011). In the *ces* (*haf*) *bee1 bee3* triple mutant ECM biosynthesis and PCD fail to occur within this tissue (Crawford and Yanofsky, 2011).

The *stk* mutant did not present any type of reproductive defect (no statistical differences when the number of unfertilized ovules was compared with wild type), while the single mutant *ces-4* is characterized by a small but significative percentage of unfertilized ovules (Figure 2). However, the *stk ces-4* double mutant had 28.1% of unfertilized ovules, which were almost exclusively found at the basal part of the silique (Figure 2). This phenotype suggests, similar to the *ces bee1 bee3* triple mutant (Crawford and Yanofsky, 2011; Poppenberger et al., 2011), a defect in transmitting tract development.

Interestingly, we also observed a genetic interaction between *STK* and *BEE1* and *BEE3* in transmitting tract development since the *bee1 bee2 bee3 stk* quadruple mutant showed 9.21% unfertilized ovules at the lower part of the silique, while *stk* had only 6.2% and the triple mutant *bee1 bee2 bee3* displayed 1.8% of unfertilized ovules (Figure 3). In the light of the fact that this phenotype was weaker than that observed for *stk ces-4* (Figure 2), we suggest that CES plays a more predominant role in transmitting tract formation together with STK. To understand a possible role of *SHP1* and *SHP2* in the formation of the transmitting tract, the triple mutant *shp1 shp2 ces-4* was obtained. This triple mutant showed 16.2% of unfertilized ovules which is also a milder phenotype when compared to *stk ces-4* (Figure 2). This suggests that STK is important for transmitting tract development, although is not able to cover for 100% *SHP*s function since the *shp1 shp2 ces-4* triple mutant presents a considerable percentage of unfertilized ovules.

Recently, it has been reported that in *Arabidopsis thaliana* the correct accomplishment of ovule fertilization directly influences the final length of the fruit. In the *ntt* mutant, in which the seeds only develop in the upper part of the fruit (Crawford et al., 2007), the authors described by using a fruit live imaging platform (FLIP) that in the upper part of the *ntt* siliques (where fertilization happens) the cells composing the valves elongate properly like in the wild type control while in the bottom part (where no fertilization occurs) the cells do not elongate properly (Ripoll et al., 2019). The quadruple mutant *bee1 bee3 stk ces-4*, which, similarly, to the *ntt* mutant displayed the highest percentage of unfertilized ovules from the middle to the bottom part of the fruit (Crawford et al., 2007), was characterized by a clear decrease in fruit length as can be seen in Figure 3, confirming the importance of ovule fertilization for fruit size.

The different degree of unfertilized ovule phenotypes obtained in the different mutant combinations points to redundancy between MADS-box, bHLH, and the BEEs transcription factor encoding genes in a dosage-dependent manner. The unfertilized ovules phenotype was only observed when different mutant alleles were combined and was more pronounced in the quadruple and quintuple mutants, suggesting an additive effect. Such a dosage dependent effect was already described for other MADS-box genes, like *FLOWERING LOCUS C* (*FLC*) that acts, in a dosage-dependent manner, as a potent repressor of the floral transition (Bastow et al., 2004; Michaels and Amasino, 2007; Sheldon et al., 2007).

Y2H and BIFC experiments showed that STK is able to interact with CES, but not with BEE1 (Figure 5 and Supplementary Table S3). This result suggests that CES might bridge the interaction between MADS and BEE proteins to form a hypothetical multimeric complex that controls correct transmitting tract formation. Cooperative action of MADS-domain and bHLH transcription factors has been previously described in mice. Molkentin et al. (1995) showed that MEF2 factors, a family of MADS-domain proteins expressed in muscle cells and other cell types, acted as coregulators of myogenic bHLH proteins to activate muscle genes (Molkentin et al., 1995). Moreover, the authors show that these two types of transcription factors physically interact, and that either factor can interact with the other when one is bound to DNA (Molkentin et al., 1995). Interestingly, the authors present this as a general mechanism for the regulation of transcription in specific cell types (Molkentin et al., 1995). This might also be a mechanism for the combinatorial control of MADS-box (with STK as a main player) and bHLH (CES as principal component) transcription factors for the formation of the transmitting tract tissue in Arabidopsis. Studies to understand the nature of a multimeric complex formed by MADS-box and bHLH proteins would really be a very interesting field for future studies.

### STK and CES Play a Role in Carpels Fusion

When we discovered, by backcrossing, that the male part was not compromised in the *stk ces-4* and *shp1 shp2 ces-4* multiple mutants (Supplementary Figure S2), we decided to perform a detailed analysis of the septum morphology of the mutants displaying the most stronger phenotype related to the percentage of unfertilized ovules. The single mutants *stk* and *ces-4* possess a similar septum respect to the wild type (Figure 4). However, when *STK* was mutated together with *CES* the fruits showed a septum fusion defect in the lower middle part in concomitance with the medial region of the ovary. The defective septum was found in the double mutant *stk ces-4*, in the quadruple mutant *bee1 bee3 stk ces-4* and in the quintuple mutant *bee1 bee2 bee3 stk ces-4*. Notably, no defective carpel fusion was revealed for the quadruple mutants *bee1 bee2 bee3 stk* and *bee1 bee2 bee3 ces-4* (Figure 4) which have a significant percentage of unfertilized ovules (Figure 3), confirming the hypothesis that only when *STK* and *CES* are mutated simultaneously the fusion of the carpels was in part compromised. Gynoecium defects were previously described only in the *ntt stk* double mutant, where there is a failure of the fusion with a septum that has a hole (Herrera-Ubaldo et al., 2019). The organ fusion defects were not disclosed for the single mutants *stk* and *ntt* and for the triple mutant *ces bee1 bee3* (Crawford and Yanofsky, 2011; Herrera-Ubaldo et al., 2019). In the *ces bee1 bee3* and probably in our quadruple mutant *bee1 bee2 bee3 ces-4* the unfertilized ovule phenotype is due to defects, as previously described by Crawford and Yanofsky (2011), in ECM components synthases and PCD failure in transmitting tract. In conclusion, only when the dimer STK-CES is not formed, the carpels fusion is compromised.

These phenotypes could be explained by mis-regulation of the genes involved in cuticle formation. The cuticle is a lipidic alteration of the cell wall (Yeats and Rose, 2013). The alteration in cuticle biosynthesis, deposition and wax component transports could cause organ fusion defects in plants, but in particular in flowers and carpels (Nawrath, 2006; Sieber et al., 2007; Panikashvili et al., 2010). Intriguingly transcriptional profiles of the quadruple mutant suggest a strong downregulation of *LTP2* (Table 1 and Figure 6), a gene involved in lipid transport and/or wax transport to form the cuticle (Kunst and Samuels, 2003). Notably, the *LTP5* gene, expressed in the transmitting tract, which belongs to the same gene family as *LTP2*, and which did not show significantly altered expression in the quadruple mutant according to our analysis, has previously been shown to be involved in pollen tube guidance (Kunst and Samuels, 2003; Chae et al., 2009). We believe that also *LTP2* might be involved in the correct fertilization of the ovules influencing not just the pollen tube growth but also the correct fusion of the two carpels that permits to form the septum and transmitting tract. Moreover, in the *bee1 bee3 stk ces-4* quadruple mutant we detected the overexpression of three genes encoding for GDSL esterase/lipase involved in lipid catabolism (Table 1 and Figure 6). The overexpression of a GDSL named, *CUTICLE DESTRUCTING FACTOR 1* (*CDEF1*), causes abnormal organ fusion in leaves, stems and flowers (Takahashi et al., 2010). This gene codes for a protein which is considered a cutinase (Takahashi et al., 2010). It is possible that like for the *CDEF1* overexpression, the excess of cutinase in the quadruple mutant respect to wild type caused abnormal organ fusion.

### STK and CES Influence Programmed Cell Death in Transmitting Tract

Cell death and tissue degeneration are required for pollen tube growth in the transmitting tract of Arabidopsis (Crawford et al., 2007). The phenotype described for combined *ces* and *bee* mutants showed that they fail to form the ECM and to accomplish PCD within the transmitting tract (Crawford and Yanofsky, 2011). Interestingly, STK has already been shown to control PCD in transmitting tract with NTT (Herrera-Ubaldo et al., 2019) but also in the receptive synergid cell through regulation of its direct target genes, *VDD* and *VAL*, two Reproductive Meristem (REM) transcription factors (Matias-Hernandez et al., 2010; Mendes et al., 2016).

As we show in the Table 1, in the quadruple mutant *bee1 bee3 stk ces-4* several genes involved in cell death are downregulated. The *ATWSCP* gene encodes for a Kunitz-type protease inhibitor localized preferentially in the transmitting tract and septum, which is involved in ECM and PCD but also in the exit of pollen tubes from the septum to reach the ovules during the fertilization phase (Bektas et al., 2012; Boex-Fontvieille et al., 2015). Also, *ACD6* is downregulated (Table 1 and Figure 6), this gene encodes for a protein that promotes cell death in Arabidopsis (Rate et al., 2007). Furthermore, three Arabinogalactan genes (*AGPs*) resulted downregulated in the quadruple mutant (Table 1 and Figure 6). The AGPs have been associated with ECM composition of the transmitting tract in different plant species (Hoggart and Clarke, 1984; Webb and Williams, 1988; Gane et al., 1995) and they have been shown to play a role in the regulation of PCD during pollen tube growth (Coimbra et al., 2009).

### Does Auxin-Brassinosteroid Crosstalk Influence Transmitting Tract Development?

It was recently suggested that the auxin pathway plays an important role in the specification and formation of the tissues along the pistil axis (Larsson et al., 2013; Robert et al., 2015). However, the role of auxin signaling in transmitting tract development is still not fully understood. ARF6 and ARF8 regulate *CES* expression in the medial domain of the pistil during transmitting tract development (Crawford and Yanofsky, 2011). The double mutant *arf6 arf8* displayed reduced alcian blue staining, that detects acidic polysaccharides, the major components of the ECM (Crawford and Yanofsky, 2011). According to our RNAseq data, four *Aux*/*IAA*s and two *SAUR*s genes were downregulated in the quadruple mutant in respect to wild type (Table 1 and Figure 6). Aux/IAA proteins and SAURs might be activated in presence of brassinosteroids since evidences of a crosstalk between auxin and brassinosteroid have already been described (Nakamura et al., 2006). Moreover, *SAUR9* expression is induced by a combination of auxin and brassinosteroids (van Mourik et al., 2017). Probably a STK-CES-BEE1-BEE3 complex is able to connect the auxin-brassinosteroid crosstalk in transmitting tract tissue, however, the precise role is of this crosstalk in the regulation of transmitting tract development remains unclear.

Finally, from the analysis of the enriched binding sites an interestingly candidate was found to be a possible direct target of the complex composed by the MADS and bHLH transcription factors described in this work. The *AT3G49270* gene (upregulated, see Supplementary Table S5) has both CArG and G boxes binding sites. It encodes for an extensin-like protein that has been suggested to have a possible role in postpollination responses such as cell wall protein modification or degradation associated with pistil receptivity (Boavida et al., 2011).

**FIGURE 7:**
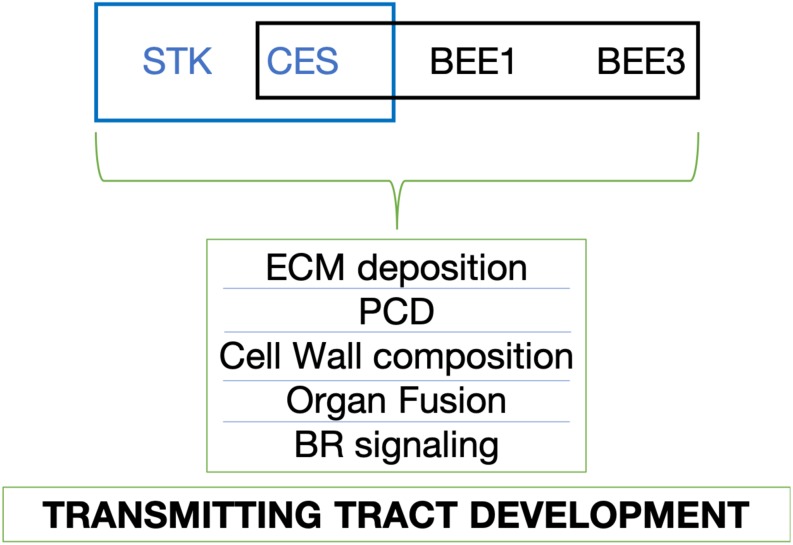
Proposed model of transmitting tract development controlled by STK, CES, BEE1, and BEE3. The blue box symbolizes the interaction (STK-CES) that we found in this research project; The black box represents the interaction (CES-BEE1-BEE3) that has been previously described (Crawford and Yanofsky, 2011; Poppenberger et al., 2011). ECM, extracellular matrix; PCD, programmed cell death; BR, brassinosteroid.

In summary, the data presented here suggest that a MADS-box-bHLH protein complex mastered by the physical interaction of STK-CES, plays a role in the regulation of genes involved in the PCD, ECM and cell wall biosynthesis pathways and by that controlling correct carpel fusion and transmitting tract development to guarantee efficient ovule fertilization (Figure 7).

## Data Availability Statement

The datasets generated for this study can be found in the Gene Expression Omnibus (GEO) database under the accession number GSE135559 (https://www.ncbi.nlm.nih.gov/geo/query/acc.cgi?acc=GSE135559).

## Author Contributions

MD and IR-V designed, performed, analyzed experiments, and wrote the manuscript. EZ, PB, AG, FC, EC, and VG performed experiments. MC performed bio-informatics analyses. MM performed aniline blue staining, analyzed all the results and, contributed writing the manuscript. LC and MK designed experiments, research strategies, and contributed to the writing of the manuscript. All the authors contributed to manuscript revision, read and approved the submitted version.

## Conflict of Interest

The authors declare that the research was conducted in the absence of any commercial or financial relationships that could be construed as a potential conflict of interest.

## Footnotes

1 http://www.ibmcp.upv.es/FerrandoLabVectors.php

2 http://www.bio-rad.com
