# Supplementary material for: MADS-Box and bHLH Transcription Factors Coordinate Transmitting Tract Development in Arabidopsis thaliana
Source: Front Plant Sci. 2020 May 6;11:526. doi: 10.3389/fpls.2020.00526 (PMC7219087; doi:10.3389/fpls.2020.00526)
Supplement: Supplementary file 1 [file Data_Sheet_1.PDF]

Supplementary Figures and Tables

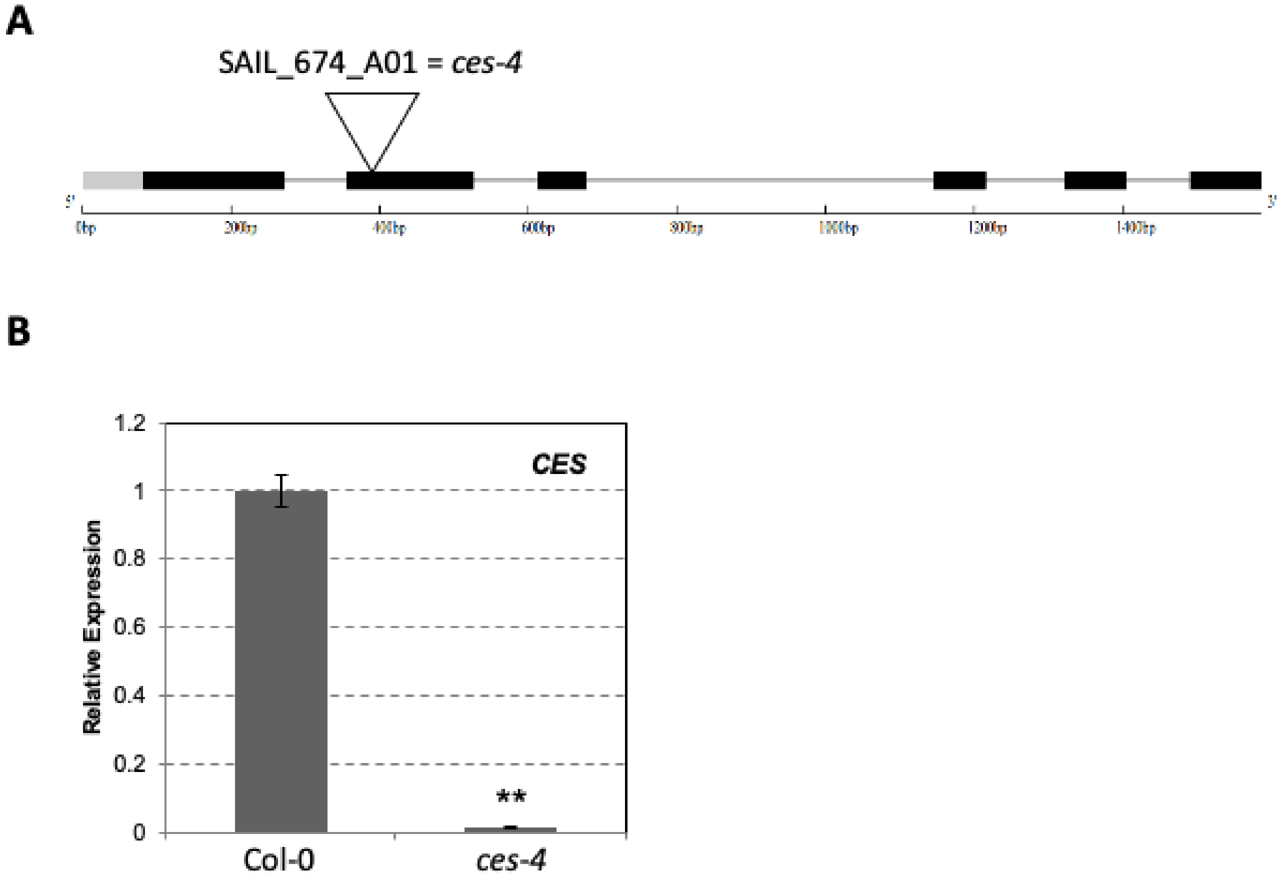

Supplementary Figure 1: (A) Schematic representation of *CES* (At1g25330) genomic sequence and the T-DNA insertion corresponding to *ces-4*. The exons (black boxes) and the 5’ UTR region (grey box) are shown. (B) Transcript abundance of *CES* in wt (Col-0) and *ces-4* plants, normalized to *ACTIN8*. Values are the mean ± s.e. of three independent qRT-PCR biological replicates. Asterisks indicate significant differences (**p<0.01) between the mutant and wild-type genotypes analysed through Student’s *t*-test.

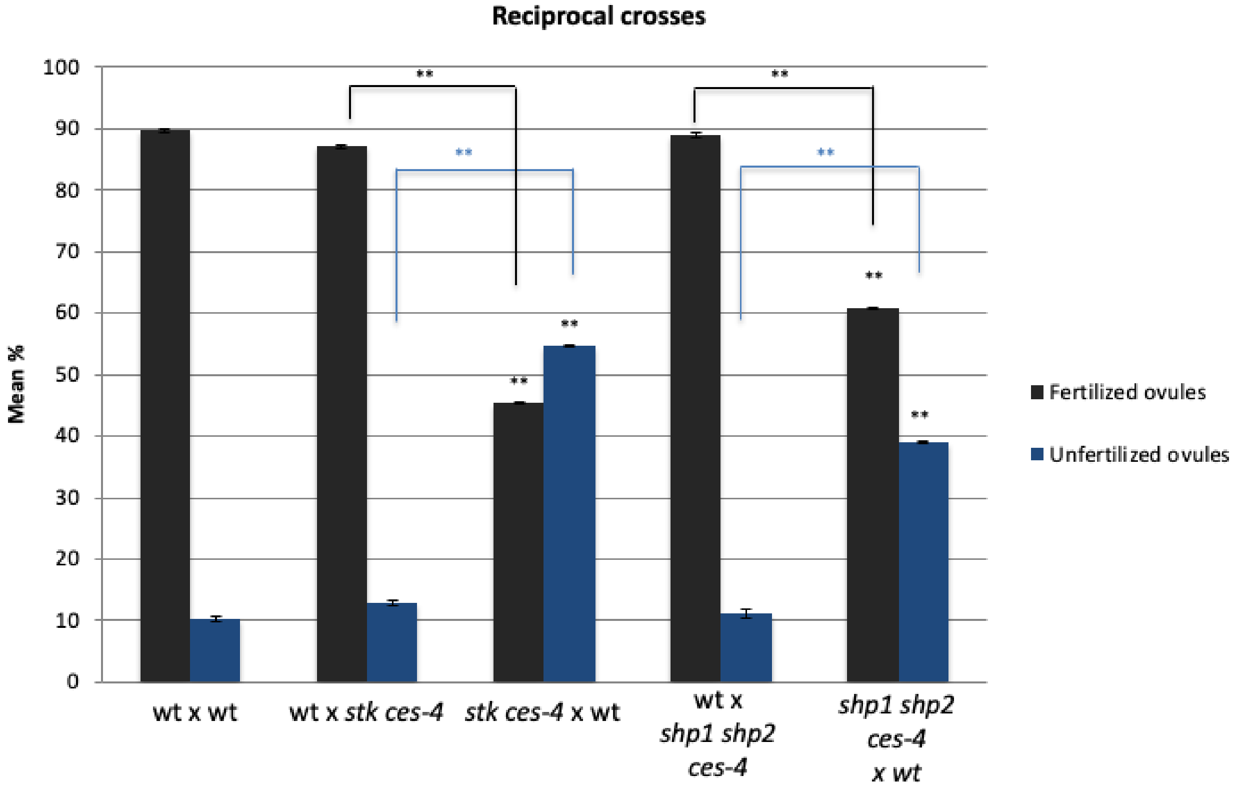

Supplementary Figure 2: Analysis of reciprocal crosses between wild type and different mutant combinations. On the x-axis: female x male genotypes. Statistical analysis was performed using Anova followed by Tukey HSD tests (** p<0.01).

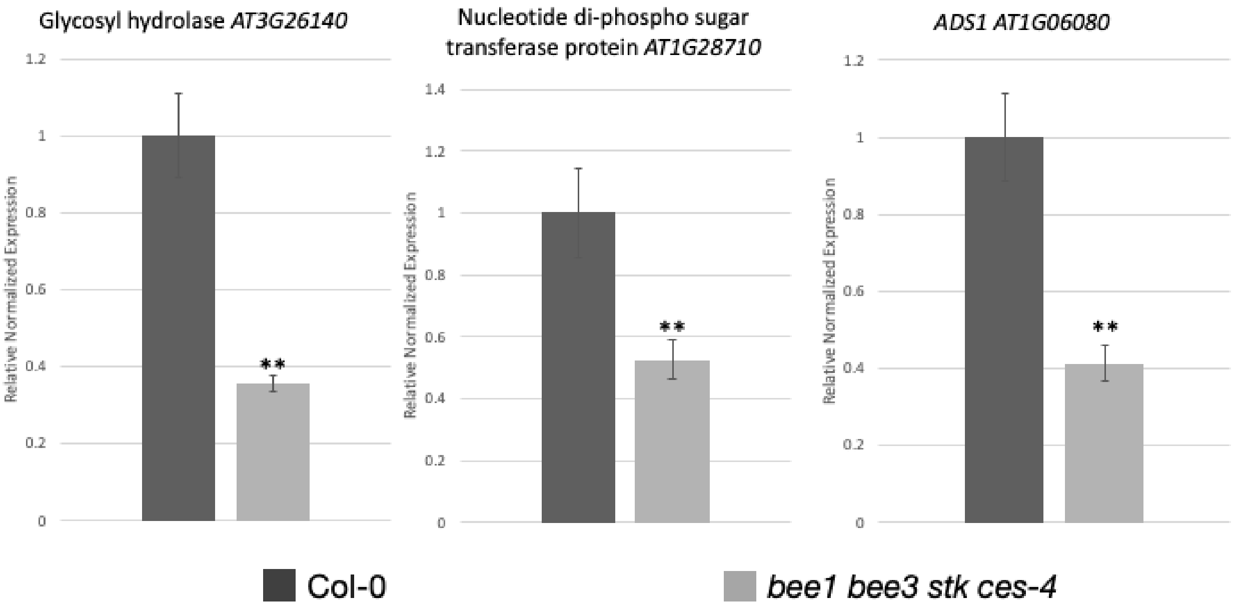

Supplementary Figure 3: qRT-PCR to validate RNA sequencing data. The genes selected to validate are: *AT3G26140*; *AT1G28710* and *AT1G06080*. Asterisks indicate significant differences (**p<0.01) between the mutant and wild-type genotypes analysed through Student's *t*-test.

Genotype	Col-0	*stk*	*ces-4*	*shp1 shp2*	*stk ces-4*	*shp1 shp2 ces-4*
Average	1.0	6.2	11.6	7.0	28.1	16.2
Median	0	6.5	8.5	3.3	29.1	14.1
Max	5.4	13.5	47.4	25.6	45.0	31.7
Min	0	0	0	0	13.2	4.8

Supplementary Table 1: Unfertilized ovules data of the *stk and ces-4* single mutants*, the stk ces-4* double and the *shp1 shp2 ces-4* triple mutant.

Genotype	**Col-0**	***bee1***	***bee3***	***bee1 bee3***	***bee1 bee2 bee3***	***bee1 bee2 bee3 stk***	***bee1 bee2 bee3 ces-4***	***bee1 bee3 stk ces-4***	***bee1 bee2 bee3 stk ces-4***
Average	0.5	0.7	1.4	1.0	1.7	9.2	28.7	46.5	43.7
Median	0	0	0	0	1	9.2	30.2	46.3	43.3
Max	2.2	9.3	5.1	5.3	8.8	15.6	38.6	61.4	54.2
Min	0	0	0	0	0	2.5	15.3	38	32.6

Supplementary Table 2: Unfertilized ovules data of the *bee1 and bee3* single mutants*,* the *bee1 bee3* double mutant*,* the *bee1 bee2 bee3* triple mutant*,* the *bee1 bee2 bee3 stk*, *bee1 bee2 bee3 ces-4*, *bee1 bee3 stk ces-4* quadruple mutants and the *bee1 bee2 bee3 stk ces-4* quintuple mutant.

AD	BD	-H-L-W			-H-L-W 5mMtriAT			-H-L-W 10mMtriAT			-H-L-W 15mMtriAT			-L-W-A		
		Replicates			Replicates			Replicates			Replicates			Replicates		
		1	2	3	1	2	3	1	2	3	1	2	3	1	2	3
STK	empty	++	++	++	-	-	-	-	-	-	-	-	-	-	-	-
empty	STK	+	+	+	-	-	-	-	-	-	-	-	-	-	-	-
CESTA	empty	+	+	+	-	-	-	-	-	-	-	-	-	-	-	-
empty	CESTA	++	++	++	+	+	+	+/-	+/-	+/-	-	-	-	++	++	++
BEE1	empty	+	+	+	-	-	-	-	-	-	-	-	-	-	-	-
empty	BEE1	+	+	+	-	-	-	-	-	-	-	-	-	-	-	-
**STK**	**CESTA**	++	++	++	++	++	++	++	++	++	++	++	++	++	++	++
CESTA	STK	+	+	+	-	-	-	-	-	-	-	-	-	-	-	-
STK	BEE1	+	+	+	-	-	-	-	-	-	-	-	-	-	-	-
BEE1	STK	+	+	+	-	-	-	-	-	-	-	-	-	-	-	-
CESTA	BEE1	++	++	++	++	++	++	+	+	+	-	-	-	++	++	++
BEE1	CESTA	++	++	++	++	++	++	++	++	++	++	++	++	++	++	++

Supplementary Table 3: The yeast 2-hybrid assays have been tested on media lacking Histidine and supplemented with different concentration of 3-AT. For each interaction, three independent transformant colonies were tested. pGADT7 and pGBKT7 vectors were employed. ++ = strong growth/interaction; + = intermediate growth/interaction; +/- = weak growth/interaction; - = no growth/interaction.

Sample	Pairs of Reads	% mapped on reference transcriptome	% mapped on reference genome
**Col-0 (1st replicate)**	30170637	69.98%	83.41%
**Col-0 (2nd replicate)**	24797986	63.97%	81.23%
**Col-0 (3rd replicate)**	26560305	66.26%	82.53%
bee1 bee3 stk ces-4 **(1st replicate)**	31604396	67.13%	82.56%
bee1 bee3 stk ces-4 **(2nd replicate)**	25725335	67.69%	81.53%
bee1 bee3 stk ces-4 **(3rd replicate)**	28137608	68.29%	83.56%

Supplementary Table 4: Number of reads obtained from each Col-0 (wt) and *bee1 bee3 stk ces-4* quadruple mutant replicate; % mapped on reference transcriptome; % mapped on reference genome.

TAIR Gene id	Col-0	*bee1 bee3 stk ces-4*	logFC	PValue	FDR	Gene Symbol and brief description
AT5G26970	0.04	0.89	4.43	0.00	0.04	unknown protein
AT3G60170	0.11	0.93	3.07	0.00	0.03	copia-like retrotransposon family
AT5G65080	2.33	13.27	2.49	0.00	0.00	MADS AFFECTING FLOWERING 5 (MAF5)
AT3G26460	0.46	2.23	2.27	0.00	0.00	Polyketide cyclase/dehydrase and lipid transport superfamily protein
AT2G05914	0.72	2.99	2.04	0.00	0.00	unknown protein
AT3G19710	0.62	2.25	1.84	0.00	0.04	branched-chain aminotransferase4 (BCAT4)
AT1G62560	2.26	6.79	1.57	0.00	0.00	flavin-monooxygenase glucosinolate S-oxygenase 3 (FMO GS-OX3)
AT5G35935	8.12	23.24	1.52	0.00	0.05	copia-like retrotransposon family
AT5G43500	19.87	49.74	1.33	0.00	0.00	actin-related protein 9 (ARP9)
AT4G12030	1.62	3.94	1.28	0.00	0.04	bile acid transporter 5 (BAT5)
AT5G14200	2.99	7.23	1.26	0.00	0.01	isopropylmalate dehydrogenase 1
AT4G13770	10.30	24.85	1.26	0.00	0.03	cytochrome P450
AT4G08115	1.74	4.16	1.26	0.00	0.04	gypsy-like retrotransposon family
AT5G52390	23.30	52.92	1.18	0.00	0.01	PAR1 protein
AT5G23010	2.72	6.15	1.16	0.00	0.04	methylthioalkylmalate synthase 1 (MAM1)
AT3G49270	6.10	13.00	1.09	0.00	0.01	molecular_function unknown
AT4G16230	2.19	4.59	1.07	0.00	0.01	GDSL-like Lipase/Acylhydrolase superfamily protein
AT4G23600	60.31	123.75	1.03	0.00	0.03	CORONATINE INDUCED 1 (CORI3)
AT4G15210	276.54	544.47	0.98	0.00	0.01	ARABIDOPSIS THALIANA BETA-AMYLASE (ATBETA-AMY)
AT1G75890	5.91	10.96	0.88	0.00	0.00	GDSL-like Lipase/Acylhydrolase superfamily protein
AT1G62540	39.98	69.16	0.79	0.00	0.05	flavin-monooxygenase glucosinolate S-oxygenase 2 (FMO GS-OX2)
AT1G48750	186.47	321.36	0.78	0.00	0.01	Bifunctional inhibitor/lipid-transfer protein/seed storage 2S albumin superfamily protein
AT2G32430	55.42	92.39	0.74	0.00	0.04	Galactosyltransferase family protein
AT1G74930	66.60	107.40	0.69	0.00	0.04	ORA47
AT4G19430	76.20	123.02	0.69	0.00	0.02	unknown protein
AT1G60190	27.48	44.08	0.68	0.00	0.00	ARM repeat superfamily protein
AT4G28780	21.20	32.74	0.62	0.00	0.04	GDSL-like Lipase/Acylhydrolase superfamily protein
AT4G30650	69.15	106.47	0.62	0.00	0.02	Low temperature and salt responsive protein family
AT3G54600	13.57	20.37	0.58	0.00	0.03	Class I glutamine amidotransferase-like superfamily protein
AT3G54820	50.69	73.23	0.53	0.00	0.03	plasma membrane intrinsic protein 2
AT4G39940	18.44	26.45	0.52	0.00	0.04	APS-kinase 2 (AKN2)

Supplementary Table 5: RNAseq, genes upregulated in the quadruple mutant *bee1 bee3 stk ces-4* compared to wild type. Column 1: Gene id according to the TAIR10 annotation of the *A. thaliana* genome; Column 2: Average expression in the wild type background. Values are expressed in log Counts Per Million (CPM); Column 3: Average expression in the *bee1 bee3 stk ces-4* mutant. Values are expressed in log Counts Per Million (CPM); Column 4: Log Fold change of expression between mutant and wild type plants; Column 5: p-value for differential expression; Column 6: Benjamini

Hochberg adjusted P-value for differential expression. A Significance cut-off of 0.05 is applied; Column 7: Brief description of the gene according to TAIR10 (if available).

TAIR Gene id	Col-0	*bee1 bee3 stk ces-4*	logFC	PValue	FDR	Gene Symbol and brief description
AT2G32890	4.62	0.00	-9.43	0.00	0.00	RALF-like 17 (RALFL17)
AT4G13266	3.50	0.00	-9.04	0.00	0.00	unknown protein
AT4G18540	1.84	0.00	-8.11	0.00	0.00	unknown protein
AT1G53130	23.38	0.09	-8.00	0.00	0.00	GRIM REAPER (GRI)
AT3G12000	55.84	0.21	-7.95	0.00	0.00	S-locus related protein SLR1, putative (S1)
AT5G19880	7.97	0.03	-7.62	0.00	0.00	Peroxidase superfamily protein
AT1G53480	40.96	0.24	-7.42	0.00	0.00	MTO 1 RESPONDING DOWN 1 (MRD1)
AT1G23720	0.67	0.00	-6.68	0.00	0.01	Proline-rich extensin-like family protein
AT1G72290	74.93	0.82	-6.51	0.00	0.00	Kunitz family trypsin and protease inhibitor protein
AT3G03670	4.17	0.07	-5.81	0.00	0.00	Peroxidase superfamily protein
AT2G41800	7.43	0.16	-5.40	0.00	0.00	Protein of unknown function, DUF642
AT5G20330	11.39	0.34	-5.05	0.00	0.00	beta-1,3-glucanase 4 (BETAG4)
AT2G15080	0.71	0.02	-4.87	0.00	0.03	receptor like protein 19 (RLP19)
AT1G73830	4.32	0.15	-4.77	0.00	0.00	BR enhanced expression 3 (BEE3)
AT3G14520	8.69	0.34	-4.66	0.00	0.00	Terpenoid cyclases/Protein prenyltransferases superfamily protein
AT2G43795	0.96	0.04	-4.57	0.00	0.00	unknown protein
AT4G11290	17.90	0.83	-4.44	0.00	0.00	Peroxidase superfamily protein
AT2G01422	35.28	1.66	-4.39	0.00	0.00	unknown protein
AT1G25330	36.95	1.79	-4.36	0.00	0.00	basic helix-loop-helix (bHLH) DNA-binding superfamily protein
AT1G61560	10.74	0.57	-4.24	0.00	0.00	MILDEW RESISTANCE LOCUS O 6 (MLO6)
AT2G43610	31.11	1.73	-4.18	0.00	0.00	Chitinase family protein
AT5G22980	16.89	0.90	-4.18	0.00	0.00	serine carboxypeptidase-like 47 (scpl47)
AT3G22250	1.01	0.05	-4.13	0.00	0.03	UDP-Glycosyltransferase superfamily protein
AT2G40680	2.44	0.13	-4.12	0.00	0.00	similar to unknown protein [Arabidopsis thaliana] (TAIR:AT5G52065.1)
AT2G46960	0.92	0.05	-4.01	0.00	0.00	cytochrome P450, family 709, subfamily B, polypeptide 1 (CYP709B1)
AT4G14400	3.11	0.18	-4.01	0.00	0.00	ACCELERATED CELL DEATH 6 (ACD6)
AT5G36110	3.08	0.19	-4.00	0.00	0.00	cytochrome P450, family 716, subfamily A, polypeptide 1 (CYP716A1)
AT4G34250	5.98	0.39	-3.92	0.00	0.00	3-ketoacyl-CoA synthase 16 (KCS16)
AT4G00910	1.99	0.15	-3.65	0.00	0.00	Aluminium activated malate transporter family protein
AT3G61035	2.74	0.23	-3.50	0.00	0.00	Cytochrome P450 superfamily protein
AT4G26420	0.80	0.07	-3.45	0.00	0.01	GAMT1
AT1G36060	3.06	0.30	-3.31	0.00	0.00	Integrase-type DNA-binding superfamily protein
AT1G30795	29.33	3.04	-3.27	0.00	0.00	Glycine-rich protein family
AT3G02940	1.69	0.16	-3.26	0.00	0.00	myb domain protein 107 (MYB107)
AT3G22231	2.53	0.27	-3.23	0.00	0.00	PATHOGEN AND CIRCADIAN CONTROLLED 1 (PCC1)
AT3G59850	3.91	0.44	-3.13	0.00	0.00	Pectin lyase-like superfamily protein

AT5G57550	1.83	0.20	-3.12	0.00	0.01	xyloglucan endotransglucosylase/hydrolase 25 (XTH25)
AT5G58782	7.82	0.92	-3.07	0.00	0.00	Undecaprenyl pyrophosphate synthetase family protein
AT5G20260	2.16	0.25	-3.07	0.00	0.00	Exostosin family protein
AT1G67105	6.83	0.82	-3.06	0.00	0.00	unknown protein
AT5G41040	3.66	0.48	-2.91	0.00	0.01	HXXXD-type acyl-transferase family protein
AT5G24110	1.58	0.22	-2.89	0.00	0.04	WRKY DNA-binding protein 30 (WRKY30)
AT4G33600	3.54	0.49	-2.84	0.00	0.00	unknown protein
AT3G04210	15.05	2.14	-2.78	0.00	0.02	Disease resistance protein (TIR-NBS class)
AT4G33610	0.70	0.10	-2.72	0.00	0.02	glycine-rich protein
AT3G26140	31.18	4.77	-2.71	0.00	0.00	Cellulase (glycosyl hydrolase family 5) protein
AT3G50470	1.59	0.23	-2.68	0.00	0.04	homolog of RPW8 3 (HR3)
AT3G59030	1.34	0.22	-2.58	0.00	0.00	TRANSPARENT TESTA 12 (TT12)
AT2G17000	5.18	0.89	-2.51	0.00	0.00	Mechanosensitive ion channel family protein
AT5G35390	7.94	1.40	-2.49	0.00	0.00	Leucine-rich repeat protein kinase family protein
AT4G23370	0.66	0.12	-2.40	0.00	0.04	unknown protein
AT3G26210	3.50	0.70	-2.34	0.00	0.02	cytochrome P450, family 71, subfamily B, polypeptide 23 (CYP71B23)
AT4G09960	51.42	10.22	-2.33	0.00	0.00	SEEDSTICK (STK)
AT3G57260	1.31	0.24	-2.30	0.00	0.02	beta-1,3-glucanase 2 (BGL2)
AT3G30720	21.45	4.39	-2.27	0.00	0.00	QUA-QUINE STARCH (QQS)
AT5G44580	1.80	0.38	-2.24	0.00	0.00	unknown protein
AT2G02010	9.48	2.02	-2.22	0.00	0.00	glutamate decarboxylase 4 (GAD4)
AT1G53490	14.51	3.16	-2.20	0.00	0.00	RING/U-box superfamily protein
AT1G35230	1.37	0.29	-2.15	0.00	0.01	arabinogalactan protein 5 (AGP5)
AT5G09730	172.56	40.81	-2.08	0.00	0.00	beta-xylosidase 3 (BXL3)
AT3G24225	1.45	0.34	-2.07	0.00	0.01	CLAVATA3/ESR-RELATED 19 (CLE19)
AT2G15650	8.26	1.99	-2.06	0.00	0.00	copia-like retrotransposon family, has a 5.0e-227 P-value blast match to gb|AAO73527.1| gag-pol polyprotein (Glycine max) (SIRE1) (Ty1_Copia-family)
AT1G44970	124.88	30.16	-2.05	0.00	0.00	Peroxidase superfamily protein
AT1G08930	29.18	7.05	-2.03	0.00	0.03	EARLY RESPONSE TO DEHYDRATION 6 (ERD6)
AT3G62150	5.84	1.44	-1.98	0.00	0.03	P-glycoprotein 21 (PGP21)
AT1G18400	11.08	2.79	-1.98	0.00	0.00	BR enhanced expression 1 (BEE1)
AT4G23130	1.20	0.29	-1.96	0.00	0.04	cysteine-rich RLK (RECEPTOR-like protein kinase) 5 (CRK5)
AT4G11890	2.40	0.61	-1.96	0.00	0.03	Protein kinase superfamily protein
AT1G05450	9.36	2.46	-1.92	0.00	0.00	Bifunctional inhibitor/lipid-transfer protein/seed storage 2S albumin superfamily protein
AT1G72416	6.35	1.66	-1.91	0.00	0.04	Chaperone DnaJ-domain superfamily protein
AT4G19970	4.12	1.11	-1.89	0.00	0.00	CONTAINS InterPro DOMAIN/s: Nucleotide-diphospho-sugar transferase, predicted (InterPro:IPR005069)

AT3G50480	16.23	4.37	-1.87	0.00	0.01	homolog of RPW8 4 (HR4)
AT3G10560	4.80	1.32	-1.87	0.00	0.00	UNFERTILIZED EMBRYO SAC 9 (UNE9)
AT1G65790	2.33	0.62	-1.86	0.00	0.05	receptor kinase 1 (RK1)
AT2G47130	2.29	0.63	-1.85	0.00	0.01	NAD(P)-binding Rossmann-fold superfamily protein
AT2G46440	6.32	1.74	-1.83	0.00	0.01	cyclic nucleotide-gated channels (CNGC11)
AT4G37400	2.21	0.62	-1.81	0.00	0.01	cytochrome P450, family 81, subfamily F, polypeptide 3 (CYP81F3)
AT1G21130	6.91	2.02	-1.77	0.00	0.00	O-methyltransferase family protein
AT5G54060	1.44	0.43	-1.76	0.00	0.04	UDP-glucose:flavonoid 3-o-glucosyltransferase (UF3GT)
AT4G38550	35.15	10.36	-1.75	0.00	0.04	Arabidopsis phospholipase-like protein (PEARLI 4) family
AT5G38900	1.47	0.44	-1.72	0.00	0.01	Thioredoxin superfamily protein
AT5G11360	4.68	1.44	-1.68	0.00	0.02	Interleukin-1 receptor-associated kinase 4 protein
AT2G05540	57.76	18.17	-1.66	0.00	0.05	Glycine-rich protein family
AT4G21870	22.64	7.12	-1.66	0.00	0.00	HSP20-like chaperones superfamily protein
AT5G44130	8.02	2.52	-1.65	0.00	0.02	FASCICLIN-like arabinogalactan protein 13 precursor (FLA13)
AT1G76930	2.75	0.86	-1.65	0.00	0.02	extensin 4 (EXT4)
AT1G75040	5.83	1.84	-1.64	0.00	0.00	pathogenesis-related gene 5 (PR5)
AT2G33020	1.59	0.50	-1.63	0.00	0.02	receptor like protein 24 (RLP24)
AT4G36430	1.58	0.51	-1.62	0.00	0.01	Peroxidase superfamily protein
AT1G65490	10.48	3.45	-1.58	0.00	0.05	unknown protein
AT4G12890	16.84	5.78	-1.55	0.00	0.00	Gamma interferon responsive lysosomal thiol (GILT) reductase family protein
AT1G58889	6.30	2.19	-1.51	0.00	0.02	copia-like retrotransposon family, has a 0. P-value blast match to dbj|BAA78425.1| polyprotein (Arabidopsis thaliana) (AtRE1) (Ty1_Copia-element)
AT3G23120	8.86	3.12	-1.49	0.00	0.01	receptor like protein 38 (RLP38)
AT3G45940	8.15	2.90	-1.49	0.00	0.00	Glycosyl hydrolases family 31 protein
AT1G21250	32.47	11.81	-1.45	0.00	0.00	cell wall-associated kinase (WAK1)
AT2G45180	61.98	22.61	-1.45	0.00	0.02	Bifunctional inhibitor/lipid-transfer protein/seed storage 2S albumin superfamily protein
AT1G71690	3.96	1.47	-1.44	0.00	0.03	Protein of unknown function (DUF579)
AT1G51805	41.05	15.21	-1.42	0.00	0.04	Leucine-rich repeat protein kinase family protein
AT4G23170	5.00	1.91	-1.37	0.00	0.01	EP1
AT1G70680	9.24	3.60	-1.36	0.00	0.01	Caleosin-related family protein
AT5G44020	34.98	13.50	-1.36	0.00	0.04	HAD superfamily, subfamily IIIB acid phosphatase
AT5G50200	4.17	1.59	-1.36	0.00	0.04	WOUND-RESPONSIVE 3 (WR3)
AT2G25510	15.94	6.32	-1.32	0.00	0.02	unknown protein
AT2G40750	6.20	2.48	-1.31	0.00	0.01	WRKY DNA-binding protein 54 (WRKY54)
AT5G65390	4.95	2.00	-1.30	0.00	0.00	arabinogalactan protein 7 (AGP7)
AT3G56410	5.35	2.14	-1.30	0.00	0.04	Protein of unknown function (DUF3133)
AT4G22690	86.77	35.26	-1.30	0.00	0.04	cytochrome P450, family 706, subfamily A, polypeptide 1 (CYP706A1)
AT2G41090	33.06	13.45	-1.29	0.00	0.00	Calcium-binding EF-hand family protein

AT5G46590	4.50	1.83	-1.29	0.00	0.01	NAC domain containing protein 96 (NAC096)
AT2G18300	2.82	1.17	-1.25	0.00	0.04	basic helix-loop-helix (bHLH) DNA-binding superfamily protein
AT5G42230	18.10	7.62	-1.25	0.00	0.00	serine carboxypeptidase-like 41 (scpl41)
AT1G28710	51.85	22.04	-1.23	0.00	0.00	Nucleotide-diphospho-sugar transferase family protein
AT4G36110	5.39	2.29	-1.22	0.00	0.04	SAUR-like auxin-responsive protein family
AT1G04250	20.41	8.77	-1.22	0.00	0.00	AUXIN RESISTANT 3 (AXR3)
AT5G23510	4.67	1.98	-1.21	0.00	0.01	unknown protein
AT1G65190	5.45	2.41	-1.18	0.00	0.01	Protein kinase superfamily protein
AT1G32860	10.40	4.75	-1.13	0.00	0.00	Glycosyl hydrolase superfamily protein
AT1G18020	19.80	9.35	-1.08	0.00	0.04	FMN-linked oxidoreductases superfamily protein
AT5G38970	4.58	2.17	-1.06	0.00	0.04	brassinosteroid-6-oxidase 1 (BR6OX1)
AT1G06080	34.94	17.05	-1.03	0.00	0.00	delta 9 desaturase 1 (ADS1)
AT5G40780	14.17	6.95	-1.03	0.00	0.00	lysine histidine transporter 1
AT4G23010	28.88	14.27	-1.02	0.00	0.00	UDP-galactose transporter 2 (UTR2)
AT1G13110	4.48	2.22	-1.00	0.00	0.04	cytochrome P450, family 71 subfamily B, polypeptide 7 (CYP71B7)
AT4G36770	8.55	4.29	-1.00	0.00	0.01	UDP-Glycosyltransferase superfamily protein
AT1G35710	14.78	7.38	-1.00	0.00	0.01	Protein kinase family protein with leucine-rich repeat domain
AT2G19800	53.28	26.69	-1.00	0.00	0.04	myo-inositol oxygenase 2 (MIOX2)
AT3G51920	12.74	6.40	-0.98	0.00	0.03	calmodulin 9 (CAM9)
AT2G23200	15.59	7.89	-0.98	0.00	0.03	Protein kinase superfamily protein
AT5G51810	15.29	7.77	-0.98	0.00	0.01	gibberellin 20 oxidase 2 (GA20OX2)
AT4G14550	5.98	3.10	-0.94	0.00	0.03	indole-3-acetic acid inducible 14 (IAA14)
AT1G78780	11.42	5.96	-0.94	0.00	0.02	pathogenesis-related family protein
AT1G52290	11.61	6.08	-0.92	0.00	0.04	Protein kinase superfamily protein
AT5G24910	8.95	4.78	-0.90	0.00	0.02	cytochrome P450, family 714, subfamily A, polypeptide 1 (CYP714A1)
AT1G22740	7.71	4.13	-0.90	0.00	0.04	RAB GTPase homolog G3B (RABG3B)
AT5G60140	21.16	11.53	-0.88	0.00	0.01	AP2/B3-like transcriptional factor family protein
AT2G38530	92.36	50.55	-0.87	0.00	0.03	lipid transfer protein 2 (LTP2)
AT4G19460	9.41	5.21	-0.86	0.00	0.02	UDP-Glycosyltransferase superfamily protein
AT3G22240	19.93	11.12	-0.84	0.00	0.03	unknown protein
AT2G44080	13.66	7.76	-0.81	0.00	0.04	ARGOS-like (ARL)
AT1G06520	43.43	24.89	-0.80	0.00	0.05	glycerol-3-phosphate acyltransferase 1 (GPAT1)
AT2G21220	14.04	8.07	-0.80	0.00	0.02	SAUR-like auxin-responsive protein family
AT1G69730	13.68	7.93	-0.79	0.00	0.02	Wall-associated kinase family protein
AT5G56540	21.87	12.73	-0.78	0.00	0.05	arabinogalactan protein 14 (AGP14)
AT1G63260	15.91	9.23	-0.78	0.00	0.03	tetraspanin10 (TET10)
AT4G14560	17.88	10.46	-0.77	0.00	0.04	indole-3-acetic acid inducible (IAA1)
AT4G28490	32.10	18.90	-0.76	0.00	0.04	HAESA (HAE)
AT4G24040	29.43	17.31	-0.76	0.00	0.05	trehalase 1 (TRE1)
AT1G65480	29.65	17.52	-0.76	0.00	0.01	FLOWERING LOCUS T (FT)
AT3G12170	10.33	6.17	-0.75	0.00	0.04	Chaperone DnaJ-domain superfamily protein

AT2G13790	43.56	25.99	-0.74	0.00	0.03	somatic embryogenesis receptor-like kinase 4 (SERK4)
AT4G38860	8.32	4.98	-0.74	0.00	0.05	SAUR-like auxin-responsive protein family
AT4G30270	167.83	103.21	-0.70	0.00	0.02	xyloglucan endotransglucosylase/hydrolase 24 (XTH24)
AT5G14920	82.01	50.49	-0.70	0.00	0.05	Gibberellin-regulated family protein
AT1G03400	11.79	7.34	-0.68	0.00	0.03	2-oxoglutarate (2OG) and Fe(II)-dependent oxygenase superfamily protein
AT1G26540	23.65	14.74	-0.68	0.00	0.04	Agenet domain-containing protein
AT5G49180	35.05	21.97	-0.68	0.00	0.05	Plant invertase/pectin methylesterase inhibitor superfamily
AT1G34750	14.24	8.92	-0.67	0.00	0.02	Protein phosphatase 2C family protein
AT3G23030	49.78	31.20	-0.67	0.00	0.04	indole-3-acetic acid inducible 2 (IAA2)
AT5G21150	164.79	104.25	-0.66	0.00	0.00	ARGONAUTE 9 (AGO9)
AT5G61010	17.69	11.22	-0.66	0.00	0.03	exocyst subunit exo70 family protein E2 (EXO70E2)
AT5G59810	28.38	18.08	-0.65	0.00	0.05	SBT5.4
AT5G24530	31.32	20.06	-0.64	0.00	0.03	DOWNY MILDEW RESISTANT 6 (DMR6)
AT4G15450	43.04	27.75	-0.63	0.00	0.00	Senescence/dehydration-associated protein-related
AT1G58602	34.90	22.52	-0.63	0.00	0.05	LRR and NB-ARC domains-containing disease resistance protein
AT1G76520	22.31	14.62	-0.61	0.00	0.04	Auxin efflux carrier family protein
AT1G05490	55.61	36.68	-0.60	0.00	0.02	chromatin remodeling 31 (chr31)
AT3G51430	20.36	13.71	-0.57	0.00	0.04	YELLOW-LEAF-SPECIFIC GENE 2 (YLS2)
AT5G07580	35.63	24.01	-0.57	0.00	0.05	Integrase-type DNA-binding superfamily protein
AT5G18860	26.80	18.18	-0.56	0.00	0.02	inosine-uridine preferring nucleoside hydrolase family protein
AT3G47250	21.82	14.86	-0.55	0.00	0.03	Plant protein of unknown function (DUF247)
AT1G58180	31.22	21.48	-0.54	0.00	0.05	beta carbonic anhydrase 6 (BCA6)

Supplementary Table 6: RNAseq, genes downregulated in the quadruple mutant *bee1 bee3 stk ces-4* compared to wild type. Column 1: Gene id according to the TAIR10 annotation of the *A. thaliana* genome; Column 2: Average expression in the wild type background. Values are expressed in log Counts Per Million (CPM); Column 3: Average expression in the *bee1 bee3 stk ces-4* mutant. Values are expressed in log Counts Per Million (CPM); Column 4: Log Fold change of expression between mutant and wild type plants; Column 5: p-value for differential expression; Column 6: Benjamini Hochberg adjusted P-value for differential expression. A Significance cut-off of 0.05 is applied; Column 7: Brief description of the gene according to TAIR10 (if available).

GO_acc	term_type	Term	pvalue	FDR
GO:0019758	P	glycosinolate biosynthetic process	2.20E-14	8.10E-13
GO:0019761	P	glucosinolate biosynthetic process	2.20E-14	8.10E-13
GO:0016144	P	S-glycoside biosynthetic process	2.20E-14	8.10E-13
GO:0019757	P	glycosinolate metabolic process	3.20E-13	5.80E-12
GO:0019760	P	glucosinolate metabolic process	3.20E-13	5.80E-12
GO:0016143	P	S-glycoside metabolic process	3.20E-13	5.80E-12
GO:0016138	P	glycoside biosynthetic process	1.60E-12	2.50E-11
GO:0016137	P	glycoside metabolic process	9.80E-12	1.30E-10
GO:0044272	P	sulfur compound biosynthetic process	1.90E-11	2.30E-10
GO:0044262	P	cellular carbohydrate metabolic process	1.50E-10	1.60E-09
GO:0034637	P	cellular carbohydrate biosynthetic process	3.50E-10	3.50E-09
GO:0006790	P	sulfur metabolic process	1.50E-09	1.40E-08
GO:0016051	P	carbohydrate biosynthetic process	7.00E-09	6.00E-08
GO:0005975	P	carbohydrate metabolic process	7.50E-08	5.90E-07
GO:0019748	P	secondary metabolic process	3.10E-07	2.30E-06
GO:0050896	P	response to stimulus	0.00079	0.0054
GO:0044238	P	primary metabolic process	0.0012	0.008
GO:0009058	P	biosynthetic process	0.0015	0.0094
GO:0009719	P	response to endogenous stimulus	0.0029	0.016
GO:0008152	P	metabolic process	0.003	0.016
GO:0044249	P	cellular biosynthetic process	0.0038	0.02
GO:0010033	P	response to organic substance	0.0076	0.036
GO:0044237	P	cellular metabolic process	0.0075	0.036
GO:0003824	F	catalytic activity	0.0077	0.027
GO:0016740	F	transferase activity	0.0097	0.027
GO:0012505	C	endomembrane system	0.00018	0.0074
GO:0005623	C	cell	0.0021	0.029
GO:0044464	C	cell part	0.0021	0.029

Supplementary Table 7: Gene Ontology analysis of the genes upregulated in the quadruple mutant *bee1 bee3 stk ces-4* in comparison with wild type; Column 1: Gene Ontology (GO) term unique identifier; Column 2: Gene Ontology term functional category: P= biological process; F= molecular function; C= cellular component; Column 3: Gene ontology term official name; Column 4: p-value for the enrichment of the GO term in the gene set; Column 5: Benjamin Hochberg adjusted p-value, a significance cut-off of 0.05 is applied.

GO_acc	term_type	Term	pvalue	FDR
GO:0050896	P	response to stimulus	2.40E-08	9.20E-06
GO:0010033	P	response to organic substance	2.70E-07	5.10E-05
GO:0042221	P	response to chemical stimulus	2.30E-06	0.00029
GO:0019825	F	oxygen binding	1.80E-06	0.00036
GO:0020037	F	heme binding	4.90E-06	0.00048
GO:0051704	P	multi-organism process	6.20E-06	0.0006
GO:0051707	P	response to other organism	1.10E-05	0.00083
GO:0016798	F	hydrolase activity, acting on glycosyl bonds	1.70E-05	0.0011
GO:0009607	P	response to biotic stimulus	2.00E-05	0.0013
GO:0046906	F	tetrapyrrole binding	3.00E-05	0.0015
GO:0009719	P	response to endogenous stimulus	5.10E-05	0.0028
GO:0005506	F	iron ion binding	7.50E-05	0.003
GO:0003824	F	catalytic activity	0.00011	0.0036
GO:0009725	P	response to hormone stimulus	7.90E-05	0.0038
GO:0005488	F	binding	0.00016	0.0045
GO:0004553	F	hydrolase activity, hydrolyzing O-glycosyl compounds	0.00021	0.0047
GO:0016684	F	oxidoreductase activity, acting on peroxide as acceptor	0.00024	0.0047
GO:0004601	F	peroxidase activity	0.00024	0.0047
GO:0016209	F	antioxidant activity	0.0005	0.009
GO:0009751	P	response to salicylic acid stimulus	0.00024	0.01
GO:0016740	F	transferase activity	0.00066	0.011
GO:0030312	C	external encapsulating structure	0.00037	0.015
GO:0005618	C	cell wall	0.00035	0.015
GO:0009505	C	plant-type cell wall	0.00081	0.022
GO:0016301	F	kinase activity	0.0016	0.024
GO:0009055	F	electron carrier activity	0.0017	0.024
GO:0016491	F	oxidoreductase activity	0.0036	0.048
GO:0008219	P	cell death	0.0015	0.05
GO:0016265	P	death	0.0015	0.05

Supplementary Table 8: Gene Ontology analysis of the genes downregulated in the quadruple mutant *bee1 bee3 stk ces-4* in comparison with wild type. Column 1: Gene Ontology (GO) term unique identifier; Column 2: Gene Ontology term functional category: P= biological process; F= molecular function; C= cellular component; Column 3: Gene ontology term official name; Column 4: p-value for the enrichment of the GO term in the gene set; Column 5: Benjamin Hochberg adjusted p-value, a significance cut-off of 0.05 is applied.

TF_NAME	MATRIX_ID	Z_SCORE	P_VALUE	FDR	LINK
GT3a	MA1207.1	4.31353	7.868E-06	0.00385532	http://jaspar.genereg.net/matrix/MA1207.1
BEE2	MA0956.1	3.94487	3.9461E-05	0.01933604	http://jaspar.genereg.net/matrix/MA0956.1
BIM3	MA0966.1	3.90905	4.5779E-05	0.02243166	http://jaspar.genereg.net/matrix/MA0966.1
BHLH13	MA0958.1	3.84929	5.8544E-05	0.02868632	http://jaspar.genereg.net/matrix/MA0958.1
BIM2	MA0965.1	3.84595	5.9381E-05	0.02909664	http://jaspar.genereg.net/matrix/MA0965.1
PHYPADRAFT_143875	MA0988.1	3.78729	7.5274E-05	0.03688402	http://jaspar.genereg.net/matrix/MA0988.1
MYC4	MA0569.1	3.78721	7.5334E-05	0.03691346	http://jaspar.genereg.net/matrix/MA0569.1
PHYPADRAFT_72483	MA1011.1	3.7864	7.5626E-05	0.03705679	http://jaspar.genereg.net/matrix/MA1011.1
BHLH3	MA0957.1	3.70494	0.00010456	0.05123195	http://jaspar.genereg.net/matrix/MA0957.1
MYC3	MA0568.1	3.67038	0.00012004	0.05882009	http://jaspar.genereg.net/matrix/MA0568.1
bHLH31	MA1359.1	3.62362	0.00014396	0.07054187	http://jaspar.genereg.net/matrix/MA1359.1
MYC2	MA0566.1	3.61622	0.00014814	0.07258713	http://jaspar.genereg.net/matrix/MA0566.1
PHYPADRAFT_48267	MA1021.1	3.60101	0.00015696	0.07691089	http://jaspar.genereg.net/matrix/MA1021.1
UNE10	MA1074.1	3.5232	0.00021102	0.10340127	http://jaspar.genereg.net/matrix/MA1074.1
BIM1	MA0964.1	3.49284	0.00023673	0.11599623	http://jaspar.genereg.net/matrix/MA0964.1
ABF2	MA0941.1	3.44873	0.00027939	0.13689914	http://jaspar.genereg.net/matrix/MA0941.1
BHLH34	MA0962.1	3.33486	0.00042332	0.20742435	http://jaspar.genereg.net/matrix/MA0962.1
HBI1	MA1025.1	3.2935	0.00049091	0.24054345	http://jaspar.genereg.net/matrix/MA1025.1

SPT	MA1061.1	3.19183	0.0007012	0.34358849	http://jaspar.genereg.net/matrix/MA1061.1
AT4G18890	MA1333.1	3.09142	0.0009897	0.48495153	http://jaspar.genereg.net/matrix/MA1333.1
ABF3	MA0930.1	2.9817	0.00142495	0.6982255	http://jaspar.genereg.net/matrix/MA0930.1
BZR2	MA0549.1	2.96177	0.00151731	0.7434819	http://jaspar.genereg.net/matrix/MA0549.1
OJ1058_F05.8	MA1033.1	2.90869	0.00180171	0.8828379	http://jaspar.genereg.net/matrix/MA1033.1
PIF4	MA0561.1	2.89397	0.00188994	0.9260706	http://jaspar.genereg.net/matrix/MA0561.1
PIF3	MA0560.1	2.88917	0.00191878	0.9402022	http://jaspar.genereg.net/matrix/MA0560.1
AT1G78700	MA1331.1	2.86715	0.00206038	1	http://jaspar.genereg.net/matrix/MA1331.1
FUS3	MA0565.1	2.81707	0.00240833	1	http://jaspar.genereg.net/matrix/MA0565.1
AT4G36780	MA1332.1	2.81029	0.00246262	1	http://jaspar.genereg.net/matrix/MA1332.1
bHLH77	MA1362.1	2.75902	0.00287329	1	http://jaspar.genereg.net/matrix/MA1362.1
TSAR2	MA1412.1	2.73758	0.00307984	1	http://jaspar.genereg.net/matrix/MA1412.1
AIB	MA0959.1	2.70807	0.00336402	1	http://jaspar.genereg.net/matrix/MA0959.1
BZR1	MA0550.2	2.66734	0.00380463	1	http://jaspar.genereg.net/matrix/MA0550.2
ABI3	MA0564.1	2.57392	0.00500092	1	http://jaspar.genereg.net/matrix/MA0564.1
BHLH78	MA0963.1	2.47784	0.00657679	1	http://jaspar.genereg.net/matrix/MA0963.1
bHLH74	MA1360.1	2.45921	0.00692884	1	http://jaspar.genereg.net/matrix/MA1360.1
ABI5	MA0931.1	2.44837	0.00713783	1	http://jaspar.genereg.net/matrix/MA0931.1
MYB4	MA1039.1	2.41678	0.00778974	1	http://jaspar.genereg.net/matrix/MA1039.1
TSAR1	MA1411.1	2.40745	0.00799477	1	http://jaspar.genereg.net/matrix/MA1411.1

HY5	MA0551.1	2.40742	0.00799618	1	http://jaspar.genereg.net/matrix/MA0551.1
FHY3	MA0557.1	2.36779	0.00891595	1	http://jaspar.genereg.net/matrix/MA0557.1
BHLH104	MA0960.1	2.36143	0.00905036	1	http://jaspar.genereg.net/matrix/MA0960.1
ERF6	MA1006.1	2.3544	0.00922637	1	http://jaspar.genereg.net/matrix/MA1006.1
At4g01280	MA1190.1	2.32705	0.00995034	1	http://jaspar.genereg.net/matrix/MA1190.1
ERF018	MA1048.1	2.31497	0.010278	1	http://jaspar.genereg.net/matrix/MA1048.1
PIF5	MA0562.1	2.29135	0.0109205	1	http://jaspar.genereg.net/matrix/MA0562.1
NAC058	MA0938.1	2.27578	0.0113717	1	http://jaspar.genereg.net/matrix/MA0938.1
PIF1	MA0552.1	2.27181	0.0114889	1	http://jaspar.genereg.net/matrix/MA0552.1
At5g52660	MA1183.1	2.2614	0.0118306	1	http://jaspar.genereg.net/matrix/MA1183.1
EmBP-1	MA0128.1	2.25669	0.0119533	1	http://jaspar.genereg.net/matrix/MA0128.1
LEC2	MA0581.1	2.18485	0.0144018	1	http://jaspar.genereg.net/matrix/MA0581.1
PIF7	MA1364.1	2.14941	0.0157458	1	http://jaspar.genereg.net/matrix/MA1364.1
At3g09600	MA1182.1	2.1404	0.0161119	1	http://jaspar.genereg.net/matrix/MA1182.1
SPL3	MA0577.1	2.11325	0.0172358	1	http://jaspar.genereg.net/matrix/MA0577.1
ARR11	MA0946.1	2.04396	0.0204421	1	http://jaspar.genereg.net/matrix/MA0946.1
FAR1	MA1382.1	1.9996	0.0227076	1	http://jaspar.genereg.net/matrix/MA1382.1
At2g38090	MA1193.1	1.99479	0.0229634	1	http://jaspar.genereg.net/matrix/MA1193.1
LCL1	MA1187.1	1.99427	0.0230037	1	http://jaspar.genereg.net/matrix/MA1187.1
LHY1	MA1185.1	1.97854	0.0238681	1	http://jaspar.genereg.net/matrix/MA1185.1

MYB111	MA1036.1	1.96732	0.0245001	1	http://jaspar.genereg.net/matrix/MA1036.1
bZIP28	MA1344.1	1.95397	0.0252851	1	http://jaspar.genereg.net/matrix/MA1344.1
AGL15	MA0548.2	1.94983	0.02552	1	http://jaspar.genereg.net/matrix/MA0548.2
squamosa	MA0082.1	1.94538	0.0258194	1	http://jaspar.genereg.net/matrix/MA0082.1
PHYPADRAFT_182268	MA1008.1	1.93285	0.0265613	1	http://jaspar.genereg.net/matrix/MA1008.1
CBF2	MA1217.1	1.91363	0.027756	1	http://jaspar.genereg.net/matrix/MA1217.1
DREB1G	MA1032.1	1.87165	0.0305411	1	http://jaspar.genereg.net/matrix/MA1032.1
OBP3	MA1274.1	1.86799	0.0308218	1	http://jaspar.genereg.net/matrix/MA1274.1
DREB1A	MA0971.1	1.8564	0.0316128	1	http://jaspar.genereg.net/matrix/MA0971.1
CBF1	MA1224.1	1.85005	0.032058	1	http://jaspar.genereg.net/matrix/MA1224.1
ARR2	MA0949.1	1.84494	0.0324721	1	http://jaspar.genereg.net/matrix/MA0949.1
AT5G56840	MA1195.1	1.84414	0.0325112	1	http://jaspar.genereg.net/matrix/MA1195.1
AHL20	MA0933.1	1.83921	0.0328953	1	http://jaspar.genereg.net/matrix/MA0933.1
bZIP68	MA0968.1	1.82181	0.0341702	1	http://jaspar.genereg.net/matrix/MA0968.1
AT3G10580	MA1194.1	1.80526	0.0354225	1	http://jaspar.genereg.net/matrix/MA1194.1
AT1G69570	MA1268.1	1.80428	0.0355307	1	http://jaspar.genereg.net/matrix/MA1268.1
dof4.2	MA1273.1	1.7895	0.0366867	1	http://jaspar.genereg.net/matrix/MA1273.1
AT5G02460	MA1281.1	1.76417	0.0387962	1	http://jaspar.genereg.net/matrix/MA1281.1
EPR1	MA1401.1	1.7192	0.0427038	1	http://jaspar.genereg.net/matrix/MA1401.1
AHL25	MA0934.1	1.70499	0.04404	1	http://jaspar.genereg.net/matrix/MA0934.1

bHLH18	MA1361.1	1.70022	0.044386<br>8	1	http://jaspar.genereg.net/matrix/MA1<br>361.1
CBF4	MA1218.1	1.66113	0.048229<br>1	1	http://jaspar.genereg.net/matrix/MA1<br>218.1
AT5G61620	MA1189.1	1.64608	0.049773<br>3	1	http://jaspar.genereg.net/matrix/MA1<br>189.1
ERF008	MA0979.1	1.64004	0.050423<br>1	1	http://jaspar.genereg.net/matrix/MA0<br>979.1
RVE1	MA1184.1	1.61232	0.053349<br>2	1	http://jaspar.genereg.net/matrix/MA1<br>184.1
AT3G10113	MA1191.1	1.60635	0.054002<br>9	1	http://jaspar.genereg.net/matrix/MA1<br>191.1
ARR10	MA0121.1	1.60684	0.054003<br>6	1	http://jaspar.genereg.net/matrix/MA0<br>121.1
PLT1	MA1377.1	1.5657	0.058537<br>6	1	http://jaspar.genereg.net/matrix/MA1<br>377.1
AGL55	MA1202.1	1.55053	0.060382<br>7	1	http://jaspar.genereg.net/matrix/MA1<br>202.1
AHL12	MA0932.1	1.53711	0.062081<br>3	1	http://jaspar.genereg.net/matrix/MA0<br>932.1
MYB46	MA1040.1	1.49863	0.066890<br>6	1	http://jaspar.genereg.net/matrix/MA1<br>040.1
AT5G66940	MA1267.1	1.49229	0.067751	1	http://jaspar.genereg.net/matrix/MA1<br>267.1
AGL13	MA1204.1	1.48576	0.068562<br>3	1	http://jaspar.genereg.net/matrix/MA1<br>204.1
FaEOBII	MA1408.1	1.48558	0.068574<br>1	1	http://jaspar.genereg.net/matrix/MA1<br>408.1
ERF039	MA0995.1	1.47385	0.070136<br>8	1	http://jaspar.genereg.net/matrix/MA0<br>995.1
AREB3	MA1338.1	1.46897	0.070780<br>9	1	http://jaspar.genereg.net/matrix/MA1<br>338.1
AT1G19210	MA1234.1	1.46352	0.071573<br>7	1	http://jaspar.genereg.net/matrix/MA1<br>234.1
bZIP68	MA1347.1	1.46295	0.071621<br>4	1	http://jaspar.genereg.net/matrix/MA1<br>347.1
MYB24	MA1037.1	1.46015	0.072022<br>5	1	http://jaspar.genereg.net/matrix/MA1<br>037.1
UIF1	MA1413.1	1.45437	0.072815<br>7	1	http://jaspar.genereg.net/matrix/MA1<br>413.1

ARR1	MA0945.1	1.44409	0.0742791	1	http://jaspar.genereg.net/matrix/MA0945.1
Adof1	MA1277.1	1.38987	0.0821877	1	http://jaspar.genereg.net/matrix/MA1277.1
ATHB18	MA1211.1	1.38879	0.0823551	1	http://jaspar.genereg.net/matrix/MA1211.1
bHLH69	MA1363.1	1.3847	0.0828523	1	http://jaspar.genereg.net/matrix/MA1363.1
AT3G45610	MA1270.1	1.37476	0.0845321	1	http://jaspar.genereg.net/matrix/MA1270.1
PHYPADRAFT_173530	MA1007.1	1.36962	0.0852812	1	http://jaspar.genereg.net/matrix/MA1007.1
AT4G28140	MA1232.1	1.34888	0.0885988	1	http://jaspar.genereg.net/matrix/MA1232.1
AT2G28810	MA1272.1	1.33344	0.0911196	1	http://jaspar.genereg.net/matrix/MA1272.1
At5g47390	MA1398.1	1.3212	0.0930919	1	http://jaspar.genereg.net/matrix/MA1398.1
PHYPADRAFT_28324	MA1023.1	1.32105	0.0931226	1	http://jaspar.genereg.net/matrix/MA1023.1
NAC083	MA1043.1	1.30781	0.0953478	1	http://jaspar.genereg.net/matrix/MA1043.1
KAN1	MA1027.1	1.28547	0.099224	1	http://jaspar.genereg.net/matrix/MA1027.1
AT4G37180	MA1164.1	1.27852	0.100396	1	http://jaspar.genereg.net/matrix/MA1164.1
ARF5	MA0943.1	1.27001	0.101911	1	http://jaspar.genereg.net/matrix/MA0943.1
ATHB7	MA0954.2	1.2602	0.103717	1	http://jaspar.genereg.net/matrix/MA0954.2
AT1G25550	MA1386.1	1.25146	0.105256	1	http://jaspar.genereg.net/matrix/MA1386.1
OBP1	MA1278.1	1.22285	0.110617	1	http://jaspar.genereg.net/matrix/MA1278.1
At1g19000	MA1400.1	1.18723	0.117474	1	http://jaspar.genereg.net/matrix/MA1400.1
bZIP16	MA1349.1	1.18003	0.118835	1	http://jaspar.genereg.net/matrix/MA1349.1
DREB26	MA1248.1	1.16337	0.122225	1	http://jaspar.genereg.net/matrix/MA1248.1

ERF096	MA0998.1	1.16038	0.122807	1	http://jaspar.genereg.net/matrix/MA0998.1
JKD	MA1156.1	1.13102	0.128901	1	http://jaspar.genereg.net/matrix/MA1156.1
AGL27	MA1012.1	1.1266	0.129873	1	http://jaspar.genereg.net/matrix/MA1012.1
MYB52	MA1171.1	1.12445	0.130295	1	http://jaspar.genereg.net/matrix/MA1171.1
ERF13	MA1004.1	1.11369	0.132567	1	http://jaspar.genereg.net/matrix/MA1004.1
ARF2	MA1206.1	1.09994	0.135585	1	http://jaspar.genereg.net/matrix/MA1206.1
MYB55	MA1041.1	1.09983	0.135592	1	http://jaspar.genereg.net/matrix/MA1041.1
At5g08520	MA1399.1	1.0997	0.135626	1	http://jaspar.genereg.net/matrix/MA1399.1
AT2G44940	MA1229.1	1.09888	0.135758	1	http://jaspar.genereg.net/matrix/MA1229.1
AGL16	MA1199.1	1.06647	0.142979	1	http://jaspar.genereg.net/matrix/MA1199.1
ANT	MA0571.1	1.06385	0.14356	1	http://jaspar.genereg.net/matrix/MA0571.1
GBF3	MA1351.1	1.04563	0.147719	1	http://jaspar.genereg.net/matrix/MA1351.1
AT1G36060	MA1263.1	1.04425	0.148073	1	http://jaspar.genereg.net/matrix/MA1263.1
SPL7	MA1060.1	1.04067	0.148866	1	http://jaspar.genereg.net/matrix/MA1060.1
MYB1	MA1179.1	1.03078	0.15121	1	http://jaspar.genereg.net/matrix/MA1179.1
DEAR5	MA1251.1	1.03034	0.151281	1	http://jaspar.genereg.net/matrix/MA1251.1
HAT22	MA1210.1	1.01688	0.154513	1	http://jaspar.genereg.net/matrix/MA1210.1
ARF8	MA0944.1	1.01326	0.155354	1	http://jaspar.genereg.net/matrix/MA0944.1
DREB2C	MA0986.1	1.00651	0.156964	1	http://jaspar.genereg.net/matrix/MA0986.1
ATHB34	MA1328.1	1.00042	0.15847	1	http://jaspar.genereg.net/matrix/MA1328.1

NAC080	MA0939.1	0.981397	0.162997	1	http://jaspar.genereg.net/matrix/MA0939.1
COG1	MA1279.1	0.98077	0.163277	1	http://jaspar.genereg.net/matrix/MA1279.1
ATHB21	MA1213.1	0.956055	0.169389	1	http://jaspar.genereg.net/matrix/MA1213.1
AT3G24120	MA1388.1	0.945141	0.172179	1	http://jaspar.genereg.net/matrix/MA1388.1
ATHB23	MA1327.1	0.94066	0.173356	1	http://jaspar.genereg.net/matrix/MA1327.1
AP3	MA0556.1	0.939383	0.173678	1	http://jaspar.genereg.net/matrix/MA0556.1
AT1G49560	MA1165.1	0.935169	0.174726	1	http://jaspar.genereg.net/matrix/MA1165.1
CCA1	MA0972.1	0.934408	0.174917	1	http://jaspar.genereg.net/matrix/MA0972.1
At5g05790	MA1196.1	0.932058	0.175515	1	http://jaspar.genereg.net/matrix/MA1196.1
MYB105	MA1169.1	0.924105	0.177608	1	http://jaspar.genereg.net/matrix/MA1169.1
NUC	MA1157.1	0.911362	0.180931	1	http://jaspar.genereg.net/matrix/MA1157.1
RAP210	MA1249.1	0.907989	0.181784	1	http://jaspar.genereg.net/matrix/MA1249.1
MYB3	MA1038.1	0.895009	0.185224	1	http://jaspar.genereg.net/matrix/MA1038.1
T11I18.17	MA0936.1	0.891603	0.186171	1	http://jaspar.genereg.net/matrix/MA0936.1
MYB62	MA1294.1	0.88961	0.186689	1	http://jaspar.genereg.net/matrix/MA1294.1
ATHB-5	MA0110.2	0.889378	0.186765	1	http://jaspar.genereg.net/matrix/MA0110.2
HDG1	MA1369.1	0.888702	0.186972	1	http://jaspar.genereg.net/matrix/MA1369.1
MGP	MA1158.1	0.886135	0.187651	1	http://jaspar.genereg.net/matrix/MA1158.1
CDC5	MA0579.1	0.883632	0.188375	1	http://jaspar.genereg.net/matrix/MA0579.1
ARF1	MA0942.1	0.880502	0.189134	1	http://jaspar.genereg.net/matrix/MA0942.1

DRE1C	MA0985.1	0.878011	0.189808	1	http://jaspar.genereg.net/matrix/MA0985.1
ANL2	MA1375.1	0.868991	0.19232	1	http://jaspar.genereg.net/matrix/MA1375.1
AT3G16280	MA1253.1	0.852988	0.196693	1	http://jaspar.genereg.net/matrix/MA1253.1
OsRR22	MA1409.1	0.849396	0.197728	1	http://jaspar.genereg.net/matrix/MA1409.1
AT4G12670	MA1354.1	0.849155	0.197798	1	http://jaspar.genereg.net/matrix/MA1354.1
AGL6	MA1205.1	0.845825	0.198687	1	http://jaspar.genereg.net/matrix/MA1205.1
SVP	MA0555.1	0.843576	0.199383	1	http://jaspar.genereg.net/matrix/MA0555.1
ERF098	MA0999.1	0.831653	0.202665	1	http://jaspar.genereg.net/matrix/MA0999.1
TINY	MA1220.1	0.824708	0.204627	1	http://jaspar.genereg.net/matrix/MA1220.1
AT3G04030	MA1168.1	0.805993	0.209976	1	http://jaspar.genereg.net/matrix/MA1168.1
MYB3R5	MA1172.1	0.80599	0.210026	1	http://jaspar.genereg.net/matrix/MA1172.1
DEAR3	MA1376.1	0.799253	0.211952	1	http://jaspar.genereg.net/matrix/MA1376.1
PEND	MA0127.1	0.791751	0.214115	1	http://jaspar.genereg.net/matrix/MA0127.1
At5g58900	MA1192.1	0.791574	0.214142	1	http://jaspar.genereg.net/matrix/MA1192.1
ATHB4	MA1406.1	0.790103	0.214639	1	http://jaspar.genereg.net/matrix/MA1406.1
DREB1E	MA0978.1	0.784308	0.216265	1	http://jaspar.genereg.net/matrix/MA0978.1
ERF8	MA0994.1	0.769105	0.220797	1	http://jaspar.genereg.net/matrix/MA0994.1
At1g74840	MA1397.1	0.769044	0.220841	1	http://jaspar.genereg.net/matrix/MA1397.1
HAT2	MA1198.1	0.764893	0.222074	1	http://jaspar.genereg.net/matrix/MA1198.1
NAC92	MA1044.1	0.756272	0.224603	1	http://jaspar.genereg.net/matrix/MA1044.1

ATHB25	MA1329.1	0.755029	0.225044	1	http://jaspar.genereg.net/matrix/MA1329.1
GATA11	MA1014.1	0.749656	0.226622	1	http://jaspar.genereg.net/matrix/MA1014.1
Dof2	MA0020.1	0.748031	0.227143	1	http://jaspar.genereg.net/matrix/MA0020.1
SPL13	MA1321.1	0.74581	0.227776	1	http://jaspar.genereg.net/matrix/MA1321.1
At3g11280	MA1188.1	0.7345	0.231175	1	http://jaspar.genereg.net/matrix/MA1188.1
TRP1	MA1352.1	0.727119	0.233516	1	http://jaspar.genereg.net/matrix/MA1352.1
NAC025	MA0935.1	0.719575	0.23579	1	http://jaspar.genereg.net/matrix/MA0935.1
REF6	MA1415.1	0.718413	0.236124	1	http://jaspar.genereg.net/matrix/MA1415.1
MYB70	MA1393.1	0.717181	0.236524	1	http://jaspar.genereg.net/matrix/MA1393.1
MNB1A	MA0053.1	0.691875	0.244432	1	http://jaspar.genereg.net/matrix/MA0053.1
SEP3	MA0563.1	0.681914	0.247596	1	http://jaspar.genereg.net/matrix/MA0563.1
F3A4.140	MA0575.1	0.667731	0.252071	1	http://jaspar.genereg.net/matrix/MA0575.1
ERF043	MA0996.1	0.667352	0.252143	1	http://jaspar.genereg.net/matrix/MA0996.1
MYB113	MA1181.1	0.666086	0.252562	1	http://jaspar.genereg.net/matrix/MA1181.1
HMG-I/Y	MA0045.1	0.649188	0.258023	1	http://jaspar.genereg.net/matrix/MA0045.1
ATHB-12	MA0950.1	0.625074	0.265878	1	http://jaspar.genereg.net/matrix/MA0950.1
TRP2	MA1356.1	0.624964	0.265912	1	http://jaspar.genereg.net/matrix/MA1356.1
ARR14	MA0947.1	0.620722	0.267295	1	http://jaspar.genereg.net/matrix/MA0947.1
MYB65	MA1177.1	0.613633	0.269671	1	http://jaspar.genereg.net/matrix/MA1177.1
bZIP3	MA1340.1	0.606967	0.271835	1	http://jaspar.genereg.net/matrix/MA1340.1

ERF11	MA1001.1	0.600497	0.273971	1	http://jaspar.genereg.net/matrix/MA1001.1
AT1G77200	MA1230.1	0.599658	0.27424	1	http://jaspar.genereg.net/matrix/MA1230.1
SPL12	MA1057.1	0.593405	0.27639	1	http://jaspar.genereg.net/matrix/MA1057.1
AT1G14580	MA1160.1	0.590667	0.277254	1	http://jaspar.genereg.net/matrix/MA1160.1
AP1	MA0940.1	0.589837	0.277579	1	http://jaspar.genereg.net/matrix/MA0940.1
MYB59	MA1042.1	0.578792	0.281251	1	http://jaspar.genereg.net/matrix/MA1042.1
AT2G40260	MA1385.1	0.575296	0.282457	1	http://jaspar.genereg.net/matrix/MA1385.1
SPL1	MA1055.1	0.573029	0.283207	1	http://jaspar.genereg.net/matrix/MA1055.1
ERF7	MA0993.1	0.569331	0.284459	1	http://jaspar.genereg.net/matrix/MA0993.1
MYB27	MA1292.1	0.557237	0.288544	1	http://jaspar.genereg.net/matrix/MA1292.1
HAT1	MA1024.1	0.546115	0.292417	1	http://jaspar.genereg.net/matrix/MA1024.1
MYB56	MA1174.1	0.544714	0.292882	1	http://jaspar.genereg.net/matrix/MA1174.1
AT1G12630	MA1227.1	0.540145	0.294427	1	http://jaspar.genereg.net/matrix/MA1227.1
DREB2	MA1258.1	0.520185	0.301359	1	http://jaspar.genereg.net/matrix/MA1258.1
PBF	MA0064.1	0.50412	0.307023	1	http://jaspar.genereg.net/matrix/MA0064.1
NTL9	MA1046.1	0.495932	0.309913	1	http://jaspar.genereg.net/matrix/MA1046.1
SPL9	MA1322.1	0.494856	0.310271	1	http://jaspar.genereg.net/matrix/MA1322.1
AGL42	MA1201.1	0.47408	0.317648	1	http://jaspar.genereg.net/matrix/MA1201.1
AT1G13300	MA1387.1	0.455141	0.324412	1	http://jaspar.genereg.net/matrix/MA1387.1
ARF3	MA1009.1	0.454731	0.324565	1	http://jaspar.genereg.net/matrix/MA1009.1

PLT3	MA1378.1	0.451954	0.325531	1	http://jaspar.genereg.net/matrix/MA1378.1
AT3G60490	MA1223.1	0.448781	0.326699	1	http://jaspar.genereg.net/matrix/MA1223.1
ARR18	MA0948.1	0.448073	0.326972	1	http://jaspar.genereg.net/matrix/MA0948.1
EDT1	MA0990.1	0.443778	0.328531	1	http://jaspar.genereg.net/matrix/MA0990.1
SPL3	MA1319.1	0.438608	0.330414	1	http://jaspar.genereg.net/matrix/MA1319.1
MYB3R1	MA1178.1	0.437948	0.330655	1	http://jaspar.genereg.net/matrix/MA1178.1
OBP4	MA1280.1	0.431156	0.333121	1	http://jaspar.genereg.net/matrix/MA1280.1
ATHB-6	MA0953.1	0.421988	0.336465	1	http://jaspar.genereg.net/matrix/MA0953.1
DREB19	MA1243.1	0.410891	0.340484	1	http://jaspar.genereg.net/matrix/MA1243.1
GT2	MA1208.1	0.408734	0.341301	1	http://jaspar.genereg.net/matrix/MA1208.1
ERF38	MA1238.1	0.408557	0.341346	1	http://jaspar.genereg.net/matrix/MA1238.1
ATHB40	MA1214.1	0.405829	0.342357	1	http://jaspar.genereg.net/matrix/MA1214.1
ATHB15	MA1026.2	0.403232	0.343298	1	http://jaspar.genereg.net/matrix/MA1026.2
ERF112	MA1002.1	0.389923	0.348208	1	http://jaspar.genereg.net/matrix/MA1002.1
GATA9	MA1018.1	0.388465	0.348763	1	http://jaspar.genereg.net/matrix/MA1018.1
AT5G47660	MA1365.1	0.383714	0.35053	1	http://jaspar.genereg.net/matrix/MA1365.1
Gam1	MA0034.1	0.372734	0.354594	1	http://jaspar.genereg.net/matrix/MA0034.1
TSO1	MA1161.1	0.369163	0.355931	1	http://jaspar.genereg.net/matrix/MA1161.1
SPL11	MA1056.1	0.365795	0.357165	1	http://jaspar.genereg.net/matrix/MA1056.1
IDD2	MA1373.1	0.364167	0.357802	1	http://jaspar.genereg.net/matrix/MA1373.1

ATHB-16	MA0951.1	0.359803	0.359421	1	http://jaspar.genereg.net/matrix/MA0951.1
POPTR_0002s00440g	MA0955.1	0.354144	0.361549	1	http://jaspar.genereg.net/matrix/MA0955.1
SEP1	MA0584.1	0.352028	0.362345	1	http://jaspar.genereg.net/matrix/MA0584.1
DYT1	MA0580.1	0.34836	0.36369	1	http://jaspar.genereg.net/matrix/MA0580.1
NAC043	MA1045.1	0.344693	0.365117	1	http://jaspar.genereg.net/matrix/MA1045.1
RAP21	MA1216.1	0.339948	0.366884	1	http://jaspar.genereg.net/matrix/MA1216.1
CMTA3	MA0970.1	0.325236	0.372434	1	http://jaspar.genereg.net/matrix/MA0970.1
AT2G20110	MA1380.1	0.311893	0.377521	1	http://jaspar.genereg.net/matrix/MA1380.1
SPL4	MA1058.1	0.307834	0.379043	1	http://jaspar.genereg.net/matrix/MA1058.1
GATA8	MA1017.1	0.28736	0.386876	1	http://jaspar.genereg.net/matrix/MA1017.1
DOF5.3	MA1071.1	0.284222	0.388041	1	http://jaspar.genereg.net/matrix/MA1071.1
PI	MA0559.1	0.284059	0.38815	1	http://jaspar.genereg.net/matrix/MA0559.1
PHYPADRAFT_64121	MA1010.1	0.280184	0.389623	1	http://jaspar.genereg.net/matrix/MA1010.1
At1g49010	MA1186.1	0.276363	0.39108	1	http://jaspar.genereg.net/matrix/MA1186.1
id1	MA0120.1	0.27502	0.391617	1	http://jaspar.genereg.net/matrix/MA0120.1
ERF13	MA1254.1	0.273928	0.39202	1	http://jaspar.genereg.net/matrix/MA1254.1
SGR5	MA1159.1	0.272516	0.39257	1	http://jaspar.genereg.net/matrix/MA1159.1
AG	MA0005.2	0.26626	0.394939	1	http://jaspar.genereg.net/matrix/MA0005.2
MYB3R4	MA1180.1	0.258096	0.398128	1	http://jaspar.genereg.net/matrix/MA1180.1
IDD5	MA1370.1	0.255891	0.398967	1	http://jaspar.genereg.net/matrix/MA1370.1

CEJ1	MA1219.1	0.248732	0.401738	1	http://jaspar.genereg.net/matrix/MA1219.1
MYB15	MA0574.1	0.239037	0.405493	1	http://jaspar.genereg.net/matrix/MA0574.1
GT-1	MA1020.1	0.233632	0.407599	1	http://jaspar.genereg.net/matrix/MA1020.1
DAG2	MA1271.1	0.231042	0.408611	1	http://jaspar.genereg.net/matrix/MA1271.1
ERF3	MA1005.1	0.226179	0.410486	1	http://jaspar.genereg.net/matrix/MA1005.1
ERF1B	MA0567.1	0.224439	0.411163	1	http://jaspar.genereg.net/matrix/MA0567.1
NAC055	MA0937.1	0.217689	0.413798	1	http://jaspar.genereg.net/matrix/MA0937.1
ERF4	MA0992.1	0.207551	0.417744	1	http://jaspar.genereg.net/matrix/MA0992.1
AT1G76880	MA1366.1	0.187222	0.425707	1	http://jaspar.genereg.net/matrix/MA1366.1
IDD7	MA1374.1	0.183135	0.42731	1	http://jaspar.genereg.net/matrix/MA1374.1
AT4G16750	MA1237.1	0.179627	0.428687	1	http://jaspar.genereg.net/matrix/MA1237.1
GATA10	MA1013.1	0.1768	0.429794	1	http://jaspar.genereg.net/matrix/MA1013.1
AT5G45580	MA1163.1	0.174597	0.430659	1	http://jaspar.genereg.net/matrix/MA1163.1
ERF094	MA1049.1	0.17431	0.430766	1	http://jaspar.genereg.net/matrix/MA1049.1
AT1G76870	MA1367.1	0.164284	0.434714	1	http://jaspar.genereg.net/matrix/MA1367.1
AT2G03500	MA1167.1	0.156659	0.437732	1	http://jaspar.genereg.net/matrix/MA1167.1
CRF4	MA0976.1	0.142959	0.443127	1	http://jaspar.genereg.net/matrix/MA0976.1
AT1G68670	MA1390.1	0.132202	0.447383	1	http://jaspar.genereg.net/matrix/MA1390.1
KAN4	MA1028.1	0.131868	0.447521	1	http://jaspar.genereg.net/matrix/MA1028.1
LFY	MA0590.1	0.0989984	0.460555	1	http://jaspar.genereg.net/matrix/MA0590.1

ATHB33	MA1326.1	0.0809965	0.467712	1	http://jaspar.genereg.net/matrix/MA1326.1
ATHB53	MA1215.1	0.0795817	0.468269	1	http://jaspar.genereg.net/matrix/MA1215.1
MYB57	MA1293.1	0.0761688	0.46962	1	http://jaspar.genereg.net/matrix/MA1293.1
GATA20	MA1324.1	0.0671003	0.473233	1	http://jaspar.genereg.net/matrix/MA1324.1
IDD4	MA1371.1	0.0441387	0.482388	1	http://jaspar.genereg.net/matrix/MA1371.1
SPL14	MA0586.2	0.037044	0.485218	1	http://jaspar.genereg.net/matrix/MA0586.2
STZ	MA1372.1	0.037022	0.485225	1	http://jaspar.genereg.net/matrix/MA1372.1
CMTA2	MA0969.1	0.0366599	0.485371	1	http://jaspar.genereg.net/matrix/MA0969.1
bZIP52	MA1343.1	0.00766878	0.496939	1	http://jaspar.genereg.net/matrix/MA1343.1
ATHB-51	MA0952.1	0.00639641	0.497447	1	http://jaspar.genereg.net/matrix/MA0952.1
O2	MA1417.1	-0.0512707	0.520452	1	http://jaspar.genereg.net/matrix/MA1417.1
SOC1	MA0554.1	-0.0522819	0.520853	1	http://jaspar.genereg.net/matrix/MA0554.1
Dof3	MA0021.1	-0.0581923	0.523214	1	http://jaspar.genereg.net/matrix/MA0021.1
RAX3	MA0576.1	-0.0606018	0.524175	1	http://jaspar.genereg.net/matrix/MA0576.1
AIL7	MA1235.1	-0.0667928	0.526646	1	http://jaspar.genereg.net/matrix/MA1235.1
GATA12	MA1015.1	-0.0895709	0.535701	1	http://jaspar.genereg.net/matrix/MA1015.1

TCX2	MA1162.1	-0.0989892	0.539442	1	http://jaspar.genereg.net/matrix/MA1162.1
SOL1	MA1379.1	-0.106186	0.542296	1	http://jaspar.genereg.net/matrix/MA1379.1
MYB77	MA1395.1	-0.109833	0.543741	1	http://jaspar.genereg.net/matrix/MA1395.1
AT4G32800	MA1241.1	-0.113361	0.545156	1	http://jaspar.genereg.net/matrix/MA1241.1
ERF069	MA0997.1	-0.117881	0.546951	1	http://jaspar.genereg.net/matrix/MA0997.1
AGL63	MA1203.1	-0.132984	0.552912	1	http://jaspar.genereg.net/matrix/MA1203.1
FLC	MA0558.1	-0.138932	0.555265	1	http://jaspar.genereg.net/matrix/MA0558.1
E2FA	MA1414.1	-0.143748	0.557183	1	http://jaspar.genereg.net/matrix/MA1414.1
AGL3	MA0001.2	-0.149562	0.55947	1	http://jaspar.genereg.net/matrix/MA0001.2
RAP2-3	MA1051.1	-0.161296	0.564104	1	http://jaspar.genereg.net/matrix/MA1051.1
ATHB20	MA1209.1	-0.197416	0.578291	1	http://jaspar.genereg.net/matrix/MA1209.1
CRF2	MA0975.1	-0.203388	0.58063	1	http://jaspar.genereg.net/matrix/MA0975.1
bZIP44	MA1337.1	-0.206936	0.582005	1	http://jaspar.genereg.net/matrix/MA1337.1
RAP211	MA1266.1	-0.209026	0.582827	1	http://jaspar.genereg.net/matrix/MA1266.1
RAP2-10	MA0980.1	-0.209125	0.582873	1	http://jaspar.genereg.net/matrix/MA0980.1
AGL1	MA0585.1	-0.21222	0.584089	1	http://jaspar.genereg.net/matrix/MA0585.1
ATHB24	MA1330.1	-0.213281	0.584477	1	http://jaspar.genereg.net/matrix/MA1330.1
ATHB13	MA1212.1	-0.228958	0.590589	1	http://jaspar.genereg.net/matrix/MA1212.1
AGL25	MA1200.1	-0.242549	0.595863	1	http://jaspar.genereg.net/matrix/MA1200.1

HAT5	MA0008.2	-0.257641	0.601696	1	http://jaspar.genereg.net/matrix/MA0008.2
SPL5	MA1059.2	-0.259874	0.602564	1	http://jaspar.genereg.net/matrix/MA1059.2
AT1G01250	MA1259.1	-0.279683	0.6102	1	http://jaspar.genereg.net/matrix/MA1259.1
GATA14	MA1325.1	-0.305549	0.620084	1	http://jaspar.genereg.net/matrix/MA1325.1
AT4G31060	MA1265.1	-0.307509	0.620813	1	http://jaspar.genereg.net/matrix/MA1265.1
ATHB-9	MA0573.1	-0.308454	0.621194	1	http://jaspar.genereg.net/matrix/MA0573.1
SPL15	MA1320.1	-0.309445	0.621567	1	http://jaspar.genereg.net/matrix/MA1320.1
SMZ	MA0553.1	-0.337016	0.631994	1	http://jaspar.genereg.net/matrix/MA0553.1
SPL8	MA0578.1	-0.359465	0.640418	1	http://jaspar.genereg.net/matrix/MA0578.1
GBF6	MA1334.1	-0.367626	0.643486	1	http://jaspar.genereg.net/matrix/MA1334.1
MYB119	MA1176.1	-0.371834	0.645054	1	http://jaspar.genereg.net/matrix/MA1176.1
ABF1	MA0570.1	-0.378211	0.647451	1	http://jaspar.genereg.net/matrix/MA0570.1
RAV1(var.2)	MA0583.1	-0.380358	0.648213	1	http://jaspar.genereg.net/matrix/MA0583.1
AT1G75490	MA1250.1	-0.407143	0.658095	1	http://jaspar.genereg.net/matrix/MA1250.1
WRKY62	MA1091.1	-0.40999	0.659135	1	http://jaspar.genereg.net/matrix/MA1091.1
AT1G77640	MA1260.1	-0.425542	0.664824	1	http://jaspar.genereg.net/matrix/MA1260.1
BPC5	MA1403.1	-0.429177	0.666169	1	http://jaspar.genereg.net/matrix/MA1403.1
MYB98	MA1392.1	-0.430853	0.666779	1	http://jaspar.genereg.net/matrix/MA1392.1
bZIP53	MA1341.1	-0.444869	0.671865	1	http://jaspar.genereg.net/matrix/MA1341.1
ERF109	MA1053.1	-0.454568	0.675383	1	http://jaspar.genereg.net/matrix/MA1053.1

RAP2-6	MA1052.1	-0.470837	0.681222	1	http://jaspar.genereg.net/matrix/MA1052.1
KAN2	MA1383.1	-0.480475	0.684642	1	http://jaspar.genereg.net/matrix/MA1383.1
AT3G25990	MA1368.1	-0.482387	0.685296	1	http://jaspar.genereg.net/matrix/MA1368.1
myb.Ph3	MA0054.1	-0.498945	0.691172	1	http://jaspar.genereg.net/matrix/MA0054.1
DOF2.4	MA0982.1	-0.503112	0.692643	1	http://jaspar.genereg.net/matrix/MA0982.1
AT1G47655	MA1275.1	-0.504508	0.693123	1	http://jaspar.genereg.net/matrix/MA1275.1
AT3G46070	MA1381.1	-0.515628	0.697032	1	http://jaspar.genereg.net/matrix/MA1381.1
AT3G52440	MA1276.1	-0.518956	0.698174	1	http://jaspar.genereg.net/matrix/MA1276.1
MYB101	MA1173.1	-0.525076	0.700295	1	http://jaspar.genereg.net/matrix/MA1173.1
AT2G01060	MA1384.1	-0.530462	0.702177	1	http://jaspar.genereg.net/matrix/MA1384.1
TGA2	MA1068.1	-0.531553	0.70252	1	http://jaspar.genereg.net/matrix/MA1068.1
MYB118	MA1170.1	-0.534585	0.70361	1	http://jaspar.genereg.net/matrix/MA1170.1
dof4.5	MA1269.1	-0.53804	0.704795	1	http://jaspar.genereg.net/matrix/MA1269.1
GATA15	MA1016.1	-0.547377	0.708044	1	http://jaspar.genereg.net/matrix/MA1016.1
BPC1	MA1404.1	-0.548515	0.708401	1	http://jaspar.genereg.net/matrix/MA1404.1
AT1G44830	MA1222.1	-0.555625	0.710853	1	http://jaspar.genereg.net/matrix/MA1222.1
ARALYDRAFT_493022	MA1097.1	-0.561169	0.712745	1	http://jaspar.genereg.net/matrix/MA1097.1
MYB73	MA1394.1	-0.569745	0.715609	1	http://jaspar.genereg.net/matrix/MA1394.1
AT5G67000	MA1261.1	-0.570211	0.715822	1	http://jaspar.genereg.net/matrix/MA1261.1
MYB33	MA1391.1	-0.584677	0.720677	1	http://jaspar.genereg.net/matrix/MA1391.1

bZIP910	MA0096.1	-0.587721	0.721679	1	http://jaspar.genereg.net/matrix/MA0096.1
BPC6	MA1402.1	-0.588844	0.722072	1	http://jaspar.genereg.net/matrix/MA1402.1
DOF5.6	MA0983.1	-0.613707	0.730387	1	http://jaspar.genereg.net/matrix/MA0983.1
bZIP43	MA1339.1	-0.620157	0.732503	1	http://jaspar.genereg.net/matrix/MA1339.1
HMG-1	MA0044.1	-0.624086	0.733799	1	http://jaspar.genereg.net/matrix/MA0044.1
AT3G12730	MA1166.1	-0.632303	0.736485	1	http://jaspar.genereg.net/matrix/MA1166.1
DOF5.7	MA0984.1	-0.644698	0.740515	1	http://jaspar.genereg.net/matrix/MA0984.1
AT5G29000	MA1389.1	-0.648853	0.741893	1	http://jaspar.genereg.net/matrix/MA1389.1
TGA1	MA0588.1	-0.650385	0.742341	1	http://jaspar.genereg.net/matrix/MA0588.1
CAMTA1	MA1197.1	-0.699702	0.758034	1	http://jaspar.genereg.net/matrix/MA1197.1
GATA19	MA1323.1	-0.751137	0.773797	1	http://jaspar.genereg.net/matrix/MA1323.1
AT3G57600	MA1242.1	-0.78481	0.783777	1	http://jaspar.genereg.net/matrix/MA1242.1
bZIP42	MA1350.1	-0.785852	0.78411	1	http://jaspar.genereg.net/matrix/MA1350.1
SIZF2	MA1405.1	-0.798599	0.787843	1	http://jaspar.genereg.net/matrix/MA1405.1
AT1G71450	MA1233.1	-0.807597	0.790434	1	http://jaspar.genereg.net/matrix/MA1233.1
TGA1A	MA0129.1	-0.825215	0.795439	1	http://jaspar.genereg.net/matrix/MA0129.1
TGA5	MA1047.1	-0.825552	0.795527	1	http://jaspar.genereg.net/matrix/MA1047.1
ARALYDRAFT_496250	MA1096.1	-0.83504	0.798316	1	http://jaspar.genereg.net/matrix/MA1096.1
AT1G22810	MA1255.1	-0.868052	0.807401	1	http://jaspar.genereg.net/matrix/MA1255.1
bZIP48	MA1345.1	-0.879497	0.810528	1	http://jaspar.genereg.net/matrix/MA1345.1

ZAP1	MA0589.1	-0.880306	0.810728	1	http://jaspar.genereg.net/matrix/MA0589.1
RAV1	MA0582.1	-0.880764	0.810897	1	http://jaspar.genereg.net/matrix/MA0582.1
DOF1.8	MA0981.1	-0.895325	0.814808	1	http://jaspar.genereg.net/matrix/MA0981.1
PHYPADRAFT_140773	MA0987.1	-0.914832	0.819985	1	http://jaspar.genereg.net/matrix/MA0987.1
bZIP14	MA1407.1	-0.920471	0.821406	1	http://jaspar.genereg.net/matrix/MA1407.1
Glyma19g26560.1	MA1019.1	-0.924035	0.822356	1	http://jaspar.genereg.net/matrix/MA1019.1
TCP3	MA1289.1	-0.936333	0.825566	1	http://jaspar.genereg.net/matrix/MA1289.1
TCP4	MA1035.1	-0.94272	0.827232	1	http://jaspar.genereg.net/matrix/MA1035.1
AT4G18450	MA1228.1	-0.991899	0.839448	1	http://jaspar.genereg.net/matrix/MA1228.1
TCP7	MA1291.1	-0.993269	0.839789	1	http://jaspar.genereg.net/matrix/MA1291.1
MYB81	MA1175.1	-1.0014	0.841736	1	http://jaspar.genereg.net/matrix/MA1175.1
RAMOSA1	MA1416.1	-1.00813	0.843383	1	http://jaspar.genereg.net/matrix/MA1416.1
AT5G18450	MA1226.1	-1.02672	0.847803	1	http://jaspar.genereg.net/matrix/MA1226.1
TCP15	MA1062.1	-1.05131	0.853532	1	http://jaspar.genereg.net/matrix/MA1062.1
TCP23	MA1066.1	-1.08068	0.860174	1	http://jaspar.genereg.net/matrix/MA1066.1
abi4	MA0123.1	-1.08597	0.861347	1	http://jaspar.genereg.net/matrix/MA0123.1
CDF2	MA0973.1	-1.09958	0.864336	1	http://jaspar.genereg.net/matrix/MA0973.1
ERF9	MA1257.1	-1.10853	0.866268	1	http://jaspar.genereg.net/matrix/MA1257.1
PTF1	MA1282.1	-1.11106	0.866848	1	http://jaspar.genereg.net/matrix/MA1282.1
TCP17	MA1290.1	-1.1543	0.875951	1	http://jaspar.genereg.net/matrix/MA1290.1

At2g45680	MA1285.1	-1.15741	0.87652	1	http://jaspar.genereg.net/matrix/MA1285.1
DOF2.5	MA0977.1	-1.16939	0.878988	1	http://jaspar.genereg.net/matrix/MA0977.1
ERF15	MA1231.1	-1.17431	0.87992	1	http://jaspar.genereg.net/matrix/MA1231.1
ARALYDRAFT_495258	MA1095.1	-1.18204	0.881505	1	http://jaspar.genereg.net/matrix/MA1095.1
ARALYDRAFT_484486	MA1098.1	-1.18204	0.881505	1	http://jaspar.genereg.net/matrix/MA1098.1
TCP19	MA1063.1	-1.1846	0.881982	1	http://jaspar.genereg.net/matrix/MA1063.1
RAP212	MA1256.1	-1.2035	0.885695	1	http://jaspar.genereg.net/matrix/MA1256.1
bZIP911	MA0097.1	-1.20422	0.885823	1	http://jaspar.genereg.net/matrix/MA0097.1
TCP24	MA1286.1	-1.21019	0.887036	1	http://jaspar.genereg.net/matrix/MA1286.1
WRKY47	MA1312.1	-1.23355	0.89138	1	http://jaspar.genereg.net/matrix/MA1312.1
WRKY40	MA1085.2	-1.25064	0.894541	1	http://jaspar.genereg.net/matrix/MA1085.2
Os05g0497200	MA1034.1	-1.30163	0.903569	1	http://jaspar.genereg.net/matrix/MA1034.1
OsI_08196	MA1050.1	-1.3155	0.905889	1	http://jaspar.genereg.net/matrix/MA1050.1
TCP2	MA1064.1	-1.3186	0.906479	1	http://jaspar.genereg.net/matrix/MA1064.1
StBRC1	MA1410.1	-1.34667	0.911059	1	http://jaspar.genereg.net/matrix/MA1410.1
PHYPADRAFT_38837	MA1022.1	-1.34882	0.911403	1	http://jaspar.genereg.net/matrix/MA1022.1
ERF2	MA1262.1	-1.35623	0.912543	1	http://jaspar.genereg.net/matrix/MA1262.1
GATA6	MA1396.1	-1.37104	0.914897	1	http://jaspar.genereg.net/matrix/MA1396.1
TCP5	MA1067.1	-1.38947	0.917802	1	http://jaspar.genereg.net/matrix/MA1067.1
ERF104	MA1239.1	-1.39026	0.917832	1	http://jaspar.genereg.net/matrix/MA1239.1

TGA7	MA1070.1	-1.40289	0.919741	1	http://jaspar.genereg.net/matrix/MA1070.1
LEP	MA1246.1	-1.44643	0.92604	1	http://jaspar.genereg.net/matrix/MA1246.1
ABR1	MA1244.1	-1.44742	0.926189	1	http://jaspar.genereg.net/matrix/MA1244.1
TCP14	MA1283.1	-1.4702	0.929332	1	http://jaspar.genereg.net/matrix/MA1283.1
PHYPADRAFT_153324	MA0989.1	-1.47053	0.929404	1	http://jaspar.genereg.net/matrix/MA0989.1
RAP26	MA1221.1	-1.47102	0.929433	1	http://jaspar.genereg.net/matrix/MA1221.1
TCP20	MA1065.1	-1.4851	0.931314	1	http://jaspar.genereg.net/matrix/MA1065.1
WRKY50	MA1317.1	-1.49606	0.932735	1	http://jaspar.genereg.net/matrix/MA1317.1
ESE1	MA1264.1	-1.54376	0.938736	1	http://jaspar.genereg.net/matrix/MA1264.1
TGA6	MA1069.1	-1.55909	0.940548	1	http://jaspar.genereg.net/matrix/MA1069.1
WRKY60	MA1090.1	-1.5797	0.942968	1	http://jaspar.genereg.net/matrix/MA1090.1
WRKY20	MA1295.1	-1.59017	0.944139	1	http://jaspar.genereg.net/matrix/MA1295.1
PUCHI	MA1252.1	-1.59062	0.944212	1	http://jaspar.genereg.net/matrix/MA1252.1
WRKY45	MA1087.1	-1.6038	0.94568	1	http://jaspar.genereg.net/matrix/MA1087.1
WRKY6	MA1300.1	-1.61343	0.946729	1	http://jaspar.genereg.net/matrix/MA1300.1
BZIP60	MA0967.1	-1.62618	0.948086	1	http://jaspar.genereg.net/matrix/MA0967.1
ERF10	MA1240.1	-1.62831	0.948323	1	http://jaspar.genereg.net/matrix/MA1240.1
ESE3	MA1236.1	-1.65389	0.95098	1	http://jaspar.genereg.net/matrix/MA1236.1
ERF5	MA1225.1	-1.65609	0.951205	1	http://jaspar.genereg.net/matrix/MA1225.1
CDF3	MA0974.1	-1.68671	0.954252	1	http://jaspar.genereg.net/matrix/MA0974.1

BHLH112	MA0961.1	-1.69222	0.954782	1	http://jaspar.genereg.net/matrix/MA0961.1
ERF105	MA1000.2	-1.74699	0.959735	1	http://jaspar.genereg.net/matrix/MA1000.2
WRKY23	MA1080.1	-1.75606	0.960503	1	http://jaspar.genereg.net/matrix/MA1080.1
OJ1581_H09.2	MA1031.1	-1.76427	0.961221	1	http://jaspar.genereg.net/matrix/MA1031.1
bHLH80	MA1357.1	-1.79963	0.964128	1	http://jaspar.genereg.net/matrix/MA1357.1
WRKY21	MA1079.1	-1.80216	0.964297	1	http://jaspar.genereg.net/matrix/MA1079.1
AT2G33710	MA1245.1	-1.80334	0.964383	1	http://jaspar.genereg.net/matrix/MA1245.1
AT1G28160	MA1247.1	-1.84887	0.967805	1	http://jaspar.genereg.net/matrix/MA1247.1
WRKY59	MA1304.1	-1.85384	0.968163	1	http://jaspar.genereg.net/matrix/MA1304.1
TCP1	MA1284.1	-1.87537	0.969694	1	http://jaspar.genereg.net/matrix/MA1284.1
bHLH130	MA1358.1	-1.92349	0.972858	1	http://jaspar.genereg.net/matrix/MA1358.1
AT1G72740	MA1353.1	-1.92596	0.972983	1	http://jaspar.genereg.net/matrix/MA1353.1
At1g72010	MA1288.1	-1.93021	0.973251	1	http://jaspar.genereg.net/matrix/MA1288.1
WRKY57	MA1089.1	-1.94726	0.974291	1	http://jaspar.genereg.net/matrix/MA1089.1
WRKY25	MA1081.1	-1.96875	0.975546	1	http://jaspar.genereg.net/matrix/MA1081.1
WRKY75	MA1093.1	-1.97169	0.975719	1	http://jaspar.genereg.net/matrix/MA1093.1
WRKY2	MA1078.1	-1.99519	0.977025	1	http://jaspar.genereg.net/matrix/MA1078.1
TBP3	MA1355.1	-1.99821	0.97719	1	http://jaspar.genereg.net/matrix/MA1355.1
WRKY31	MA1307.1	-2.00451	0.977529	1	http://jaspar.genereg.net/matrix/MA1307.1
WRKY12	MA1075.1	-2.01138	0.977897	1	http://jaspar.genereg.net/matrix/MA1075.1

WRKY17	MA1299.1	-2.01565	0.978114	1	http://jaspar.genereg.net/matrix/MA1299.1
WRKY43	MA1086.1	-2.05228	0.979959	1	http://jaspar.genereg.net/matrix/MA1086.1
ARALYDRAFT_897773	MA1054.1	-2.06168	0.980426	1	http://jaspar.genereg.net/matrix/MA1054.1
At5g08330	MA1287.1	-2.20161	0.986181	1	http://jaspar.genereg.net/matrix/MA1287.1
bZIP50	MA1342.1	-2.20601	0.986331	1	http://jaspar.genereg.net/matrix/MA1342.1
WRKY28	MA1311.1	-2.24022	0.987487	1	http://jaspar.genereg.net/matrix/MA1311.1
WRKY27	MA1318.1	-2.28231	0.988788	1	http://jaspar.genereg.net/matrix/MA1318.1
WRKY38	MA1084.1	-2.32258	0.989924	1	http://jaspar.genereg.net/matrix/MA1084.1
WRKY8	MA1094.1	-2.32786	0.990069	1	http://jaspar.genereg.net/matrix/MA1094.1
WRKY29	MA1298.1	-2.3337	0.990217	1	http://jaspar.genereg.net/matrix/MA1298.1
TGA4	MA1335.1	-2.34407	0.990475	1	http://jaspar.genereg.net/matrix/MA1335.1
TGA3	MA1336.1	-2.34451	0.990485	1	http://jaspar.genereg.net/matrix/MA1336.1
TGA9	MA1348.1	-2.34724	0.990557	1	http://jaspar.genereg.net/matrix/MA1348.1
WRKY71	MA1316.1	-2.35002	0.990633	1	http://jaspar.genereg.net/matrix/MA1316.1
WRKY7	MA1313.1	-2.40746	0.991987	1	http://jaspar.genereg.net/matrix/MA1313.1
WRKY11	MA1306.1	-2.43191	0.992509	1	http://jaspar.genereg.net/matrix/MA1306.1
WRKY42	MA1310.1	-2.44406	0.992752	1	http://jaspar.genereg.net/matrix/MA1310.1
WRKY18	MA1077.1	-2.44404	0.992755	1	http://jaspar.genereg.net/matrix/MA1077.1
TCP16	MA0587.1	-2.45084	0.992889	1	http://jaspar.genereg.net/matrix/MA0587.1
P0510F09.23	MA1030.1	-2.46542	0.993171	1	http://jaspar.genereg.net/matrix/MA1030.1

WRKY33	MA1301.1	-2.47025	0.993262	1	http://jaspar.genereg.net/matrix/MA1301.1
WRKY15	MA1076.1	-2.48365	0.993519	1	http://jaspar.genereg.net/matrix/MA1076.1
WRKY30	MA1083.1	-2.48886	0.993611	1	http://jaspar.genereg.net/matrix/MA1083.1
WRKY63	MA1092.1	-2.51581	0.994075	1	http://jaspar.genereg.net/matrix/MA1092.1
WRKY22	MA1303.1	-2.55717	0.994739	1	http://jaspar.genereg.net/matrix/MA1303.1
WRKY3	MA1309.1	-2.73897	0.996926	1	http://jaspar.genereg.net/matrix/MA1309.1
WRKY46	MA1296.1	-2.75006	0.997029	1	http://jaspar.genereg.net/matrix/MA1296.1
WRKY48	MA1088.1	-2.76615	0.997177	1	http://jaspar.genereg.net/matrix/MA1088.1
WRKY14	MA1314.1	-2.79335	0.997399	1	http://jaspar.genereg.net/matrix/MA1314.1
WRKY55	MA1305.1	-2.82177	0.99762	1	http://jaspar.genereg.net/matrix/MA1305.1
WRKY65	MA1302.1	-2.87077	0.997958	1	http://jaspar.genereg.net/matrix/MA1302.1
TGA10	MA1346.1	-2.87583	0.997989	1	http://jaspar.genereg.net/matrix/MA1346.1
WRKY24	MA1315.1	-2.90954	0.998195	1	http://jaspar.genereg.net/matrix/MA1315.1
WRKY70	MA1308.1	-2.91604	0.998233	1	http://jaspar.genereg.net/matrix/MA1308.1
TRB2	MA1073.1	-2.9323	0.998322	1	http://jaspar.genereg.net/matrix/MA1073.1
WRKY26	MA1297.1	-3.00538	0.998677	1	http://jaspar.genereg.net/matrix/MA1297.1

Supplementary Table 9: Results of TFBS enrichment analysis according to Pscan on the upregulated genes. The first column reports the common name of the transcription factor, the Jaspar identifier of the corresponding TFBS is reported in the second column. Raw and normalized (using the Bonferroni correction) p-values for the enrichment are reported in the 3rd and 4th column, respectively.

TF_NAME	MATRIX_ID	Z_SCORE	P_VALUE	FDR	LINK
WRKY55	MA1305.1	4.97788	2.9619E-07	0.00014484	http://jaspar.genereg.net/matrix/MA1305.1
WRKY70	MA1308.1	4.95174	3.3909E-07	0.00016582	http://jaspar.genereg.net/matrix/MA1308.1
WRKY14	MA1314.1	4.92109	3.977E-07	0.00019447	http://jaspar.genereg.net/matrix/MA1314.1
WRKY65	MA1302.1	4.9194	4.0125E-07	0.00019621	http://jaspar.genereg.net/matrix/MA1302.1
WRKY29	MA1298.1	4.74172	9.8256E-07	0.00048047	http://jaspar.genereg.net/matrix/MA1298.1
WRKY22	MA1303.1	4.69007	1.2677E-06	0.00061989	http://jaspar.genereg.net/matrix/MA1303.1
WRKY33	MA1301.1	4.64303	1.6056E-06	0.00078515	http://jaspar.genereg.net/matrix/MA1301.1
WRKY26	MA1297.1	4.59623	2.011E-06	0.00098338	http://jaspar.genereg.net/matrix/MA1297.1
WRKY27	MA1318.1	4.59056	2.0565E-06	0.00100564	http://jaspar.genereg.net/matrix/MA1318.1
WRKY3	MA1309.1	4.5808	2.1706E-06	0.0010614	http://jaspar.genereg.net/matrix/MA1309.1
WRKY71	MA1316.1	4.46708	3.724E-06	0.00182104	http://jaspar.genereg.net/matrix/MA1316.1
WRKY46	MA1296.1	4.46455	3.7491E-06	0.0018333	http://jaspar.genereg.net/matrix/MA1296.1
WRKY40	MA1085.2	4.42655	4.5048E-06	0.00220283	http://jaspar.genereg.net/matrix/MA1085.2
WRKY28	MA1311.1	4.28989	8.4332E-06	0.00412381	http://jaspar.genereg.net/matrix/MA1311.1
WRKY20	MA1295.1	4.1178	1.8233E-05	0.00891579	http://jaspar.genereg.net/matrix/MA1295.1
AGL3	MA0001.2	3.95204	3.7115E-05	0.01814904	http://jaspar.genereg.net/matrix/MA0001.2
WRKY11	MA1306.1	3.91826	4.2311E-05	0.02069018	http://jaspar.genereg.net/matrix/MA1306.1
WRKY45	MA1087.1	3.91758	4.2769E-05	0.02091394	http://jaspar.genereg.net/matrix/MA1087.1
WRKY24	MA1315.1	3.85233	5.6017E-05	0.02739212	http://jaspar.genereg.net/matrix/MA1315.1
WRKY57	MA1089.1	3.73956	8.8055E-05	0.04305894	http://jaspar.genereg.net/matrix/MA1089.1
WRKY7	MA1313.1	3.6967	0.00010426	0.05098167	http://jaspar.genereg.net/matrix/MA1313.1
bHLH80	MA1357.1	3.58603	0.00015927	0.07788254	http://jaspar.genereg.net/matrix/MA1357.1
WRKY47	MA1312.1	3.55294	0.0001833	0.08963566	http://jaspar.genereg.net/matrix/MA1312.1
WRKY42	MA1310.1	3.5407	0.00019204	0.09390756	http://jaspar.genereg.net/matrix/MA1310.1
WRKY17	MA1299.1	3.51916	0.0002081	0.10176286	http://jaspar.genereg.net/matrix/MA1299.1
WRKY31	MA1307.1	3.50299	0.00022112	0.10812964	http://jaspar.genereg.net/matrix/MA1307.1
WRKY6	MA1300.1	3.4861	0.00023604	0.11542307	http://jaspar.genereg.net/matrix/MA1300.1
LEC2	MA0581.1	3.47132	0.00024786	0.1212055	http://jaspar.genereg.net/matrix/MA0581.1

WRKY59	MA1304.1	3.43508	0.00028561	0.13966525	http://jaspar.genereg.net/matrix/MA1304.1
bHLH130	MA1358.1	3.15379	0.00077155	0.37728648	http://jaspar.genereg.net/matrix/MA1358.1
ATHB21	MA1213.1	3.15131	0.0007902	0.38640976	http://jaspar.genereg.net/matrix/MA1213.1
WRKY38	MA1084.1	3.14291	0.00081135	0.39674868	http://jaspar.genereg.net/matrix/MA1084.1
ATHB7	MA0954.2	3.08487	0.00099467	0.48639412	http://jaspar.genereg.net/matrix/MA0954.2
WRKY23	MA1080.1	3.06523	0.00105762	0.51717618	http://jaspar.genereg.net/matrix/MA1080.1
ATHB40	MA1214.1	2.98305	0.00139584	0.68256576	http://jaspar.genereg.net/matrix/MA1214.1
WRKY50	MA1317.1	2.97088	0.00144683	0.70749987	http://jaspar.genereg.net/matrix/MA1317.1
WRKY43	MA1086.1	2.86896	0.00200561	0.98074329	http://jaspar.genereg.net/matrix/MA1086.1
WRKY30	MA1083.1	2.85948	0.00206677	1.01065053	http://jaspar.genereg.net/matrix/MA1083.1
ATHB-51	MA0952.1	2.83197	0.00226379	1	http://jaspar.genereg.net/matrix/MA0952.1
WRKY75	MA1093.1	2.81586	0.00237241	1	http://jaspar.genereg.net/matrix/MA1093.1
NAC92	MA1044.1	2.78844	0.00259648	1	http://jaspar.genereg.net/matrix/MA1044.1
ABI3	MA0564.1	2.7215	0.00317352	1	http://jaspar.genereg.net/matrix/MA0564.1
WRKY21	MA1079.1	2.66457	0.00376398	1	http://jaspar.genereg.net/matrix/MA1079.1
WRKY8	MA1094.1	2.55244	0.00524141	1	http://jaspar.genereg.net/matrix/MA1094.1
AHL12	MA0932.1	2.54916	0.00532608	1	http://jaspar.genereg.net/matrix/MA0932.1
ATHB-6	MA0953.1	2.50436	0.00603739	1	http://jaspar.genereg.net/matrix/MA0953.1
WRKY18	MA1077.1	2.4327	0.00736469	1	http://jaspar.genereg.net/matrix/MA1077.1
SPL8	MA0578.1	2.36697	0.00883142	1	http://jaspar.genereg.net/matrix/MA0578.1
WRKY12	MA1075.1	2.36512	0.00885064	1	http://jaspar.genereg.net/matrix/MA1075.1
WRKY15	MA1076.1	2.35114	0.00919719	1	http://jaspar.genereg.net/matrix/MA1076.1
ATHB23	MA1327.1	2.29965	0.0106039	1	http://jaspar.genereg.net/matrix/MA1327.1
BHLH112	MA0961.1	2.29354	0.0106641	1	http://jaspar.genereg.net/matrix/MA0961.1
ATHB18	MA1211.1	2.26585	0.011566	1	http://jaspar.genereg.net/matrix/MA1211.1
HAT5	MA0008.2	2.25847	0.0118015	1	http://jaspar.genereg.net/matrix/MA0008.2
WRKY48	MA1088.1	2.24453	0.0122115	1	http://jaspar.genereg.net/matrix/MA1088.1
At3g09600	MA1182.1	2.23332	0.0126021	1	http://jaspar.genereg.net/matrix/MA1182.1
ATHB24	MA1330.1	2.20309	0.0136061	1	http://jaspar.genereg.net/matrix/MA1330.1

RVE1	MA1184.1	2.18296	0.0143404	1	http://jaspar.genereg.net/matrix/MA1184.1
EDT1	MA0990.1	2.16889	0.0148719	1	http://jaspar.genereg.net/matrix/MA0990.1
HAT2	MA1198.1	2.13922	0.0160142	1	http://jaspar.genereg.net/matrix/MA1198.1
At5g52660	MA1183.1	2.12018	0.0168065	1	http://jaspar.genereg.net/matrix/MA1183.1
ATHB20	MA1209.1	2.09273	0.0179773	1	http://jaspar.genereg.net/matrix/MA1209.1
FUS3	MA0565.1	2.08099	0.01841	1	http://jaspar.genereg.net/matrix/MA0565.1
WRKY63	MA1092.1	2.06257	0.0193486	1	http://jaspar.genereg.net/matrix/MA1092.1
ATHB13	MA1212.1	2.04018	0.0204283	1	http://jaspar.genereg.net/matrix/MA1212.1
WRKY25	MA1081.1	2.03669	0.0205821	1	http://jaspar.genereg.net/matrix/MA1081.1
SPL3	MA0577.1	2.02786	0.0210154	1	http://jaspar.genereg.net/matrix/MA0577.1
ATHB53	MA1215.1	2.02662	0.0210785	1	http://jaspar.genereg.net/matrix/MA1215.1
WRKY62	MA1091.1	2.00824	0.0220242	1	http://jaspar.genereg.net/matrix/MA1091.1
AHL25	MA0934.1	2.00402	0.0223352	1	http://jaspar.genereg.net/matrix/MA0934.1
At1g49010	MA1186.1	2.00109	0.0224851	1	http://jaspar.genereg.net/matrix/MA1186.1
ATHB4	MA1406.1	1.99617	0.0227022	1	http://jaspar.genereg.net/matrix/MA1406.1
WRKY2	MA1078.1	1.98279	0.0234172	1	http://jaspar.genereg.net/matrix/MA1078.1
TGA6	MA1069.1	1.97229	0.023923	1	http://jaspar.genereg.net/matrix/MA1069.1
AG	MA0005.2	1.97199	0.0240421	1	http://jaspar.genereg.net/matrix/MA0005.2
ATHB-5	MA0110.2	1.97083	0.0240591	1	http://jaspar.genereg.net/matrix/MA0110.2
TGA9	MA1348.1	1.92391	0.0268456	1	http://jaspar.genereg.net/matrix/MA1348.1
LCL1	MA1187.1	1.91996	0.0271829	1	http://jaspar.genereg.net/matrix/MA1187.1
HDG1	MA1369.1	1.905	0.0281007	1	http://jaspar.genereg.net/matrix/MA1369.1
squamosa	MA0082.1	1.88762	0.0292603	1	http://jaspar.genereg.net/matrix/MA0082.1
At4g01280	MA1190.1	1.88546	0.0294066	1	http://jaspar.genereg.net/matrix/MA1190.1
WRKY60	MA1090.1	1.88447	0.0294302	1	http://jaspar.genereg.net/matrix/MA1090.1
AT3G10113	MA1191.1	1.88091	0.0296956	1	http://jaspar.genereg.net/matrix/MA1191.1
At5g08520	MA1399.1	1.87801	0.0298333	1	http://jaspar.genereg.net/matrix/MA1399.1
At2g38090	MA1193.1	1.85094	0.0318046	1	http://jaspar.genereg.net/matrix/MA1193.1
At3g11280	MA1188.1	1.84455	0.0322318	1	http://jaspar.genereg.net/matrix/MA1188.1

KAN2	MA1383.1	1.82895	0.0334308	1	http://jaspar.genereg.net/matrix/MA1383.1
TGA7	MA1070.1	1.78758	0.0365012	1	http://jaspar.genereg.net/matrix/MA1070.1
At5g58900	MA1192.1	1.77921	0.0372947	1	http://jaspar.genereg.net/matrix/MA1192.1
ANL2	MA1375.1	1.76434	0.0384837	1	http://jaspar.genereg.net/matrix/MA1375.1
AHL20	MA0933.1	1.7646	0.0385364	1	http://jaspar.genereg.net/matrix/MA0933.1
bZIP14	MA1407.1	1.74773	0.0397752	1	http://jaspar.genereg.net/matrix/MA1407.1
EPR1	MA1401.1	1.73681	0.0408798	1	http://jaspar.genereg.net/matrix/MA1401.1
At5g47390	MA1398.1	1.72328	0.0421051	1	http://jaspar.genereg.net/matrix/MA1398.1
AT1G76880	MA1366.1	1.7123	0.0430077	1	http://jaspar.genereg.net/matrix/MA1366.1
AT5G61620	MA1189.1	1.69585	0.0445927	1	http://jaspar.genereg.net/matrix/MA1189.1
SGR5	MA1159.1	1.67475	0.046597	1	http://jaspar.genereg.net/matrix/MA1159.1
ATHB-9	MA0573.1	1.65932	0.0480593	1	http://jaspar.genereg.net/matrix/MA0573.1
ATHB25	MA1329.1	1.65682	0.0483547	1	http://jaspar.genereg.net/matrix/MA1329.1
CCA1	MA0972.1	1.623	0.0519341	1	http://jaspar.genereg.net/matrix/MA0972.1
SEP1	MA0584.1	1.60415	0.0538837	1	http://jaspar.genereg.net/matrix/MA0584.1
HAT22	MA1210.1	1.57745	0.0568959	1	http://jaspar.genereg.net/matrix/MA1210.1
AT5G56840	MA1195.1	1.57425	0.0573224	1	http://jaspar.genereg.net/matrix/MA1195.1
AGL25	MA1200.1	1.55124	0.0600726	1	http://jaspar.genereg.net/matrix/MA1200.1
TGA10	MA1346.1	1.53091	0.0623801	1	http://jaspar.genereg.net/matrix/MA1346.1
AGL6	MA1205.1	1.51969	0.0637994	1	http://jaspar.genereg.net/matrix/MA1205.1
AT2G20110	MA1380.1	1.47206	0.0701136	1	http://jaspar.genereg.net/matrix/MA1380.1
ATHB33	MA1326.1	1.46065	0.0715909	1	http://jaspar.genereg.net/matrix/MA1326.1
NAC025	MA0935.1	1.45783	0.071923	1	http://jaspar.genereg.net/matrix/MA0935.1
TGA3	MA1336.1	1.44185	0.0741152	1	http://jaspar.genereg.net/matrix/MA1336.1
ATHB-16	MA0951.1	1.44167	0.0742331	1	http://jaspar.genereg.net/matrix/MA0951.1
AGL13	MA1204.1	1.43517	0.0751245	1	http://jaspar.genereg.net/matrix/MA1204.1
FLC	MA0558.1	1.42108	0.0771595	1	http://jaspar.genereg.net/matrix/MA0558.1
Dof3	MA0021.1	1.42168	0.0772936	1	http://jaspar.genereg.net/matrix/MA0021.1
At1g74840	MA1397.1	1.40611	0.0793441	1	http://jaspar.genereg.net/matrix/MA1397.1

SVP	MA0555.1	1.40402	0.079639<br>9	1	http://jaspar.genereg.net/matrix/MA0555.1
ATHB-12	MA0950.1	1.35758	0.086757<br>9	1	http://jaspar.genereg.net/matrix/MA0950.1
LHY1	MA1185.1	1.34662	0.088574<br>6	1	http://jaspar.genereg.net/matrix/MA1185.1
AT2G40260	MA1385.1	1.31845	0.093242<br>9	1	http://jaspar.genereg.net/matrix/MA1385.1
SOL1	MA1379.1	1.31091	0.094544<br>4	1	http://jaspar.genereg.net/matrix/MA1379.1
At1g19000	MA1400.1	1.30755	0.094967<br>9	1	http://jaspar.genereg.net/matrix/MA1400.1
TSO1	MA1161.1	1.30491	0.095545<br>3	1	http://jaspar.genereg.net/matrix/MA1161.1
SPL12	MA1057.1	1.29868	0.096466<br>7	1	http://jaspar.genereg.net/matrix/MA1057.1
TCX2	MA1162.1	1.27638	0.100493	1	http://jaspar.genereg.net/matrix/MA1162.1
TGA1A	MA0129.1	1.26892	0.101491	1	http://jaspar.genereg.net/matrix/MA0129.1
At5g05790	MA1196.1	1.26268	0.102783	1	http://jaspar.genereg.net/matrix/MA1196.1
SPL9	MA1322.1	1.26274	0.102802	1	http://jaspar.genereg.net/matrix/MA1322.1
ATHB15	MA1026.2	1.25265	0.104693	1	http://jaspar.genereg.net/matrix/MA1026.2
HBI1	MA1025.1	1.25128	0.104877	1	http://jaspar.genereg.net/matrix/MA1025.1
SPL4	MA1058.1	1.23249	0.108352	1	http://jaspar.genereg.net/matrix/MA1058.1
AGL27	MA1012.1	1.21951	0.110776	1	http://jaspar.genereg.net/matrix/MA1012.1
IDD2	MA1373.1	1.17589	0.119325	1	http://jaspar.genereg.net/matrix/MA1373.1
MYB33	MA1391.1	1.1746	0.119538	1	http://jaspar.genereg.net/matrix/MA1391.1
AT3G10580	MA1194.1	1.15664	0.123154	1	http://jaspar.genereg.net/matrix/MA1194.1
NAC055	MA0937.1	1.14195	0.126195	1	http://jaspar.genereg.net/matrix/MA0937.1
MYB27	MA1292.1	1.08755	0.137824	1	http://jaspar.genereg.net/matrix/MA1292.1
AGL63	MA1203.1	1.06798	0.142194	1	http://jaspar.genereg.net/matrix/MA1203.1
bZIP50	MA1342.1	1.05313	0.145439	1	http://jaspar.genereg.net/matrix/MA1342.1
SOC1	MA0554.1	1.01749	0.153928	1	http://jaspar.genereg.net/matrix/MA0554.1
bHLH69	MA1363.1	1.01666	0.154151	1	http://jaspar.genereg.net/matrix/MA1363.1
IDD7	MA1374.1	1.016	0.154251	1	http://jaspar.genereg.net/matrix/MA1374.1
TGA5	MA1047.1	0.934116	0.17434	1	http://jaspar.genereg.net/matrix/MA1047.1
MYB56	MA1174.1	0.878338	0.189403	1	http://jaspar.genereg.net/matrix/MA1174.1
AT5G47660	MA1365.1	0.865403	0.192808	1	http://jaspar.genereg.net/matrix/MA1365.1

DYT1	MA0580.1	0.858361	0.194702	1	http://jaspar.genereg.net/matrix/MA0580.1
IDD4	MA1371.1	0.847346	0.197841	1	http://jaspar.genereg.net/matrix/MA1371.1
MYB113	MA1181.1	0.843813	0.198868	1	http://jaspar.genereg.net/matrix/MA1181.1
AP3	MA0556.1	0.840761	0.199728	1	http://jaspar.genereg.net/matrix/MA0556.1
GT2	MA1208.1	0.810197	0.208329	1	http://jaspar.genereg.net/matrix/MA1208.1
PIF5	MA0562.1	0.788124	0.21476	1	http://jaspar.genereg.net/matrix/MA0562.1
AT3G25990	MA1368.1	0.781425	0.216791	1	http://jaspar.genereg.net/matrix/MA1368.1
NAC080	MA0939.1	0.771334	0.219617	1	http://jaspar.genereg.net/matrix/MA0939.1
OBP1	MA1278.1	0.769953	0.220159	1	http://jaspar.genereg.net/matrix/MA1278.1
ZAP1	MA0589.1	0.767692	0.220808	1	http://jaspar.genereg.net/matrix/MA0589.1
NAC058	MA0938.1	0.762896	0.222213	1	http://jaspar.genereg.net/matrix/MA0938.1
NUC	MA1157.1	0.760942	0.222836	1	http://jaspar.genereg.net/matrix/MA1157.1
KAN4	MA1028.1	0.749418	0.226253	1	http://jaspar.genereg.net/matrix/MA1028.1
NAC043	MA1045.1	0.746446	0.227096	1	http://jaspar.genereg.net/matrix/MA1045.1
HMG-1	MA0044.1	0.744034	0.227734	1	http://jaspar.genereg.net/matrix/MA0044.1
STZ	MA1372.1	0.738036	0.229661	1	http://jaspar.genereg.net/matrix/MA1372.1
O2	MA1417.1	0.730946	0.231812	1	http://jaspar.genereg.net/matrix/MA1417.1
SPL13	MA1321.1	0.684878	0.246117	1	http://jaspar.genereg.net/matrix/MA1321.1
AT3G04030	MA1168.1	0.673358	0.249923	1	http://jaspar.genereg.net/matrix/MA1168.1
KAN1	MA1027.1	0.67287	0.249983	1	http://jaspar.genereg.net/matrix/MA1027.1
MYB65	MA1177.1	0.671856	0.250445	1	http://jaspar.genereg.net/matrix/MA1177.1
PHYPADRAFT_140773	MA0987.1	0.671438	0.250567	1	http://jaspar.genereg.net/matrix/MA0987.1
NTL9	MA1046.1	0.66245	0.253349	1	http://jaspar.genereg.net/matrix/MA1046.1
Gam1	MA0034.1	0.651096	0.256928	1	http://jaspar.genereg.net/matrix/MA0034.1
PBF	MA0064.1	0.650481	0.257286	1	http://jaspar.genereg.net/matrix/MA0064.1
IDD5	MA1370.1	0.648141	0.257975	1	http://jaspar.genereg.net/matrix/MA1370.1
PEND	MA0127.1	0.647196	0.25827	1	http://jaspar.genereg.net/matrix/MA0127.1
HAT1	MA1024.1	0.637162	0.261514	1	http://jaspar.genereg.net/matrix/MA1024.1
MYB118	MA1170.1	0.614847	0.268816	1	http://jaspar.genereg.net/matrix/MA1170.1

MGP	MA1158.1	0.612436	0.26968	1	http://jaspar.genereg.net/matrix/MA1158.1
MYB57	MA1293.1	0.602165	0.272977	1	http://jaspar.genereg.net/matrix/MA1293.1
TSAR1	MA1411.1	0.601476	0.273259	1	http://jaspar.genereg.net/matrix/MA1411.1
AGL16	MA1199.1	0.576946	0.281525	1	http://jaspar.genereg.net/matrix/MA1199.1
AGL55	MA1202.1	0.560075	0.28722	1	http://jaspar.genereg.net/matrix/MA1202.1
MYB4	MA1039.1	0.559936	0.287385	1	http://jaspar.genereg.net/matrix/MA1039.1
ATHB34	MA1328.1	0.55914	0.287572	1	http://jaspar.genereg.net/matrix/MA1328.1
BZIP60	MA0967.1	0.545862	0.292072	1	http://jaspar.genereg.net/matrix/MA0967.1
bZIP911	MA0097.1	0.546091	0.292092	1	http://jaspar.genereg.net/matrix/MA0097.1
ARF2	MA1206.1	0.534274	0.296106	1	http://jaspar.genereg.net/matrix/MA1206.1
dof4.2	MA1273.1	0.525161	0.299315	1	http://jaspar.genereg.net/matrix/MA1273.1
MNB1A	MA0053.1	0.50336	0.306985	1	http://jaspar.genereg.net/matrix/MA0053.1
JKD	MA1156.1	0.49713	0.309144	1	http://jaspar.genereg.net/matrix/MA1156.1
ABF3	MA0930.1	0.487118	0.312735	1	http://jaspar.genereg.net/matrix/MA0930.1
T11I18.17	MA0936.1	0.464715	0.320645	1	http://jaspar.genereg.net/matrix/MA0936.1
SPL11	MA1056.1	0.454079	0.324493	1	http://jaspar.genereg.net/matrix/MA1056.1
AT5G66940	MA1267.1	0.44733	0.326918	1	http://jaspar.genereg.net/matrix/MA1267.1
bZIP43	MA1339.1	0.437162	0.330581	1	http://jaspar.genereg.net/matrix/MA1339.1
TSAR2	MA1412.1	0.435292	0.331257	1	http://jaspar.genereg.net/matrix/MA1412.1
AGL15	MA0548.2	0.432204	0.332474	1	http://jaspar.genereg.net/matrix/MA0548.2
AT1G25550	MA1386.1	0.415079	0.338643	1	http://jaspar.genereg.net/matrix/MA1386.1
AIB	MA0959.1	0.389038	0.34824	1	http://jaspar.genereg.net/matrix/MA0959.1
BIM2	MA0965.1	0.38838	0.348583	1	http://jaspar.genereg.net/matrix/MA0965.1
Dof2	MA0020.1	0.387618	0.348817	1	http://jaspar.genereg.net/matrix/MA0020.1
MYB62	MA1294.1	0.383201	0.350441	1	http://jaspar.genereg.net/matrix/MA1294.1
GT3a	MA1207.1	0.382793	0.350644	1	http://jaspar.genereg.net/matrix/MA1207.1
MYB46	MA1040.1	0.356714	0.360321	1	http://jaspar.genereg.net/matrix/MA1040.1
TCP17	MA1290.1	0.354589	0.361067	1	http://jaspar.genereg.net/matrix/MA1290.1
NAC083	MA1043.1	0.354423	0.361145	1	http://jaspar.genereg.net/matrix/MA1043.1

GBF6	MA1334.1	0.333467	0.369065	1	http://jaspar.genereg.net/matrix/MA1334.1
MYB98	MA1392.1	0.323319	0.372962	1	http://jaspar.genereg.net/matrix/MA1392.1
AT2G28810	MA1272.1	0.320383	0.374077	1	http://jaspar.genereg.net/matrix/MA1272.1
MYC3	MA0568.1	0.30907	0.378386	1	http://jaspar.genereg.net/matrix/MA0568.1
DOF5.7	MA0984.1	0.304628	0.380039	1	http://jaspar.genereg.net/matrix/MA0984.1
SPL14	MA0586.2	0.302192	0.380954	1	http://jaspar.genereg.net/matrix/MA0586.2
ARALYDRAFT_496250	MA1096.1	0.300678	0.381509	1	http://jaspar.genereg.net/matrix/MA1096.1
DOF1.8	MA0981.1	0.300488	0.381689	1	http://jaspar.genereg.net/matrix/MA0981.1
AT1G13300	MA1387.1	0.297685	0.382718	1	http://jaspar.genereg.net/matrix/MA1387.1
bZIP910	MA0096.1	0.287822	0.386351	1	http://jaspar.genereg.net/matrix/MA0096.1
MYB55	MA1041.1	0.286328	0.387053	1	http://jaspar.genereg.net/matrix/MA1041.1
PIF3	MA0560.1	0.272933	0.392189	1	http://jaspar.genereg.net/matrix/MA0560.1
bZIP53	MA1341.1	0.264284	0.39552	1	http://jaspar.genereg.net/matrix/MA1341.1
BEE2	MA0956.1	0.263285	0.395967	1	http://jaspar.genereg.net/matrix/MA0956.1
TGA4	MA1335.1	0.261403	0.396626	1	http://jaspar.genereg.net/matrix/MA1335.1
PI	MA0559.1	0.256826	0.398439	1	http://jaspar.genereg.net/matrix/MA0559.1
MYC2	MA0566.1	0.232636	0.407823	1	http://jaspar.genereg.net/matrix/MA0566.1
PIF4	MA0561.1	0.225285	0.410705	1	http://jaspar.genereg.net/matrix/MA0561.1
AP1	MA0940.1	0.225155	0.410766	1	http://jaspar.genereg.net/matrix/MA0940.1
AT4G12670	MA1354.1	0.222296	0.411776	1	http://jaspar.genereg.net/matrix/MA1354.1
PHYPADRAFT_153324	MA0989.1	0.219107	0.413132	1	http://jaspar.genereg.net/matrix/MA0989.1
SIZF2	MA1405.1	0.207278	0.41766	1	http://jaspar.genereg.net/matrix/MA1405.1
bZIP44	MA1337.1	0.197102	0.421673	1	http://jaspar.genereg.net/matrix/MA1337.1
BHLH78	MA0963.1	0.1832	0.427142	1	http://jaspar.genereg.net/matrix/MA0963.1
AT5G02460	MA1281.1	0.169872	0.4324	1	http://jaspar.genereg.net/matrix/MA1281.1
Adof1	MA1277.1	0.16235	0.435346	1	http://jaspar.genereg.net/matrix/MA1277.1
bZIP42	MA1350.1	0.1507	0.439956	1	http://jaspar.genereg.net/matrix/MA1350.1
PHYPADRAFT_48267	MA1021.1	0.124166	0.450495	1	http://jaspar.genereg.net/matrix/MA1021.1
AGL1	MA0585.1	0.113841	0.454572	1	http://jaspar.genereg.net/matrix/MA0585.1

At5g08330	MA1287.1	0.105999	0.457659	1	http://jaspar.genereg.net/matrix/MA1287.1
MYB81	MA1175.1	0.0971718	0.461219	1	http://jaspar.genereg.net/matrix/MA1175.1
PTF1	MA1282.1	0.0921982	0.463162	1	http://jaspar.genereg.net/matrix/MA1282.1
AT3G24120	MA1388.1	0.0861731	0.465574	1	http://jaspar.genereg.net/matrix/MA1388.1
AT2G03500	MA1167.1	0.0693379	0.472293	1	http://jaspar.genereg.net/matrix/MA1167.1
AT4G37180	MA1164.1	0.0553943	0.477856	1	http://jaspar.genereg.net/matrix/MA1164.1
PHYPADRAFT_72483	MA1011.1	0.0413952	0.483458	1	http://jaspar.genereg.net/matrix/MA1011.1
SPT	MA1061.1	0.0356529	0.485752	1	http://jaspar.genereg.net/matrix/MA1061.1
BIM1	MA0964.1	0.0266057	0.489368	1	http://jaspar.genereg.net/matrix/MA0964.1
RAV1	MA0582.1	0.0250967	0.489962	1	http://jaspar.genereg.net/matrix/MA0582.1
TCP19	MA1063.1	0.0235418	0.490584	1	http://jaspar.genereg.net/matrix/MA1063.1
FaEOBII	MA1408.1	0.0220311	0.49119	1	http://jaspar.genereg.net/matrix/MA1408.1
bZIP52	MA1343.1	0.0143035	0.494279	1	http://jaspar.genereg.net/matrix/MA1343.1
AT1G14580	MA1160.1	-0.00222501	0.50089	1	http://jaspar.genereg.net/matrix/MA1160.1
SPL15	MA1320.1	-0.00804865	0.503219	1	http://jaspar.genereg.net/matrix/MA1320.1
At2g45680	MA1285.1	-0.0281447	0.511264	1	http://jaspar.genereg.net/matrix/MA1285.1
At1g72010	MA1288.1	-0.0298939	0.511963	1	http://jaspar.genereg.net/matrix/MA1288.1
AT5G45580	MA1163.1	-0.0307083	0.512272	1	http://jaspar.genereg.net/matrix/MA1163.1
MYB3R5	MA1172.1	-0.0454591	0.518175	1	http://jaspar.genereg.net/matrix/MA1172.1
OBP3	MA1274.1	-0.048583	0.519414	1	http://jaspar.genereg.net/matrix/MA1274.1
TCP7	MA1291.1	-0.049949	0.519981	1	http://jaspar.genereg.net/matrix/MA1291.1
MYB59	MA1042.1	-0.0528027	0.521107	1	http://jaspar.genereg.net/matrix/MA1042.1
TCP1	MA1284.1	-0.0533969	0.521352	1	http://jaspar.genereg.net/matrix/MA1284.1
BHLH13	MA0958.1	-0.0572107	0.52286	1	http://jaspar.genereg.net/matrix/MA0958.1
bZIP48	MA1345.1	-0.0747679	0.529876	1	http://jaspar.genereg.net/matrix/MA1345.1
MYB73	MA1394.1	-0.0814812	0.53254	1	http://jaspar.genereg.net/matrix/MA1394.1
MYC4	MA0569.1	-0.0957082	0.538201	1	http://jaspar.genereg.net/matrix/MA0569.1
TCP24	MA1286.1	-0.100044	0.539953	1	http://jaspar.genereg.net/matrix/MA1286.1

MYB111	MA1036.1	-0.105007	0.541912	1	http://jaspar.genereg.net/matrix/MA1036.1
ARR18	MA0948.1	-0.111673	0.544567	1	http://jaspar.genereg.net/matrix/MA0948.1
HMG-I/Y	MA0045.1	-0.115977	0.546286	1	http://jaspar.genereg.net/matrix/MA0045.1
BIM3	MA0966.1	-0.11701	0.546664	1	http://jaspar.genereg.net/matrix/MA0966.1
TCP14	MA1283.1	-0.129323	0.551591	1	http://jaspar.genereg.net/matrix/MA1283.1
F3A4.140	MA0575.1	-0.135816	0.554156	1	http://jaspar.genereg.net/matrix/MA0575.1
TCP3	MA1289.1	-0.137119	0.554682	1	http://jaspar.genereg.net/matrix/MA1289.1
bHLH77	MA1362.1	-0.155731	0.562	1	http://jaspar.genereg.net/matrix/MA1362.1
BHLH34	MA0962.1	-0.164011	0.565262	1	http://jaspar.genereg.net/matrix/MA0962.1
BHLH3	MA0957.1	-0.181413	0.57213	1	http://jaspar.genereg.net/matrix/MA0957.1
bZIP3	MA1340.1	-0.184655	0.573418	1	http://jaspar.genereg.net/matrix/MA1340.1
SPL3	MA1319.1	-0.194933	0.577481	1	http://jaspar.genereg.net/matrix/MA1319.1
POPTR_0002s00440g	MA0955.1	-0.212191	0.584217	1	http://jaspar.genereg.net/matrix/MA0955.1
MYB101	MA1173.1	-0.213761	0.5848	1	http://jaspar.genereg.net/matrix/MA1173.1
bZIP28	MA1344.1	-0.22153	0.587832	1	http://jaspar.genereg.net/matrix/MA1344.1
DOF5.6	MA0983.1	-0.229301	0.590841	1	http://jaspar.genereg.net/matrix/MA0983.1
PHYPADRAFT_143875	MA0988.1	-0.232188	0.591969	1	http://jaspar.genereg.net/matrix/MA0988.1
SPL5	MA1059.2	-0.25648	0.601461	1	http://jaspar.genereg.net/matrix/MA1059.2
AREB3	MA1338.1	-0.257016	0.601585	1	http://jaspar.genereg.net/matrix/MA1338.1
GATA15	MA1016.1	-0.264141	0.604427	1	http://jaspar.genereg.net/matrix/MA1016.1
PIF1	MA0552.1	-0.279091	0.610119	1	http://jaspar.genereg.net/matrix/MA0552.1
AT3G46070	MA1381.1	-0.286029	0.612913	1	http://jaspar.genereg.net/matrix/MA1381.1
UIF1	MA1413.1	-0.287438	0.613413	1	http://jaspar.genereg.net/matrix/MA1413.1
bHLH18	MA1361.1	-0.290412	0.614456	1	http://jaspar.genereg.net/matrix/MA1361.1
DOF5.3	MA1071.1	-0.314238	0.623549	1	http://jaspar.genereg.net/matrix/MA1071.1
MYB24	MA1037.1	-0.320749	0.626103	1	http://jaspar.genereg.net/matrix/MA1037.1
bHLH74	MA1360.1	-0.322875	0.626823	1	http://jaspar.genereg.net/matrix/MA1360.1
MYB3R4	MA1180.1	-0.331229	0.630082	1	http://jaspar.genereg.net/matrix/MA1180.1
ERF018	MA1048.1	-0.336773	0.632151	1	http://jaspar.genereg.net/matrix/MA1048.1

MYB3R1	MA1178.1	-0.338494	0.63283	1	http://jaspar.genereg.net/matrix/MA1178.1
PHYPADRAFT_38837	MA1022.1	-0.352267	0.637928	1	http://jaspar.genereg.net/matrix/MA1022.1
UNE10	MA1074.1	-0.356606	0.639551	1	http://jaspar.genereg.net/matrix/MA1074.1
TCP20	MA1065.1	-0.357962	0.640161	1	http://jaspar.genereg.net/matrix/MA1065.1
TRB2	MA1073.1	-0.405013	0.657659	1	http://jaspar.genereg.net/matrix/MA1073.1
SPL7	MA1060.1	-0.406957	0.658346	1	http://jaspar.genereg.net/matrix/MA1060.1
AT1G78700	MA1331.1	-0.463776	0.678922	1	http://jaspar.genereg.net/matrix/MA1331.1
GATA20	MA1324.1	-0.481374	0.685395	1	http://jaspar.genereg.net/matrix/MA1324.1
AT1G47655	MA1275.1	-0.492397	0.689158	1	http://jaspar.genereg.net/matrix/MA1275.1
TCP23	MA1066.1	-0.512939	0.696467	1	http://jaspar.genereg.net/matrix/MA1066.1
MYB3	MA1038.1	-0.51456	0.696977	1	http://jaspar.genereg.net/matrix/MA1038.1
AT5G29000	MA1389.1	-0.52141	0.699348	1	http://jaspar.genereg.net/matrix/MA1389.1
TBP3	MA1355.1	-0.526201	0.701023	1	http://jaspar.genereg.net/matrix/MA1355.1
BHLH104	MA0960.1	-0.530766	0.702562	1	http://jaspar.genereg.net/matrix/MA0960.1
OJ1058_F05.8	MA1033.1	-0.544163	0.707195	1	http://jaspar.genereg.net/matrix/MA1033.1
DOF2.4	MA0982.1	-0.546504	0.707994	1	http://jaspar.genereg.net/matrix/MA0982.1
Glyma19g26560.1	MA1019.1	-0.546489	0.70813	1	http://jaspar.genereg.net/matrix/MA1019.1
MYB77	MA1395.1	-0.54797	0.708579	1	http://jaspar.genereg.net/matrix/MA1395.1
ARALYDRAFT_495258	MA1095.1	-0.551972	0.709955	1	http://jaspar.genereg.net/matrix/MA1095.1
ARALYDRAFT_484486	MA1098.1	-0.551972	0.709955	1	http://jaspar.genereg.net/matrix/MA1098.1
LFY	MA0590.1	-0.561738	0.713405	1	http://jaspar.genereg.net/matrix/MA0590.1
AT1G69570	MA1268.1	-0.572467	0.716922	1	http://jaspar.genereg.net/matrix/MA1268.1
TCP15	MA1062.1	-0.573701	0.717421	1	http://jaspar.genereg.net/matrix/MA1062.1
ARR11	MA0946.1	-0.582059	0.720151	1	http://jaspar.genereg.net/matrix/MA0946.1
OBP4	MA1280.1	-0.582109	0.720226	1	http://jaspar.genereg.net/matrix/MA1280.1
OsI_08196	MA1050.1	-0.593928	0.724229	1	http://jaspar.genereg.net/matrix/MA1050.1
MYB119	MA1176.1	-0.597496	0.7254	1	http://jaspar.genereg.net/matrix/MA1176.1
PIF7	MA1364.1	-0.598251	0.725607	1	http://jaspar.genereg.net/matrix/MA1364.1
TCP5	MA1067.1	-0.602857	0.727271	1	http://jaspar.genereg.net/matrix/MA1067.1

GBF3	MA1351.1	-0.610051	0.729501	1	http://jaspar.genereg.net/matrix/MA1351.1
EmBP-1	MA0128.1	-0.618389	0.732241	1	http://jaspar.genereg.net/matrix/MA0128.1
dof4.5	MA1269.1	-0.638874	0.739033	1	http://jaspar.genereg.net/matrix/MA1269.1
bZIP68	MA0968.1	-0.64713	0.741609	1	http://jaspar.genereg.net/matrix/MA0968.1
DREB1E	MA0978.1	-0.651755	0.743334	1	http://jaspar.genereg.net/matrix/MA0978.1
ABI5	MA0931.1	-0.686264	0.754094	1	http://jaspar.genereg.net/matrix/MA0931.1
MYB15	MA0574.1	-0.692143	0.756118	1	http://jaspar.genereg.net/matrix/MA0574.1
bHLH31	MA1359.1	-0.692964	0.756206	1	http://jaspar.genereg.net/matrix/MA1359.1
ARALYDRAFT_897773	MA1054.1	-0.696434	0.757494	1	http://jaspar.genereg.net/matrix/MA1054.1
ABF2	MA0941.1	-0.700315	0.75852	1	http://jaspar.genereg.net/matrix/MA0941.1
bZIP16	MA1349.1	-0.700607	0.758605	1	http://jaspar.genereg.net/matrix/MA1349.1
BZR1	MA0550.2	-0.703115	0.759472	1	http://jaspar.genereg.net/matrix/MA0550.2
AT4G36780	MA1332.1	-0.711164	0.761937	1	http://jaspar.genereg.net/matrix/MA1332.1
TCP2	MA1064.1	-0.722455	0.765531	1	http://jaspar.genereg.net/matrix/MA1064.1
SMZ	MA0553.1	-0.735981	0.769702	1	http://jaspar.genereg.net/matrix/MA0553.1
AGL42	MA1201.1	-0.750843	0.774169	1	http://jaspar.genereg.net/matrix/MA1201.1
myb.Ph3	MA0054.1	-0.752811	0.774646	1	http://jaspar.genereg.net/matrix/MA0054.1
TCP16	MA0587.1	-0.756962	0.776001	1	http://jaspar.genereg.net/matrix/MA0587.1
DAG2	MA1271.1	-0.760001	0.776871	1	http://jaspar.genereg.net/matrix/MA1271.1
TRP1	MA1352.1	-0.765836	0.778714	1	http://jaspar.genereg.net/matrix/MA1352.1
TGA2	MA1068.1	-0.76577	0.778827	1	http://jaspar.genereg.net/matrix/MA1068.1
DRE1C	MA0985.1	-0.786954	0.784937	1	http://jaspar.genereg.net/matrix/MA0985.1
RAMOSA1	MA1416.1	-0.788666	0.785423	1	http://jaspar.genereg.net/matrix/MA1416.1
ARALYDRAFT_493022	MA1097.1	-0.797672	0.788039	1	http://jaspar.genereg.net/matrix/MA1097.1
RAX3	MA0576.1	-0.805966	0.790408	1	http://jaspar.genereg.net/matrix/MA0576.1
bZIP68	MA1347.1	-0.81853	0.793893	1	http://jaspar.genereg.net/matrix/MA1347.1
CMTA3	MA0970.1	-0.825702	0.796147	1	http://jaspar.genereg.net/matrix/MA0970.1
CMTA2	MA0969.1	-0.834058	0.798525	1	http://jaspar.genereg.net/matrix/MA0969.1
MYB70	MA1393.1	-0.847182	0.802074	1	http://jaspar.genereg.net/matrix/MA1393.1

AT5G67000	MA1261.1	-0.858609	0.805301	1	http://jaspar.genereg.net/matrix/MA1261.1
AT3G45610	MA1270.1	-0.863833	0.806704	1	http://jaspar.genereg.net/matrix/MA1270.1
AT4G18890	MA1333.1	-0.86668	0.807376	1	http://jaspar.genereg.net/matrix/MA1333.1
REF6	MA1415.1	-0.885359	0.812793	1	http://jaspar.genereg.net/matrix/MA1415.1
CDF3	MA0974.1	-0.912856	0.819765	1	http://jaspar.genereg.net/matrix/MA0974.1
DOF2.5	MA0977.1	-0.923333	0.822527	1	http://jaspar.genereg.net/matrix/MA0977.1
OsRR22	MA1409.1	-0.938991	0.826685	1	http://jaspar.genereg.net/matrix/MA1409.1
BPC5	MA1403.1	-0.949676	0.829419	1	http://jaspar.genereg.net/matrix/MA1403.1
ARF5	MA0943.1	-0.957081	0.831379	1	http://jaspar.genereg.net/matrix/MA0943.1
ARR2	MA0949.1	-0.963078	0.8328	1	http://jaspar.genereg.net/matrix/MA0949.1
COG1	MA1279.1	-0.974217	0.83551	1	http://jaspar.genereg.net/matrix/MA1279.1
CDF2	MA0973.1	-0.992444	0.839993	1	http://jaspar.genereg.net/matrix/MA0973.1
P0510F09.23	MA1030.1	-1.0584	0.855685	1	http://jaspar.genereg.net/matrix/MA1030.1
BPC1	MA1404.1	-1.08991	0.862652	1	http://jaspar.genereg.net/matrix/MA1404.1
TRP2	MA1356.1	-1.09596	0.864066	1	http://jaspar.genereg.net/matrix/MA1356.1
TCP4	MA1035.1	-1.09942	0.864872	1	http://jaspar.genereg.net/matrix/MA1035.1
PLT1	MA1377.1	-1.10149	0.86514	1	http://jaspar.genereg.net/matrix/MA1377.1
MYB1	MA1179.1	-1.10409	0.86574	1	http://jaspar.genereg.net/matrix/MA1179.1
ANT	MA0571.1	-1.11264	0.867659	1	http://jaspar.genereg.net/matrix/MA0571.1
HY5	MA0551.1	-1.12037	0.869134	1	http://jaspar.genereg.net/matrix/MA0551.1
GATA19	MA1323.1	-1.15658	0.876996	1	http://jaspar.genereg.net/matrix/MA1323.1
CBF1	MA1224.1	-1.1607	0.877708	1	http://jaspar.genereg.net/matrix/MA1224.1
GT-1	MA1020.1	-1.16694	0.879123	1	http://jaspar.genereg.net/matrix/MA1020.1
AT2G01060	MA1384.1	-1.16907	0.879296	1	http://jaspar.genereg.net/matrix/MA1384.1
ARR1	MA0945.1	-1.1763	0.880858	1	http://jaspar.genereg.net/matrix/MA0945.1
AT3G52440	MA1276.1	-1.18022	0.8816	1	http://jaspar.genereg.net/matrix/MA1276.1
AT1G76870	MA1367.1	-1.1931	0.884232	1	http://jaspar.genereg.net/matrix/MA1367.1
MYB105	MA1169.1	-1.20382	0.886238	1	http://jaspar.genereg.net/matrix/MA1169.1
SEP3	MA0563.1	-1.22594	0.890419	1	http://jaspar.genereg.net/matrix/MA0563.1

SPL1	MA1055.1	-1.23291	0.891752	1	http://jaspar.genereg.net/matrix/MA1055.1
E2FA	MA1414.1	-1.23992	0.893142	1	http://jaspar.genereg.net/matrix/MA1414.1
AIL7	MA1235.1	-1.24684	0.894331	1	http://jaspar.genereg.net/matrix/MA1235.1
AT3G12730	MA1166.1	-1.26656	0.897779	1	http://jaspar.genereg.net/matrix/MA1166.1
TGA1	MA0588.1	-1.27805	0.900078	1	http://jaspar.genereg.net/matrix/MA0588.1
DREB1G	MA1032.1	-1.29322	0.902593	1	http://jaspar.genereg.net/matrix/MA1032.1
ERF7	MA0993.1	-1.3381	0.910045	1	http://jaspar.genereg.net/matrix/MA0993.1
BPC6	MA1402.1	-1.33993	0.910347	1	http://jaspar.genereg.net/matrix/MA1402.1
ERF098	MA0999.1	-1.40489	0.920424	1	http://jaspar.genereg.net/matrix/MA0999.1
abi4	MA0123.1	-1.41061	0.921368	1	http://jaspar.genereg.net/matrix/MA0123.1
ARF8	MA0944.1	-1.43472	0.924927	1	http://jaspar.genereg.net/matrix/MA0944.1
ERF11	MA1001.1	-1.43876	0.925315	1	http://jaspar.genereg.net/matrix/MA1001.1
DREB1A	MA0971.1	-1.47329	0.9302	1	http://jaspar.genereg.net/matrix/MA0971.1
MYB52	MA1171.1	-1.49858	0.933528	1	http://jaspar.genereg.net/matrix/MA1171.1
GATA11	MA1014.1	-1.50557	0.934359	1	http://jaspar.genereg.net/matrix/MA1014.1
ARF3	MA1009.1	-1.51711	0.935908	1	http://jaspar.genereg.net/matrix/MA1009.1
AT1G68670	MA1390.1	-1.53954	0.938612	1	http://jaspar.genereg.net/matrix/MA1390.1
id1	MA0120.1	-1.54193	0.938894	1	http://jaspar.genereg.net/matrix/MA0120.1
AT1G72740	MA1353.1	-1.56151	0.941179	1	http://jaspar.genereg.net/matrix/MA1353.1
AT1G49560	MA1165.1	-1.56111	0.941223	1	http://jaspar.genereg.net/matrix/MA1165.1
StBRC1	MA1410.1	-1.579	0.943307	1	http://jaspar.genereg.net/matrix/MA1410.1
CBF4	MA1218.1	-1.58111	0.943561	1	http://jaspar.genereg.net/matrix/MA1218.1
GATA14	MA1325.1	-1.62946	0.94885	1	http://jaspar.genereg.net/matrix/MA1325.1
ERF096	MA0998.1	-1.6372	0.949575	1	http://jaspar.genereg.net/matrix/MA0998.1
ERF069	MA0997.1	-1.66375	0.952263	1	http://jaspar.genereg.net/matrix/MA0997.1
ARF1	MA0942.1	-1.66628	0.95261	1	http://jaspar.genereg.net/matrix/MA0942.1
ERF039	MA0995.1	-1.66816	0.952737	1	http://jaspar.genereg.net/matrix/MA0995.1
CBF2	MA1217.1	-1.6711	0.953089	1	http://jaspar.genereg.net/matrix/MA1217.1
BZR2	MA0549.1	-1.68795	0.954564	1	http://jaspar.genereg.net/matrix/MA0549.1

AT1G19210	MA1234.1	-1.70087	0.955872	1	http://jaspar.genereg.net/matrix/MA1234.1
PLT3	MA1378.1	-1.73004	0.958532	1	http://jaspar.genereg.net/matrix/MA1378.1
ERF8	MA0994.1	-1.75316	0.960524	1	http://jaspar.genereg.net/matrix/MA0994.1
OJ1581_H09.2	MA1031.1	-1.78363	0.963143	1	http://jaspar.genereg.net/matrix/MA1031.1
PHYPADRAFT_64121	MA1010.1	-1.79132	0.963706	1	http://jaspar.genereg.net/matrix/MA1010.1
RAP210	MA1249.1	-1.80241	0.96462	1	http://jaspar.genereg.net/matrix/MA1249.1
PHYPADRAFT_182268	MA1008.1	-1.8126	0.96535	1	http://jaspar.genereg.net/matrix/MA1008.1
CAMTA1	MA1197.1	-1.81763	0.96586	1	http://jaspar.genereg.net/matrix/MA1197.1
ARR10	MA0121.1	-1.88785	0.970834	1	http://jaspar.genereg.net/matrix/MA0121.1
ABF1	MA0570.1	-1.89279	0.971054	1	http://jaspar.genereg.net/matrix/MA0570.1
ERF13	MA1004.1	-1.92373	0.973078	1	http://jaspar.genereg.net/matrix/MA1004.1
AT3G57600	MA1242.1	-1.94887	0.974558	1	http://jaspar.genereg.net/matrix/MA1242.1
CRF4	MA0976.1	-1.95189	0.974773	1	http://jaspar.genereg.net/matrix/MA0976.1
PHYPADRAFT_173530	MA1007.1	-1.9811	0.976477	1	http://jaspar.genereg.net/matrix/MA1007.1
ERF6	MA1006.1	-2.10165	0.982395	1	http://jaspar.genereg.net/matrix/MA1006.1
ERF13	MA1254.1	-2.10818	0.982697	1	http://jaspar.genereg.net/matrix/MA1254.1
PHYPADRAFT_28324	MA1023.1	-2.11656	0.983064	1	http://jaspar.genereg.net/matrix/MA1023.1
CRF2	MA0975.1	-2.14359	0.984154	1	http://jaspar.genereg.net/matrix/MA0975.1
GATA6	MA1396.1	-2.1502	0.984398	1	http://jaspar.genereg.net/matrix/MA1396.1
ERF1B	MA0567.1	-2.20535	0.986447	1	http://jaspar.genereg.net/matrix/MA0567.1
AT2G44940	MA1229.1	-2.20996	0.986624	1	http://jaspar.genereg.net/matrix/MA1229.1
ARR14	MA0947.1	-2.21413	0.986776	1	http://jaspar.genereg.net/matrix/MA0947.1
ERF043	MA0996.1	-2.22037	0.986991	1	http://jaspar.genereg.net/matrix/MA0996.1
GATA10	MA1013.1	-2.23203	0.987374	1	http://jaspar.genereg.net/matrix/MA1013.1
AT1G36060	MA1263.1	-2.23906	0.987581	1	http://jaspar.genereg.net/matrix/MA1263.1
RAP2-3	MA1051.1	-2.25898	0.988206	1	http://jaspar.genereg.net/matrix/MA1051.1
ERF112	MA1002.1	-2.26611	0.988438	1	http://jaspar.genereg.net/matrix/MA1002.1
ERF4	MA0992.1	-2.30164	0.989442	1	http://jaspar.genereg.net/matrix/MA0992.1
AT4G18450	MA1228.1	-2.30793	0.98961	1	http://jaspar.genereg.net/matrix/MA1228.1

AT1G77200	MA1230.1	-2.3202	0.989991	1	http://jaspar.genereg.net/matrix/MA1230.1
AT2G33710	MA1245.1	-2.32482	0.99006	1	http://jaspar.genereg.net/matrix/MA1245.1
TINY	MA1220.1	-2.32689	0.990164	1	http://jaspar.genereg.net/matrix/MA1220.1
DEAR3	MA1376.1	-2.33796	0.990425	1	http://jaspar.genereg.net/matrix/MA1376.1
FAR1	MA1382.1	-2.34541	0.990619	1	http://jaspar.genereg.net/matrix/MA1382.1
ERF094	MA1049.1	-2.34983	0.990737	1	http://jaspar.genereg.net/matrix/MA1049.1
GATA12	MA1015.1	-2.38432	0.991565	1	http://jaspar.genereg.net/matrix/MA1015.1
ERF10	MA1240.1	-2.39678	0.991827	1	http://jaspar.genereg.net/matrix/MA1240.1
ERF109	MA1053.1	-2.40336	0.991981	1	http://jaspar.genereg.net/matrix/MA1053.1
RAP26	MA1221.1	-2.40506	0.992007	1	http://jaspar.genereg.net/matrix/MA1221.1
AT3G60490	MA1223.1	-2.44925	0.992964	1	http://jaspar.genereg.net/matrix/MA1223.1
DEAR5	MA1251.1	-2.45184	0.99299	1	http://jaspar.genereg.net/matrix/MA1251.1
DREB2C	MA0986.1	-2.46239	0.993197	1	http://jaspar.genereg.net/matrix/MA0986.1
RAP2-6	MA1052.1	-2.46446	0.993229	1	http://jaspar.genereg.net/matrix/MA1052.1
ERF008	MA0979.1	-2.49948	0.993869	1	http://jaspar.genereg.net/matrix/MA0979.1
ERF3	MA1005.1	-2.55494	0.994766	1	http://jaspar.genereg.net/matrix/MA1005.1
GATA8	MA1017.1	-2.56577	0.994935	1	http://jaspar.genereg.net/matrix/MA1017.1
GATA9	MA1018.1	-2.57774	0.995113	1	http://jaspar.genereg.net/matrix/MA1018.1
ERF15	MA1231.1	-2.57977	0.995122	1	http://jaspar.genereg.net/matrix/MA1231.1
DREB19	MA1243.1	-2.58096	0.99516	1	http://jaspar.genereg.net/matrix/MA1243.1
AT4G32800	MA1241.1	-2.59974	0.99542	1	http://jaspar.genereg.net/matrix/MA1241.1
DREB2	MA1258.1	-2.60069	0.995423	1	http://jaspar.genereg.net/matrix/MA1258.1
RAP2-10	MA0980.1	-2.6087	0.995523	1	http://jaspar.genereg.net/matrix/MA0980.1
ERF104	MA1239.1	-2.64048	0.995918	1	http://jaspar.genereg.net/matrix/MA1239.1
ERF5	MA1225.1	-2.67057	0.996262	1	http://jaspar.genereg.net/matrix/MA1225.1
AT1G01250	MA1259.1	-2.69186	0.996511	1	http://jaspar.genereg.net/matrix/MA1259.1
FHY3	MA0557.1	-2.7025	0.996613	1	http://jaspar.genereg.net/matrix/MA0557.1
AT3G16280	MA1253.1	-2.72184	0.996818	1	http://jaspar.genereg.net/matrix/MA1253.1
RAP211	MA1266.1	-2.77932	0.99732	1	http://jaspar.genereg.net/matrix/MA1266.1

ERF105	MA1000.2	-2.79331	0.997427	1	http://jaspar.genereg.net/matrix/MA1000.2
Os05g0497200	MA1034.1	-2.79746	0.997466	1	http://jaspar.genereg.net/matrix/MA1034.1
RAV1(var.2)	MA0583.1	-2.83078	0.997719	1	http://jaspar.genereg.net/matrix/MA0583.1
ABR1	MA1244.1	-2.83677	0.997757	1	http://jaspar.genereg.net/matrix/MA1244.1
ESE1	MA1264.1	-2.85525	0.997882	1	http://jaspar.genereg.net/matrix/MA1264.1
PUCHI	MA1252.1	-2.86699	0.997956	1	http://jaspar.genereg.net/matrix/MA1252.1
AT1G75490	MA1250.1	-2.87048	0.997985	1	http://jaspar.genereg.net/matrix/MA1250.1
ESE3	MA1236.1	-2.98103	0.99859	1	http://jaspar.genereg.net/matrix/MA1236.1
AT4G16750	MA1237.1	-2.99539	0.998655	1	http://jaspar.genereg.net/matrix/MA1237.1
AT1G28160	MA1247.1	-3.05013	0.998875	1	http://jaspar.genereg.net/matrix/MA1247.1
AT1G71450	MA1233.1	-3.1009	0.999053	1	http://jaspar.genereg.net/matrix/MA1233.1
AT4G28140	MA1232.1	-3.12812	0.999137	1	http://jaspar.genereg.net/matrix/MA1232.1
ERF2	MA1262.1	-3.13601	0.99916	1	http://jaspar.genereg.net/matrix/MA1262.1
LEP	MA1246.1	-3.20279	0.999332	1	http://jaspar.genereg.net/matrix/MA1246.1
RAP21	MA1216.1	-3.20209	0.999333	1	http://jaspar.genereg.net/matrix/MA1216.1
AT4G31060	MA1265.1	-3.21264	0.999358	1	http://jaspar.genereg.net/matrix/MA1265.1
CDC5	MA0579.1	-3.21749	0.999369	1	http://jaspar.genereg.net/matrix/MA0579.1
AT1G12630	MA1227.1	-3.25734	0.999453	1	http://jaspar.genereg.net/matrix/MA1227.1
RAP212	MA1256.1	-3.27783	0.999488	1	http://jaspar.genereg.net/matrix/MA1256.1
ERF38	MA1238.1	-3.29912	0.999526	1	http://jaspar.genereg.net/matrix/MA1238.1
AT1G22810	MA1255.1	-3.31442	0.999552	1	http://jaspar.genereg.net/matrix/MA1255.1
AT5G18450	MA1226.1	-3.34502	0.999599	1	http://jaspar.genereg.net/matrix/MA1226.1
ERF9	MA1257.1	-3.49254	0.999766	1	http://jaspar.genereg.net/matrix/MA1257.1
DREB26	MA1248.1	-3.64316	0.999869	1	http://jaspar.genereg.net/matrix/MA1248.1
AT1G77640	MA1260.1	-3.76533	0.999919	1	http://jaspar.genereg.net/matrix/MA1260.1
CEJ1	MA1219.1	-3.90539	0.999954	1	http://jaspar.genereg.net/matrix/MA1219.1
AT1G44830	MA1222.1	-4.32352	0.999993	1	http://jaspar.genereg.net/matrix/MA1222.1

Supplementary Table 10: Results of TFBS enrichment analysis according to Pscan on the downregulated genes. The first column reports the common name of the transcription factor, the Jaspar identifier of the corresponding TFBS is reported in the second column. Raw and normalized

(using the Bonferroni correction) p-values for the enrichment are reported in the 3rd and 4th column, respectively.

Primer	Sequence	Purpose
Atp_0204	GCTTGTTCTGATAGCACCAACACTAGCA	STK and *stk* genotyping
Atp_0561	GGAACTCAAAGAGTCTCCCATCAG	STK and *stk* genotyping
Atp_2785	GGCACGGTTTGAGCCATATAAC	*ces-4* genotyping
Atp_2786	GCATCACTGCCATTCCCATTGC	*ces-4* genotyping
Atp_2812	TAGCATCTGAATTTCATAACCAATCTCGATACAC	*ces-4* genotyping (with Atp_2785)
Atp_0206	GATGCACTCGAAATCAGCCAATTTTAGAC	*shp1* genotyping (with Atp_207)
Atp_0207	GTGACGGAAGGAGGGTTGACG	*shp1* genotyping
Atp_0208	GTCTACTGATGAGTTGTCACTAGG	*shp1* genotyping
Atp_0645	GTTCTTGGTAAAAACATACTTGCC	*shp2* genotyping
Atp_0646	CCCTTCTTTTTTTGGGATATTATTG	*shp2* genotyping
Atp_0647	AGCGGATAACAATTTCACACAGGA	*shp2* genotyping (with Atp_646)
Atp_4361	CCCGGAAACTCTCCAGACAGTAGTAACAA	*bee1* genotyping
Atp_4362	CCTTATAACATCCGGGCACCATATCTTGCA	*bee1* genotyping
Atp_3744	GGCAATCAGCTGTTGCCCGTCTCACTGGTG	*bee1* genotyping (with Atp_4362)
Atp_3851	GGGGACAAGTTTGTACAAAAAAGCAGGCTCGATGGCAAATTTCGAGAATCTTTC	CDS cloning of BEE1
Atp_3852	GGGGACCACTTTGTACAAGAAAGCTGGGTGTCAAAGGGACCATGTTGATAAATG	CDS cloning of BEE1
Atp_4363	GCAGAGGATGAAACAGAGCCAAGCATGAA	*bee2* genotyping
Atp_4364	GGAGGACCTGTGAAGTAAGCCTGAAACTAG	*bee2* genotyping
Atp_3744	GGCAATCAGCTGTTGCCCGTCTCACTGGTG	*bee2* genotyping (with Atp_4364)
Atp_4365	CTCTACCTCTTCTGCTCAAGTTTCCATAAA	*bee3* genotyping
Atp_4366	AATCATAGCAAACATCACCAGTCTTACGAG	*bee3* genotyping
Atp_3744	GGCAATCAGCTGTTGCCCGTCTCACTGGTG	*bee3* genotyping (with Atp_4366)
Atp_3723	GCGGCAAGTGCATGTTACGACCT	In situ probe of CES
Atp_3785	GGTAATGTTGAACTGAAATTAG	In situ probe of CES
Atp_3957	CCAGGATGCTACAAGGCAAT	*CES* qRT-PCR in *ces-4*
Atp_3958	ATCCGTTGGCTCAATATCCA	*CES* qRT-PCR in *ces-4*
Atp_3855	GGGGACAAGTTTGTACAAAAAAGCAGGCTCGATGGCACGGTTTGAGCCATATAAC	CDS cloning of *CES*
Atp_3856	GGGGACCACTTTGTACAAGAAAGCTGGGTGTCAAAAGGGTAATGTTGAACTG	CDS cloning of *CES*
Atp_4204	GGGGACAAGTTTGTACAAAAAAGCAGGCTTCATGGGAAGAGGAAAGATAGAAATAAAG	CDS cloning of *STK*
Atp_4205	GGGGACCACTTTGTACAAGAAAGCTGGGTTTATCCGAGATGAAGAATTTTCTTG	CDS cloning of *STK*
RT_147	CTGTTCACGGAACCCAATTC	*UBIQUITIN* housekeeping
RT_148	GGAAAAAGGTCTGACCGACA	*UBIQUITIN* housekeeping
RT_861	CTCAGG TATTGCAGACCGTATGAG	*ACTIN* housekeeping
RT_862	CTGGACCTGCTTCATCATACTCTG	*ACTIN* housekeeping
RT_2870	GTCCGATCACGACACGTAAA	qRT-PCR *AT3G26140*
RT_2871	GTCGATTCCAAACTCGCTAAGA	qRT-PCR *AT3G26140*

RT_2872	TGTCCATTTCCTCTGTCTCTTG	qRT-PCR *AT1G06080*
RT_2873	TAGGACTATGTGGGTCCCTATC	qRT-PCR *AT1G06080*
RT_2874	CCGCGGCTACAATCTTACTT	qRT-PCR *AT1G28710*
RT_2875	AGCCTCATGTCGTACCATTTC	qRT-PCR *AT1G28710*

Supplementary Table 11: Primers used in this research article.
